# SPEX: A modular end-to-end platform for high-plex tissue spatial omics analysis

**DOI:** 10.1093/gigascience/giaf090

**Published:** 2025-08-29

**Authors:** Xiao Li, Ximo Pechuan-Jorge, Tyler Risom, Conrad Foo, Alexander Prilipko, Artem Zubkov, Caleb Chan, Patrick Chang, Frank Peale, James Ziai, Sandra Rost, Derrek Hibar, Lisa McGinnis, Evgeniy Tabatsky, Xin Ye, Hector Corrada Bravo, Zhen Shi, Malgorzata Nowicka, Jon Scherdin, James Cowan, Jennifer Giltnane, Darya Orlova, Rajiv Jesudason

**Affiliations:** Genentech, Inc., South San Francisco 94080, CA, USA; Roche Diagnostics Solutions, Santa Clara, CA 95050, USA; Genentech, Inc., South San Francisco 94080, CA, USA; Revolution Medicines, Redwood City, CA 94063, USA; Genentech, Inc., South San Francisco 94080, CA, USA; Genentech, Inc., South San Francisco 94080, CA, USA; Genentech, Inc., South San Francisco 94080, CA, USA; Genentech, Inc., South San Francisco 94080, CA, USA; Genentech, Inc., South San Francisco 94080, CA, USA; Genentech, Inc., South San Francisco 94080, CA, USA; Genentech, Inc., South San Francisco 94080, CA, USA; Genentech, Inc., South San Francisco 94080, CA, USA; Genentech, Inc., South San Francisco 94080, CA, USA; Genentech, Inc., South San Francisco 94080, CA, USA; Genentech, Inc., South San Francisco 94080, CA, USA; Genentech, Inc., South San Francisco 94080, CA, USA; Cellazon Inc., Los Altos, CA 94024, USA; Genentech, Inc., South San Francisco 94080, CA, USA; Genentech, Inc., South San Francisco 94080, CA, USA; Genentech, Inc., South San Francisco 94080, CA, USA; Genentech, Inc., South San Francisco 94080, CA, USA; Genentech, Inc., South San Francisco 94080, CA, USA; Genentech, Inc., South San Francisco 94080, CA, USA; Roche Diagnostics Solutions, Santa Clara, CA 95050, USA; Genentech, Inc., South San Francisco 94080, CA, USA; GlaxoSmithKline, Philadelphia, PA 19104, USA; Genentech, Inc., South San Francisco 94080, CA, USA; Cellazon Inc., Los Altos, CA 94024, USA; Genentech, Inc., South San Francisco 94080, CA, USA

**Keywords:** open source, user friendly web platform, spatial proteomics, spatial transcriptomics, spatial analysis, clustering, segmentation, graphical user interface

## Abstract

Recent advancements in transcriptomics and proteomics have opened the possibility for spatially resolved molecular characterization of tissue architecture with the promise of enabling a deeper understanding of tissue biology in either homeostasis or disease. The wealth of data generated by these technologies has recently driven the development of a wide range of computational methods. These methods have the requirement of advanced coding fluency to be applied and integrated across the full spatial omics analysis process, thus presenting a hurdle for widespread adoption by the biology research community. To address this, we introduce SPEX (Spatial Expression Explorer), a web-based analysis platform that employs modular analysis pipeline design, accessible through a user-friendly interface. SPEX’s infrastructure allows for streamlined access to open-source image data management systems, analysis modules, and fully integrated data visualization solutions. Analysis modules include essential steps covering image processing, single-cell analysis, and spatial analysis. We demonstrate SPEX’s ability to facilitate the discovery of biological insights in spatially resolved omics datasets from healthy tissue to tumor samples.

## Introduction

Spatially resolved, highly multiplexed protein and RNA profiling of tissues at the cellular or subcellular level is essential for understanding the molecular components underlying tissue architecture and function [1]. This fine characterization enables the measurement of cellular heterogeneity and cell–cell interactions in the spatial dimension, which in turn brings us closer to identifying molecular determinants of tissue function. Hence, recently developed technologies, such as IMC [2], MIBI [3], CODEX [4], or MERFISH [5], that allow for the acquisition of spatially resolved proteomic and transcriptomic states of individual cells and tissues, hold great promise for advancing our understanding in many areas of cell and developmental biology. However, the widespread adoption of these technologies vitally depends on the development of analytical capabilities and infrastructure to readily extract biologically relevant conclusions from the complex data generated.

Spatially resolved omics data contain multiple layers of information, which in turn are derived from imaging data and the molecular measurements associated with it. Examples of said layers constitute cell morphology, patterns of protein and transcript expression, cell neighborhoods, or cell–cell communication at different spatial scales. To obtain this information, robust preprocessing and visualization methods are required, as well as ways of analyzing the omics information and integrating it with the image-derived properties [6]. The current software ecosystem developed in the past 5 years leverages several common analysis categories, including cell segmentation, clustering, and spatial analytics, all required for meaningful interpretation of spatially resolved omics data [7, 8].

However, currently existing tools and custom analysis protocols provide only fragmentary solutions to the spatial omics analytical workflow [9, 10]. None of them standing alone provide means for efficient data representation and preprocessing, interactive visualizations, and spatial relationship querying. As a result, most current end-to-end analytical strategies involve piecemeal workflows requiring data migration across numerous programs and coding proficiency. To our knowledge, the code-free platforms currently available are predominantly developed by commercial spatial omics platform vendors. These software solutions are user-friendly but often entail prohibitive licensing agreements and associated costs that hinder widespread use. Moreover, these commercial solutions demonstrate limitations in their applicability across various modalities or platforms. Every imaging, transcriptomic, and proteomic data type has unique interpretation requirements, undercutting the effectiveness of platforms optimized for specific vendor data types. Therefore, the scientific community’s demand is 2-fold: for an open-source solution to democratize access and foster enhanced collaboration and reproducibility, and for a flexible, comprehensive tool that can be tailored to different modalities and platforms without compromising user accessibility and functionality.

With these current challenges in mind, we developed SPEX (Spatial Expression Explorer), a modular analytics platform that covers a broad span of essential methods required to analyze spatial omics data. SPEX is an application that could readily be deployed on a cloud or on a local workstation. It has a unique architecture that allows users to build data modality-specific customized pipelines from SPEX’s novel analytics developments or via plugging in external open-source analysis modules. To support the needs of the cross-functional research community, we designed SPEX in a way that one could operate it either from the user interface (code-free environment) or via more involved hands-on coding. A detailed comparison of SPEX with existing tools is provided in Supplementary Table S1, assessing key aspects such as execution mode (GUI vs. code-based), target audience and prerequisites, supported data types, interactive image visualization, image preprocessing, single-cell segmentation, cell type clustering, spatial analysis, image data management, and data analytics visualization.

Here, as part of the SPEX software package, we implement extensible methods for image preprocessing, single-cell segmentation, segmentation postprocessing, single-cell clustering, cell–cell co-occurrence, niche/neighborhood analysis, and spatially informed functional analysis in the form of differential expression analysis and pathway enrichment analysis. SPEX is integrated with the open-sourced and widely adopted image management system OMERO [11] and also leverages an open-source spatial omics data visualization solution, Vitessce [12], as a fully integrated element of the graphical user interface.

As a proof of concept, we show SPEX appropriately characterizes the spatial and molecular configuration of tonsil tissue. We further demonstrate the robustness of these methods in more heterogeneous tumor microenvironments represented in pancreatic ductal adenocarcinoma (PDAC) and triple-negative breast cancer (TNBC). Moreover, we extended the analysis to identify new patterns in tumor-immune microenvironment composition. Lastly, in a spatial transcriptomics dataset, we applied spatially informed pathway analysis to a human lung cancer specimen and elucidated the pathways associated with immune attraction/avoidance in immune cells and tumor cells.

## Results

**Figure 1:**
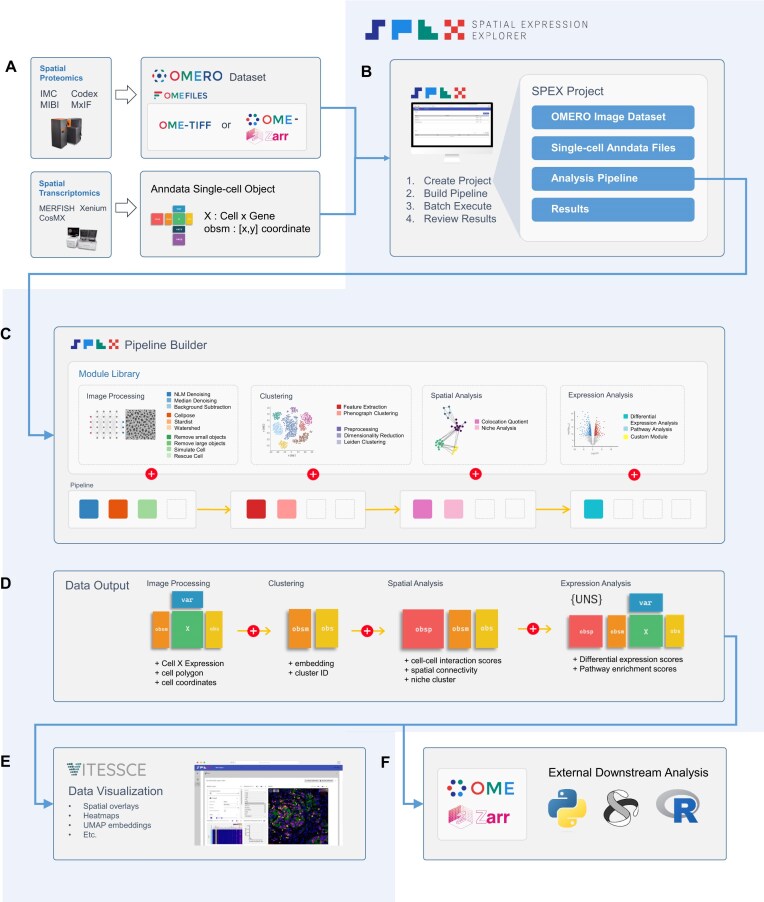
Graphical depiction of the Spatial Expression Explorer (SPEX) platform and analytical workflow. (A) Data can be input into SPEX as images or single-cell objects. Image data are managed and served into SPEX via the OMERO image management system. (B) Analytical projects in SPEX are executed as a 4-step process: create project, build pipeline, batch execute, and review results. Projects are containers for input data, editable pipelines, and study output data. (C) A modular pipeline builder is used to assemble an analytical routine. Modules are selected from the module library, covering image processing, segmentation, clustering, spatial analysis, and expression analysis. The individual modules are shown as multicolored rounded squares and can be added to the pipeline in a graphical manner. (D) Analysis data generated by the pipeline are stored as an Anndata object. As the pipeline proceeds, new elements are added to the Anndata object. (E) Processed datasets packaged as Anndata ZARR files can be reviewed using an integrated Vitessce dashboard. (F) Output data are compiled as an Anndata ZARR and can be exported for downstream analysis in a variety of platforms.

SPEX is a comprehensive spatial omics analysis platform implemented as a user-friendly web-based application with flexible plug-in analysis modules (Fig. 1, Supplementary Table S2). SPEX provides both infrastructure and quantitative analysis methods that allow for efficient storage (Fig. 1A), manipulation, and interactive visualization of spatial expression data (Fig. 1E). SPEX has an easy-to-use graphical user interface, which allows the wider research community to build analytical pipelines and visualize high-dimensional spatial data.

### SPEX analytical modules

For spatial proteomics modalities, SPEX can start with raw imaging data or single-cell objects with spatial coordinates. For spatial transcriptomics modalities, SPEX requires a single-cell object with spatial coordinates as input. Analysis can then be performed on these assets by leveraging a wide range of algorithms that can be categorized into 4 primary domains: image processing (spatial proteomics), single-cell clustering, cell–cell interaction spatial analysis, and spatially informed differential expression analysis and pathway analysis.

#### Image-processing modules

SPEX includes a modular pipeline to facilitate tissue-based single-cell segmentation (Fig. 1C). This generalized pipeline aims to accommodate a wide range of high-dimensional imaging modalities such as IMC, MIBI, cyclic IF, and spatial transcriptomics. Image processing is executed in a 4-step sequence with the ultimate goal of generating a cell by expression matrix in Anndata format for downstream single-cell and spatial analysis. These steps include image preprocessing, single-cell segmentation, postprocessing, and feature extraction. Each step contains a selection of modules, which can be linked together to address the particularities of a given image set. The adaptability of the pipeline was demonstrated by analyzing imaging data coming from a variety of high-dimensional imaging modalities. Each image set presented unique characteristics, varying in resolution, signal to noise, and structural attributes.

#### Clustering module

To enable the analysis of the high-plex omics data associated with the images, SPEX provides single-cell clustering modules that cover both intensity-based proteomics and count-based transcriptomics single-cell inputs. For transcriptomics, we rely on the suite of proven methods offered by the Pegasus package [13]. For proteomics, we also include the widely adopted graph-based clustering algorithm, PhenoGraph, which has recently been shown to be among the most robust to the curse of dimensionality compared to other high-dimensional clustering methods [14].

#### Spatial analysis module

Tissue architecture can be complex, with cell types forming spatial patterns that define particular domains where they might exert very different functions owing to the distinct local cellular context. These functions might be reflected in the associated gene or protein expression patterns. SPEX implements the Colocation Quotient (CLQ) module, as detailed in the Methods section, which integrates spatial coordinates of the identified cells with their respective cell types to stratify them into colocation or avoidance pattern between cell types. Further, given a set of spatially co-occurring cells, it is natural to ask whether these co-occurrence patterns are repeated throughout the tissue and what comprises such patterns or spatial niches. Clustering spatial co-occurrence features (in an unsupervised fashion) facilitates the identification of such spatial patterns or niches.

#### Spatial expression analysis module

The functional state of cells and/or their molecular action may be influenced by their spatial organization in tissue. This can be due to inclusion in higher-order functional structures or cell–cell mediated interactions. To facilitate quantification of spatially informed expression, SPEX includes both differential expression analysis and pathway analysis. These modules can take spatially informed cell categories as input.

### SPEX user interface

**Figure 2:**
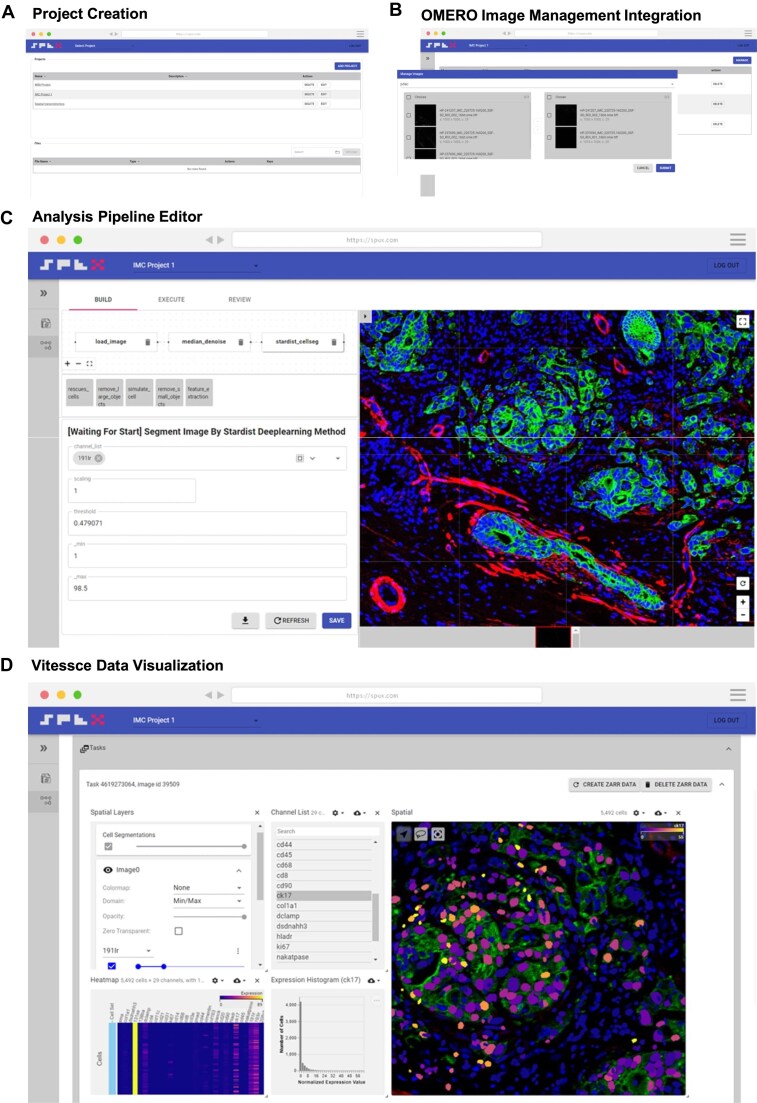
SPEX Graphical User Interface. (A) SPEX project creation user interface. Projects are containers for study data. (B) SPEX-OMERO integration highlighting navigation and selection OMERO hosted datasets and images within SPEX. (C) SPEX pipeline builder user interface. An intuitive 3-step process of building, executing, and reviewing the analysis displayed at the top of the interface. The user moves through each step in sequence. In the build section, the user is able to graphically build an analysis pipeline by selecting and chaining modules in sequence. A unique parameter section is displayed for each module. (D) Fully integrated Vitessce dashboard for visualizing results in spatial context.

The SPEX graphical user interface is designed to minimize spurious user options and visual overhead in an effort to increase platform intuitiveness. At a high level, the SPEX interface can be divided into 3 primary sections organized as sequential steps: project creation, data loading, and analysis development (Fig. 2). The analysis section contains sequential subsections covering pipeline building, batch execution, and data visualization (Fig. 2C). Critically, these application sections can be executed without the need for code scripting, ensuring a user-friendly and efficient analysis experience within the SPEX environment.

#### Project creation

After logging into SPEX, the first screen encountered by users is the SPEX project creation interface (Fig. 2A), where projects, serving as containers for study data, are initiated. The user can define a name and description for the project for future retrieval. This project can then be accessed and populated with omics data to be analyzed.

#### Data loading

SPEX supports analysis of 1 of 3 data formats: OMETIFF, OMEZARR, or H5AD (Anndata). OMETIFF and OMEZARR represent supported multiplex image formats while H5AD represents the supported single-cell data format. H5AD files can be loaded from local storage drives. The integration with an OMERO image management system (Fig. 2B) allows seamless visual navigation and selection of OMERO-hosted imaging data within SPEX. Individual images across OMERO datasets can be selected, concatenated, and downloaded into the SPEX project for downstream analysis.

#### Analysis development

Once a project is populated with image or single-cell data, the user can define an analysis pipeline. Several pipelines can exist for any given project if the user is interested in prototyping different analysis workflows. To develop an analytical workflow, users engage with the SPEX pipeline builder interface (Fig. 2C). The user progresses in the build section, enabling the graphical construction of analysis pipelines. In this phase, modules are selected from a library section and dragged into a visual pipeline map (Fig. 1C). The selection of modules is dependency-informed, where new modules can only be added to modules that generate prerequisite data, thus eliminating the potential for erroneous pipeline construction. Modules with user-defined parameters include graphical widgets such as slidebars and dropdown menus displayed in a window to facilitate code-free user parameterization. Once the user has constructed a pipeline and defined the parameters, they move onto execution. In the execution page of the pipeline builder, the user can send the data for batch processing. The status of the analysis jobs will be displayed for all images in the queue. When complete, an aggregated Anndata single-cell object housing data from all images is automatically generated and available for local download as a ZARR file. This ZARR file can be loaded into other external analysis routines in Python or R (Fig. 1F).

#### Visualization

The final step in the pipeline building interface is data visualization. Here, users can visualize analytical readouts using an integrated Vitessce spatial omics dashboard. Vitessce, as described by Keller et al. [12], is an advanced web-based platform designed for the interactive visualization of spatial omics data. The dashboard integrates a variety of visualization modalities, such as spatial plots, scatterplots, expression heatmaps, histograms, and more. All visualization panes are interactive and have cross-filtering functionality (Fig. 2D). As an example, a user can select a specific population of cells in the UMAP embedded space, and these same cells will be highlighted in the spatial plot atop the multiplex imaging data. The image pane can be zoomed and panned to further explore the tissue microenvironment. Together, these plots facilitate investigation of spatial patterns, cell clustering, and tissue organization at single-cell resolution.

### SPEX workflow on spatial proteomics data

#### SPEX identified structural composition of tonsil

In the previous sections, we illustrated the capabilities of the different modules that integrate SPEX. To demonstrate that SPEX analysis modules generate biologically meaningful results, we validated the SPEX workflow by analyzing a sample from human tonsil tissue, which has a well-defined cellular organization [15–17]. To achieve this, a 4$\mathrm{\mu }$m-thick section of formalin-fixed, paraffin-embedded (FFPE) tonsil tissue was stained with a panel of imaging mass cytometry (IMC) antibodies listed in Supplementary Table S3. The antibody panel was designed to simultaneously characterize the composition of the immune compartment, the spatial relationship between immune cells and stromal cells, and the interactions among cell subsets.

**Figure 3:**
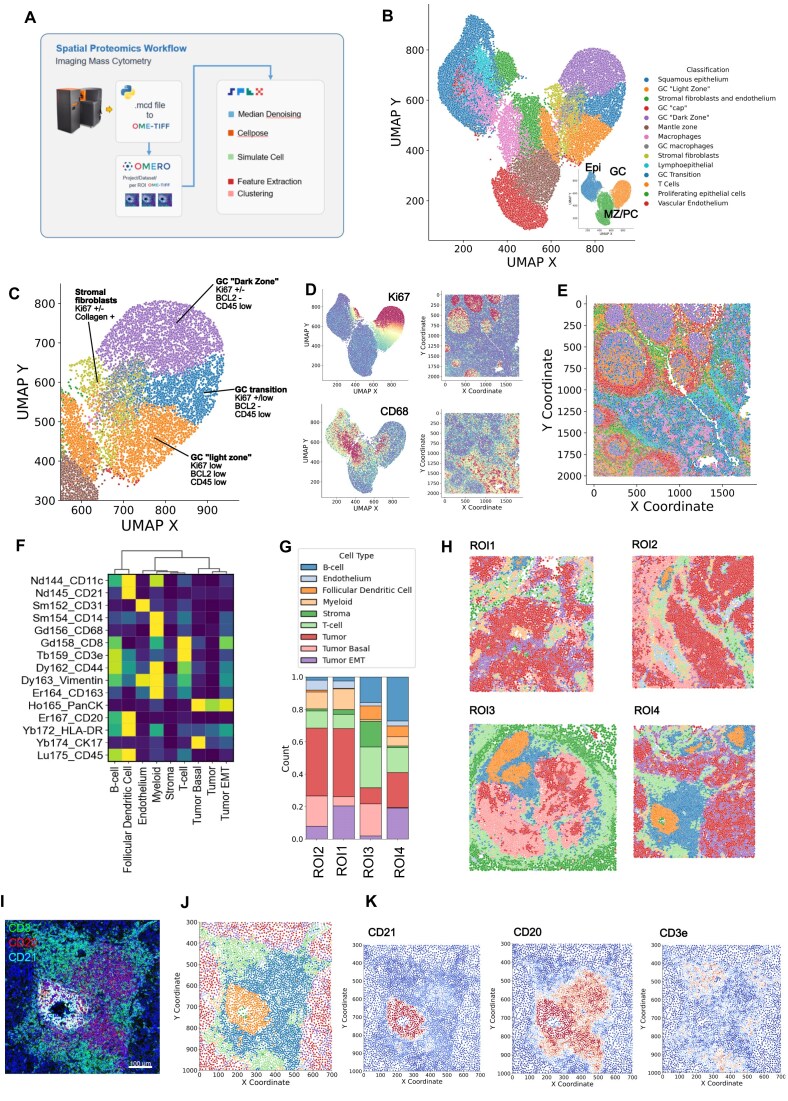
SPEX robustly identified structural composition of tonsil and PDAC tissue in IMC modality. (A) Graphical dataset description and SPEX spatial proteomics workflow modules highlighted in this figure. (B) UMAP embedding of tonsil single-cell data colored by pathologist-annotated cell type. Inset shows primary high-order tonsil structures (EPI = epithelial, GC = germinal Center, MZ/PC= marginal zone/paracortex). (C) Detail of the germinal center region of UMAP with pathologist-annotated functional zones. (D) Protein expression of Ki67 and CD68 color mapped to UMAP embedding and spatial domain. Warm colors denote high expressing regions. (E) The histologic tonsil image is color-coded by 14 structural hierarchy categories identified by the marker expression pattern. (F) PDAC single-cell clustering expression heatmap. (G) Cell-type composition of 4 individual IMC ROIs. (H) Spatial map of clustered cell types across 4 PDAC ROIs. (I) Multiplex IMC image showing detail of tertiary lymphoid structure (TLS). CD3 = green, CD20 = red, CD21 = cyan. (J) Detail spatial map of cell types in a TLS region. (K) Spatial distribution expression heatmap of CD21, CD20, and CD3e.

The stained tonsil sample was imaged with the Hyperion Imaging System and then preprocessed and analyzed using a SPEX pipeline utilizing most of SPEX modules (Fig. 3A). This pipeline applied to the tonsil tissue IMC images started with per cell segmentation using StarDist based on the nuclear histone H3 channel. Then, PhenoGraph was used for clustering the segmented cells according to their median marker expression levels of the different IMC channels. Clustering outcomes were visualized using UMAP, and cluster labels were assigned based on the IMC marker measurements, as well as positional data (x and y coordinates) of cells, revealing 3 well-defined lobes representing epithelial, follicular, and paracortical tissue compartments distinguished primarily by expression of cytokeratin, Ki67, and CD8, respectively (Fig. 3B), consistent with the composition of cells in these compartments. Within the individual UMAP regions, the location of closely related cell populations correlates with biologically relevant parameters. For example, we note distinct populations representing well-characterized functional zones of tonsil germinal centers. Germinal center light and dark zones can be distinguished across an axis of Ki67 and BCL2 expression.

We applied this same SPEX workflow of single-cell segmentation, feature extraction, data normalization, and single-cell phenotyping to 4 regions of interest in a PDAC patient tumor, revealing major cell types by PhenoGraph, including stromal cells, T cells, myeloid cells, B cells, Follicular dendriticcells (FDC), and a myriad of tumor cell states (Fig. 3F). Compositional analysis of these cell states per region of interest (ROI) revealed 2 of the 4 ROIs encompassed robust B-cell and FDC cell frequencies (Fig. 3G) in a spatially colocalized pattern (Fig. 3H). Upon further spatial interrogation of the molecular expression of CD3, CD20, and CD21 in these cells by image overlays (Fig. 3I) and spatial heatmaps (Fig. 3J–L), they appear to compile a tertiary lymphoid structure (TLS).

#### SPEX spatial proteomics pipeline enables novel single-cell spatial insights

**Figure 4:**
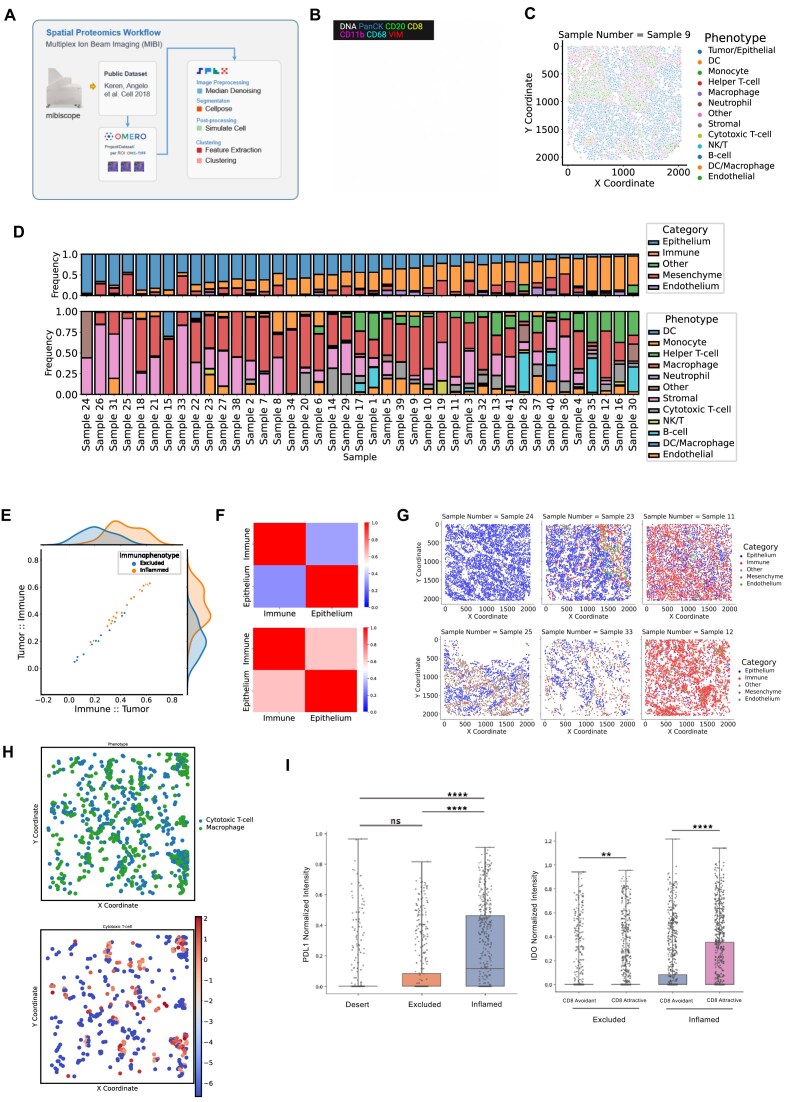
SPEX spatial analysis modules reveal immune cell state relationships to tumor/immune spatial architecture in a TNBC MIBI dataset. (A) Graphical dataset description and SPEX spatial proteomics workflow modules highlighted in this figure. (B) Representative image of TNBC patient tumor from the Keren cohort showing color overlays of DNA (white), pancytokeratin (PanCK, blue), CD20 (green), CD8 (yellow), CD11b (pink), CD68 (cyan), and vimentin (VIM, red); scale bar = 100 μm. (C) Cell phenotype map corresponding to B, showing 13 major cellular phenotypes by colored points. (D) Frequencies of major cell lineages (of all cells, top) and immune cell types (of total immune cells, bottom) are shown as stacked bar plots for each patient. (E) Scatterplot showing results of SPEX CLQ analysis module comparing the immune/tumor spatial enrichment versus tumor/immune spatial enrichment in each patient who had more than 10% immune cells (not desert). Based on these data, patients are assigned to an immune-inflamed (orange) class or an immune-excluded (blue) class using a GMM model. (F) Heatmaps showing the tumor/immune, tumor/tumor, immune/tumor, and immune/immune CLQ results in immune-excluded patients versus immune-inflamed patients. (G) Representative cell phenotype maps of 2 immune-desert tumors, 2 immune-excluded tumors, and 2 immune-inflamed tumors, showing the location of tumor (blue), immune (red), mesenchymal (brown), endothelial (green), and other (gray) cells. (H) A cell phenotype map (top) showing cytotoxic T cells (blue) and macrophages (green) is shown above a heatmap of the macrophage cells, colored by their CLQ macrophage/T-cell spatial enrichment score. (I) Boxplot showing the normalized expression of PDL1 in macrophages in immune-desert, immune-excluded, and immune-inflamed tumors; asterisks denote significance in the Kruskal–Wallis test. ****P  $<0.001$, ns = not significant. Boxplot showing the normalized expression of IDO1 in macrophages that are either CD8/T cell avoidant (CLQ $<0.5$) or CD8/T cell attractive (CLQ$>1.5$) in immune-excluded versus immune-inflamed tumors; Mann–Whitney test. Asterisks denote significance: **$P<0.01$, ****$P<0.001$.

Next we analyzed a public Multiplex Ion Beam Imaging (MIBI) dataset [3] to demonstrate the single-cell spatial analysis methods of the SPEX pipeline (Fig. 4A). This image set consisted of 41 field-of-view images with 36 channels. Being a nonoptical mass detection platform, it is common for raw images to include substantial noise when compared to traditional optical acquisition platforms. Therefore, median denoising was executed on channels utilized for segmentation (dsDNA, H3K9ac, H3K27me3), and channels were then merged and segmented for single cells using the Cellpose deep learning model [18]. Following cell phenotyping by unsupervised clustering, these SPEX modules generated cell calls that correctly match their molecular profiles and the published calls by the original work [3] (Fig. 4B–D). Through arranging the tumors from low to high immune cell frequency of total cells, we observe an increased diversity of immune cell types within the sample where samples with a lower density of immune cells are dominated by macrophages, and samples with higher density of immune cells have a mixed composition of lymphocytes, myeloid cells, and antigen-presenting cells (Fig. 4D). To further interrogate the spatial relationship between this variable immune compartment and the tumor cells, we employ the CLQ method under the SPEX spatial co-occurrence module. Briefly, the CLQ measures the co-occurrence or avoidance of cell-type pairs by looking at the local density of a target cell type at a fixed radius from each cell of the sample belonging to a reference cell type. That way, one can, for instance, query if target immune cells are colocating or avoiding tumor cells. We applied the CLQ module with the immune cell/tumor cell pair here in samples that had more than 10% immune cells (nondesert samples), revealing 2 major groups of samples: those with high immune/tumor cell spatial enrichment and those with low immune/tumor cell enrichment (Fig. 4E). These identified inflamed and excluded groupings aligned well with the visual representation of immunophenotyping presented in the images (Fig. 4F).

Having defined the tumor immune phenotypes with CLQ analysis as desert, excluded, or inflamed tumors, we explored potential gene expression patterns yielded by this classification. We observed a significant increase in PDL1 expression by tumor-associated macrophages in inflamed tumors, suggesting a relationship between tumor-immune spatial enrichment and immune checkpoint expression on tumor-associated macrophages (Fig. 4H) [19, 20]. We then further leveraged the CLQ module to interrogate macrophage phenotypes in these tumors by computing their pairwise spatial enrichment with all other cell types. Interestingly, this combination of CLQ and expression analysis revealed that macrophages that are spatially enriched (attractive) with CD8^+^ cytotoxic T cells (Fig. 4G) had significantly higher IDO1 expression compared to macrophages that are spatially isolated (avoidant) from these T cells (Fig. 4I). This relationship was observed in excluded tumors and further enriched in inflamed tumors, suggesting IDO1 expression by macrophages may play an immunosuppressive role specifically in the inflamed subset of patients. While conclusions on the significance of these observations would require their validation by orthogonal assays, this demonstrates the utility of the single-cell annotation and spatial enrichment SPEX modules for analyses of the Tumor Microenvironment (TME) and hypothesis generation [21].

### SPEX workflow on spatial transcriptomics data

The availability of spatial resolution enhances the meaningful interpretation of cellular interactions and signaling events. The CLQ module calculates descriptive statistics of spatial avoidance and attraction at both the single-cell and cell cluster levels. Gene and pathway expression states can be correlated with these statistics to provide insight into the effects of spatial co-occurrence on the transcriptome.

**Figure 5:**
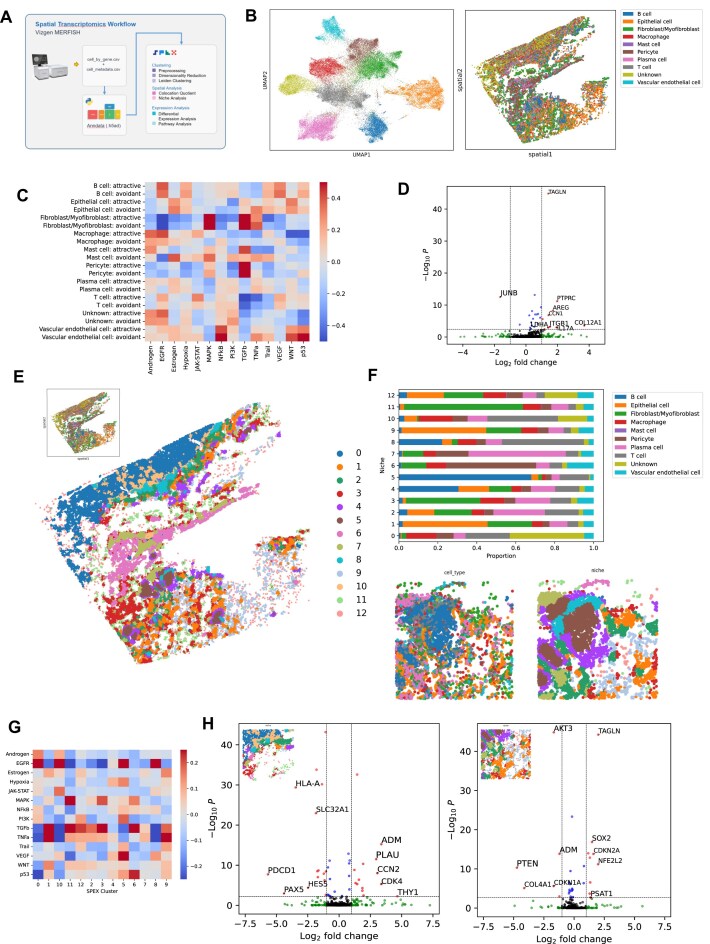
SPEX spatial transcriptomics analysis identifies immune cell niches. (A) Graphical dataset description and SPEX spatial transcriptomics workflow modules highlighted in this figure. (B) Cell phenotype map in both gene expression (UMAP, left) and spatial (right) coordinates. (C) PROGENy pathway enrichment scores for each cell type cluster, split by colocalization with T cells. Colocalization was determined using the SPEX CLQ analysis module. Scores were calculated by fitting a multilinear model to the gene expression of each cell given the pathway weights per gene. The average score per cluster is shown here. (D) Volcano plot showing differentially expressed genes for T cells based on colocalization with epithelial cells. (E) Spatial map of cell niches. (F) Cell phenotype composition of detected niches with detail spatial maps showing phenotype spatial arrangement in niches. (G) PROGENy pathway enrichment scores for each spatial niche. (H) Volcano plot showing differential expression between T cells in spatial niche 10 versus spatial niche 8 (B-cell enriched) (left) and volcano plot showing differential expression between epithelial cells in spatial niche 9 (macrophage enriched) versus spatial niche 1 (right).

As an example, we used the CLQ method to stratify cells in a human lung cancer MERFISH sample by attraction or avoidance to each cell type (Fig. 5C). We investigated how proximity to T cells modulates inflammatory and cell proliferation pathways (Fig. 5D). We noticed upregulation of NFKb pathway genes and downregulation of Trail and TNFa genes in epithelial cells, upregulation of EGFR genes and downregulation of TNFa genes in myeloid cells, and downregulation of p54 genes in endothelial cells, when split by proximity to T cells. Furthermore, the integration of the CLQ with omics data allowed us to identify genes that are differentially expressed in the proximal presence or absence of another cell type. As an example, we assessed how gene expression differs between T cells that are classified as “attractive” to or “avoidant” of epithelial cells (as determined by CLQ analysis).

The CLQ method is limited to pairwise relations and does not take into account higher-order interactions with additional cell types within potential functional communities composed of multiple cell types. We acknowledge that spatial patterns exist beyond pairwise spatial correlations, and as such, we introduce the next analysis module, SPEX niche analysis. This module identifies cell communities by determining the neighborhood composition for each cell in the image at a given radius and then clustering all the different neighborhoods to identify cell communities.

In the lung tumor MERFISH sample, the resulting analysis demonstrated the presence of cell-type communities that define regions of interest in the image with potentially different biology. This allowed us to extract further information than the mere projection of the cell types identified by transcriptomics back into the image (Fig. 5B). SPEX communities, when highlighted in the tissue slide, allow for the identification of potential structures (Fig. 5E). For example, SPEX communities bring out aggregates of B cells surrounded by a layer comprising a combination of B cells, epithelial cells, and T cells potentially defining TLS (Fig. 5F). Beyond the spatial information, the clusters themselves can be characterized using pathway analysis. Pathways will be present as a function of the cell types involved in the community and their contextual transcriptomic state. In the present case, TGF-$\beta$ has larger activity in communities containing more fibroblasts (Fig. 5G).

The SPEX community analysis module can also be leveraged to look at specific cell types in the context of a particular microenvironment. More concretely, differential gene expression analyses for the same cell type across clusters can further identify context-dependent expression patterns (Fig. 5H). We highlight how the T cells tend to express more CD8A in SPEX community 8 (B-cell associated) compared to 10, potentially indicating a differentiation toward more cytotoxic T cells in that community. Tumor cells also show some interesting patterns; when associated with stroma, they express more HLA and CYR61, involved in angiogenesis. Overall, our community module generates hypotheses about regions of the tissue where potential contextual phenotypes are observed and guides the further exploration of the data.

## Discussion

Cells in complex multicellular organisms are hierarchically arranged into tissues that are in turn organized into organs, and so on. At each level of organization, structure is closely related to function. In tissues, both during and after development, structure is governed by different gene expression programs that in turn depend on the resulting architecture of the cellular microenvironment experienced by the individual cells composing the tissue. This suggests that if we can measure gene and protein expression of individual cells in a given tissue and efficiently incorporate spatial cell arrangement information into these measurements, we would be able to discern the molecular basis of tissue function and dynamics.

Currently, the fast pace of the development of spatial transcriptomics and proteomics [22] is bringing us closer to fulfill this scientific program. Nevertheless, the data generated by these methodologies are very rich in information, and their successful analysis and interpretation depend on the existence of computational pipelines. Most of the solutions provided by the scientific community are libraries for use by computational biologists, exposing the need for a graphical user interface application to enable the direct interaction with the data from other domain experts such as pathologists or biomedical scientists. Although there are commercially licensed platforms that offer more accessible graphical interfaces, their application is restricted due to high costs, license limitations, and lack of generalizability across different modalities or platforms. This highlights the dual need for an open-source solution and a modular, easily adaptable tool.

In response to this requirement, we presented in this work SPEX, a powerful toolkit that offers a graphical user interface for analysis of spatially resolved omics data. The unique modular design of SPEX provides flexibility, enabling users to create bespoke data analysis pipelines. Users can directly interact with data through the graphical interface, significantly easing the process of data analysis and interpretation by eliminating the need for low-level coding.

The SPEX platform demonstrates flexibility in analyzing diverse biological data types, illustrating both its breadth of functionality and its modality-agnostic adaptability. For IMC data, we leveraged methods in SPEX to establish an end-to-end workflow to systematically characterize the spatial organization and molecular features of the highly structured microenvironment of human tonsil tissue. Additional application extended to analyzing regions of interest in a human PDAC dataset, wherein we characterized TLS positioned within a heterogeneous tumor microenvironment.

Our exploration continued in other high-dimensional imaging modalities, where we leveraged the CLQ method to quantify local cell–cell interaction in a TNBC MIBI dataset. The outcomes were not only consistent with previously reported results by orthogonal methods but also provided novel insights into the relationship between spatial proximity and function of macrophage populations present in varying microenvironments defined by tumor-immune spatial colocalization. While these applications highlight CLQ’s biological utility, practical adoption on large spatial-omics datasets also requires computational efficiency. To assess the computational performance of CLQ, we benchmarked its execution time and memory usage alongside other commonly used cell–cell interaction scoring methods. As summarized in Supplementary Figure S1A, B, SPEX-CLQ consistently demonstrated lower memory usage and faster processing times as the number of cells increased, compared to alternative approaches—with the exception of Squidpy. Both CLQ and the comparable method implemented in Squidpy leverage Numba, a just-in-time (JIT) compiler for Python that translates a subset of Python and NumPy code into fast machine code at runtime. While Squidpy exhibited greater efficiency in terms of RAM and runtime, our CLQ approach offers a unique advantage by generating a localized CLQ map, enabling spatial visualization of where attraction or avoidance patterns occur within the tissue. This added layer of spatial interpretability provides biologically meaningful insights that are not readily captured by global summary statistics alone.

Finally, we explored the ability of SPEX methods to tackle deeper data analysis tasks by analyzing a non–small cell lung cancer (NSCLC) spatial transcriptomics dataset. We integrated gene expression analysis with spatial information by leveraging the SPEX niche analysis module. We identified microenvironment domains within the tumor tissue where particular transcriptional programs are more active. Across all these use cases, SPEX showed its potential to discover and characterize informative biological programs from spatially resolved omics data.

We are aware that there are still many open questions that warrant the development of analysis modules. Our analyses indicate that “ambient RNA” diffusion in the slide is a problem of MERFISH samples that goes beyond the need to improve the cell segmentation step. This is an example of many possibilities for future development, and therefore, we hope that the scientific community will embrace this software tool and expand upon its modules.

While we foresee immediate utility of the current SPEX version in the emerging field of spatially resolved omics, we recognize the need to enhance its capabilities for the imaging processing of whole-slide imaging data and spatial transcriptomics modalities. In the forthcoming years, the field of spatially resolved omics is expected to progress toward generating multimodal readouts, encompassing transcriptomics, proteomics, T-Cell Receptor (TCR) and B-Cell Receptor (BCR) repertoire, epigenomics, and metabolomics, all from the same tissue slide [22, 23]. Therefore, we present SPEX, not merely as a static tool, but as an adaptable and extensible open-source solution, directly catering to the diverse and rapidly evolving needs of the spatial omics research community. SPEX is available at [24] and has been registered in the bio.tools database under the identifier *biotools:spex*.

## Methods

### Data overview

To demonstrate SPEX capabilities, we leverage both newly generated and published datasets. The published datasets include raw MIBI data for a cohort of 41 TNBC patients that was downloaded from [3].

Control Tonsil Tissue Antibody staining for imaging mass cytometry was performed on 4$\mathrm{\mu }$m-thick, FFPE tissue sections mounted on SuperFrost Plus (Erie Scientific) glass slides. Antibodies to cytokeratin (cat. ab80826), CD103 (cat. ab221210), and PD-L1 (cat. ab226766) were purchased as purified, carrier-free formulations from Abcam and, using the Maxpar X8 Antibody Labeling Kit (Fluidigm), conjugated to 176-Yb (cat. 201167A), 153-Eu (cat. 201153A), and 150-Nd (cat. 201150A), respectively, per the manufacturer’s protocol. Remaining antibodies were purchased as conjugated from Fluidigm. To stain, tissue was first deparaffinized and rehydrated. Next, pH 9.0 antigen retrieval was used, followed by incubation in Superblock at room temperature for 30 minutes. Samples were incubated with a staining panel comprising 16 antibodies (see Supplementary Table S3) overnight at 4°C in a humidity chamber. Samples were washed 4 times in 0.1% TritonX/phosphate-buffered saline (PBS) for 4 minutes each, followed by two 4-minute washes in PBS at room temperature. Samples were then incubated with Ir-Intercalator dye for 30 minutes at room temperature, washed in water for 5 minutes, and air-dried. ROIs measuring 1,800 × 2,000 $\mathrm{\mu }$m each were selected from the slide and acquired at 200 Hz. Data were exported as MCD files and visualized.

For PDAC imaging mass cytometry samples, slides were first baked at 70°C for a minimum of 30 minutes to remove all visible wax. They were then subjected to a deparaffinization and rehydration series using xylenes, alcohol, and water in an autostainer. Antigen retrieval was subsequently achieved using the EZ-Retriever System (BioGenex), and the slides were cooled to about 74°C in EZ-AR1 solution. After a 10-minute wash in Maxpar Water, the samples on the slide were encircled with a PAP pen and then blocked with a freshly prepared 3% BSA in Maxpar PBS solution. For antibody staining, an overnight MCA cocktail specific for the assay was prepared. The slides were incubated overnight with this mixture at 4°C and given a secondary antibody for 30 minutes, following a rinse in Maxpar PBS. Counterstaining involved washing slides with 0.2% Triton X-100 in Maxpar PBS, followed by rinses in Maxpar PBS. Slides were then incubated in a diluted Intercalator-Ir solution. Finally, slides were washed in Maxpar Water and air-dried overnight at room temperature.

MERFISH lung tumor samples from patients with human lung cancer were snap-frozen and preserved in optimal cutting temperature (OCT) compound and cut into 10-$\mathrm{\mu }$m-thick slices on a cryostat at −20°C and placed on MERSCOPE Slide (Vizgen 20400001). The tissue slices were fixed with 4% paraformaldehyde in 1× PBS for 15 minutes, washed 3 times with 5 mL 1× PBS, and incubated with 70% ethanol at 4°C overnight for tissue permeabilization. Samples were then stained for cell boundary using Vizgen’s Cell Boundary Kit (10400009) and later hybridized with a custom-designed MERSCOPE Gene Panel Mix consisting of 484 genes that assess the different cell types (tumor, stroma, immune cells, etc.) and key cell signaling and activity marker (Vizgen 20300008) in a 37°C incubator for 36 to 48 hours. Following incubation, the tissues were washed with 5 mL formamide wash buffer at 47°C for 30 minutes twice and embedded into a hydrogel using the Gel Embedding Premix (Vizgen 20300004), ammonium persulfate (Sigma, 09913-100G), and TEMED (N,N,N′,N′-tetramethylethylenediamine) (Sigma, T7024-25ML) from the MERSCOPE Sample Prep Kit (10400012). After the gel mix solution solidified, the samples were cleared with clearing solution consisting of 50 μL Protease K (NEB, P8107S) and 5 mL Clearing Premix (Vizgen 20300003) at 37°C overnight. After removing clearing solution, the sample was stained with DAPI and Poly T Reagent (Vizgen 20300021) for 15 minutes at room temperature, washed for 10 minutes with 5 mL Formamide Wash Buffer, and then imaged on the MERSCOPE system (Vizgen 10000001). A fully detailed, step-by-step instruction on the MERFISH sample prep full protocol is available at [25]. Full instrumentation protocol is available at [26].

### SPEX modules

The SPEX graphical user interface provides essential components to facilitate comprehensive analysis of spatial omics images and datasets. SPEX supports end-to-end modules for spatial proteomics, covering both image processing and downstream single-cell spatial analysis. Since the most single-cell spatial transcriptomics platforms output cell-by-transcript files with spatial coordinates, SPEX will also directly ingest a cell-by-transcript Anndata file (.h5ad) for downstream clustering and/or single-cell spatial analysis. For nonimaging, sequencing-based spatial transcriptomics platforms such as 10x Genomics Visium HD, users can leverage existing tools like bin2cell to transform bin-level data into pseudo single-cell resolution. The transformed data, structured as a cell-by-gene matrix with spatial coordinates, can then be converted into an AnnData object and processed by SPEX for downstream analyses such as clustering, spatial feature extraction, and colocation analysis.

#### Image processing

Image processing is executed in a 5-step sequence with the ultimate goal of generating a cell-by-expression matrix. These steps include image loading, image preprocessing, single-cell segmentation, postprocessing, and feature extraction. Each step contains a selection of modules, which can be linked together to address the particularities of a given image set.

#### Image loading

The image loading step contains a single module that supports OME-TIFF or OME-ZARR. This multidimensional open format provides a well-structured metadata header that can accommodate a wide range of image and acquisition system information. A number of open-source conversion pipelines are available in the event proprietary microscopy formats need to be converted to OMETIFF or OME-ZARR. Within SPEX, the AICSimageio Python library [27] is used to read these open formats as in-memory multichannel Numpy arrays. SPEX also supports the loading of H5AD files, which are a data format for AnnData objects.

This allows SPEX users to utilize H5AD files as data sources. After loading, these data can be processed and analyzed in the SPEX pipeline for those modules that provide this capability.

The preprocessing step includes optional modules to denoise images and/or enhance pixel information to facilitate single-cell segmentation. These modules include global background correction, median filter denoising, and nonlocal means (NLM) denoising. All modules were developed using the Scikit-image Python library. To accommodate the potentially large channel dimension of images, channels are processed in parallel with use of the apply *parallel()* function in SCIKIT image, a wrapper for the DASK map *blocks()* function. This routine allows application of the denoising functions on chunked arrays, which are efficiently distributed across computing cores.

In the global background correction module, background signal, as captured in 1 channel, can be subtracted from all other channels. For example, signal in an autofluorescent or a detector noise channel can be subtracted from channels that house specific molecular markers. Within the defined background channel, OTSU thresholding [28] is applied to create a positive pixel binary mask. This pixel mask is then intensity-scaled by a user-defined correction factor. This correction mask is then subtracted from the other channels.

Median denoising is a common image-processing technique that can preserve edge information while suppressing unstructured noise. For any channel, the filter replaces a pixel’s value with the median of its local neighborhood. The size of the local neighborhood is defined by the filter kernel size, an argument of the function.

NLM denoising provides a slightly more sophisticated method where a pixel’s value is replaced by a mean that is sampled from other regions of the image. These sampled regions are only utilized if their mean is similar to the target region. This technique can preserve local texture information.

Single-cell segmentation SPEX includes several algorithms for single-cell segmentation. This includes traditional watershed cell segmentation in addition to a collection of pretrained deep learning models, which include Stardist [29] and Cellpose [18]. These algorithms will operate on the channel or combination of channels containing cell nucleus information. In instances where the input data differ in resolution to the training dataset for the respective models, the user can up-sample or down-sample the image pixels. All segmentation modules will return an instance segmentation label image where each cell is assigned a unique integer value.

The optional postprocessing modules aim to modify the single-cell segmentation labels. Tissue-based imaging is often prone to include technical artifacts. This could be tissue tears, folds, or debris. These artifacts may generate false-positive cell segmentation labels. Therefore, we include rule-based functions to exclude segmented labels based on morphological features.

In some rare instances, the pretrained deep learning segmentation may include false-negative regions. This is likely a result of the model not having representation of this cell type in the training data. In these cases, one can utilize the optional 'rescue_cells' algorithm. First, prototypical cell size and intensity are calculated adaptively from the currently segmented cell objects. These parameters are then used as input for classical watershed segmentation of the image. If a label from the watershed segmentation does not overlap with that of the deep learning approach, then it is merged into the final instance segmentation label image.

The user then has an option to expand the boundaries of the cell. This may be desired since some of the cell segmentation modules utilize only nuclear information. To ensure cytoplasmic compartments are included in feature extraction, boundaries should be dilated.

SPEX utilizes the scikit image regionprops function to extract single-cell features. Currently, these features include mean intensity for each channel, extracted for each cell. The output will be a cell-by-expression matrix in Anndata format, which serves as input for downstream cell-type clustering.

#### Proteomics data clustering

An Anndata object containing the cell-by-expression matrix obtained from the upstream image-processing modules can be clustered into groups of cells that share the same properties. The SPEX proteomics clustering module leverages PhenoGraph for cell typing [14]. Within this module, users will define a transformation and scaling method that is most suitable for their data. Arcsin and Log are available as transformation options. Winsorizing and *z*-scoring are available as scaling options.

#### Transcriptomics data clustering

Cells described by a cell-by-expression matrix obtained from the set of image-processing or spatial transcriptomics ingestion modules can be clustered by gene expression. Currently, we provide a standard method to cluster the cells using the popular scanpy package. Cells with fewer than 20 total UMI counts were excluded from analysis. The cell-by-expression matrix is first normalized to the median total counts and log-transformed. The matrix is then whitened, and principal component analysis (PCA) is performed (the optimal number of components is determined from the singular value spectrum). A nearest-neighbor graph ($K=\sqrt{\text{number of cells}}$) is constructed on the PCA matrix, and the graph is clustered using the Leiden algorithm with the default parameters.

#### Cell-type annotation

After unsupervised clustering is executed, clusters can be renamed and grouped to facilitate more meaningful downstream spatial analysis within the SPEX application. To facilitate annotation of clusters to biologically relevant cell types, SPEX offers a manual classification module where users can easily rename cluster identifiers. The fully integrated Vitessce spatial omics visualization dashboard aids in this process by providing a means to interpret the expression profile of the cluster through heatmaps. Vitessce spatial plots can also aid in proper cluster classification as certain cell types may have specific localization patterns. One can also export the SPEX clustering results in Anndata format and leverage a wide variety of cell-type annotation tools available in Python or R. For analysis of the NSCLC MERFISH data, we utilized the Pegasus [30] Python package for cell typing.

#### Spatial analytics module

##### Cell–cell co-occurrence at the cell population level

To systematically assess the nonrandom co-occurrence/avoidance of identified cell types at the cell population level, we used a permutation test to compare the number of interactions between all of the cell types in a given image (or in a given user-defined region) to that of synthetic matched controls generated under the null hypothesis containing randomized cell phenotypes. The neighborhood radius (e.g., 20 $\mathrm{\mu }$m) was chosen empirically to restrict cell–cell co-occurrence assessment to only those cells that are close enough to potentially establish physical contact between their membranes. This approach, similar to the one described in the histoCAT paper [31], allows one to determine the significance of cell–cell co-occurrence between 2 cell types.

##### CLQ: cell–cell co-occurrence at enhanced resolution

The CLQ, defined as a ratio of ratios, is a widely used geographic index [32]. In the context of tissue images, it is used to measure the local density to the global density of a specific cell type [33]. Specifically, the local density is calculated as the proportion of cell type B in the neighborhood constructed by a certain radius, centered around cell type A. The global density is the proportion of cell type B in the entire tissue image. In general, if the density of cell type B within the neighborhood of A is more than the global density of B, the CLQ will be $>1$. If the neighborhood of cell type A contains many other cell types other than B, the CLQ will be $<1$. The value 1 means there is no spatial relationships between the 2 cell types.

The CLQ can be measured globally for a particular cell type A, $\text{CLQ}_{A\rightarrow B}$, or locally for each cell in the slide classified as that particular cell type, which we denote as $\text{LCLQ}_{A_i\rightarrow B}$. The CLQ is calculated through the following expressions:


(1) \begin{eqnarray*}
\text{LCLQ}_{A_i\rightarrow B}&=\frac{N_{A_i\rightarrow B}}{N_b\left(N-1\right)}
\end{eqnarray*}

(2) \begin{eqnarray*}
N_{A_i\rightarrow B}&= \sum \frac{w_{ij}\delta _{ij}}{\sum w_{ij}}
\end{eqnarray*}

(3) \begin{eqnarray*}
\text{CLQ}_{A\rightarrow B}&= \frac{\sum _{i=1}^{N_A} \text{LCLQ}_{A_i\rightarrow B}}{N_{A}}
\end{eqnarray*}

where $N$ is the total number of cells in the image, $\delta _{ij}$ is the Kroenecker delta indicating whether cell $j$ is a type B cell, and $w_{ij}$ is $1/N$ for the nonweighted version and Gaussian distance decay kernel for the weighted version, which is common in geostatistics application [34]. The global CLQ will then be the average of the local CLQ.

To evaluate the statistical significance of spatial associations identified by CLQ, we implemented a permutation-based hypothesis testing framework in our SPEX software, following an approach similar to that used in histoCAT for assessing cell–cell interactions in multiplex imaging data [31]. Under the null hypothesis, cell-type labels are assumed to be randomly distributed in space, implying no spatial association between cell types. To preserve tissue architecture, the spatial coordinates of all cells were kept fixed while the cell-type labels were randomly permuted across cells. For each test, 999 random permutations were performed, and the observed CLQ value was included in the null distribution, yielding a total of 1,000 values. Empirical *P* values were then calculated. The *P* value for spatial attraction was defined as


(4) \begin{eqnarray*}
\text{P}_{attractive}&=\frac{counts(CLQ_{perm}\ge CLQ_{obs})+1}{999+1}
\end{eqnarray*}

and for spatial avoidant as 


(5) \begin{eqnarray*}
\text{P}_{avoidant}&=\frac{counts(CLQ_{perm}\le CLQ_{obs})+1}{999+1}
\end{eqnarray*}

where $CLQ_{perm}$ denotes the CLQ value calculated on each permuted sample, and $CLQ_{obs}$ denotes the CLQ value calculated on the original sample. The “+1” correction ensures that the observed statistic is included in its null distribution and avoids zero *P* values, consistent with best practices in nonparametric testing. Associations with $P_{\rm {attractive}} < 0.05$ were considered significantly attractive, and those with $P_{\rm {avoidant}} < 0.05$ were considered significantly avoidant. Other associations were classified as nonsignificant.

##### Spatially informed transcriptomics using CLQ scores

For each cell type, we estimated a CLQ score that describes the spatial relationship between cells (or globally as clusters), classifying them as “avoidant” or “attractive” spatial relationships. The cell-level CLQ classifications can be further investigated to identify differences in gene expression associated with, for example, “avoidant” behavior in clusters of interest. Gene expression differences were estimated using the scanpy (version 1.9.4) [35] using default parameters. Hypothesis-driven testing of cell clusters and cell–cell spatial relationships for differences in gene expression can be performed flexibly using SPEX.

##### Spatially informed transcriptomics using SPEX clusters

For each cell in the dataset, neighborhood compositions were determined by collecting the counts of cells belonging to the different clusters included within a circle of radius epsilon centered on each cell (a parameter that can be adjusted by the user to assess the locality of expected cell interactions). SPEX communities then were identified via clustering of the matrix of the neighborhood compositions using PhenoGraph.

MERFISH single-cell data were processed using the squidpy package [10] (version 1.3.0) and then fed into SPEX for spatial analyses. For each cluster identified, CLQ was calculated and neighborhood compositions were determined for each cell. SPEX communities were identified via clustering neighborhood compositions using the Leiden algorithm as implemented in scanpy (version 1.9.4). These communities were then analyzed using decoupleR [36] (version 1.4.0) to infer cell–cell interactions and pathways specific for said communities. SPEX communities were also inspected for pathway activities from a collection of well-understood transcriptional pathways using progeny [37] (version 1.12.0).

#### Custom analysis modules

To accommodate the growing collection of spatial omics methods emerging in the field, SPEX supports the integration of custom plug-in modules. Users can develop their own Python-based analytical modules and integrate them into the SPEX platform for execution within a pipeline. The primary components of a custom module are (i) an *app.py* file, which contains the core function (reading data, processing data, and returning results) and (ii) a json file, which defines the required parameters, pipeline execution order, dependencies, and other global settings. Detailed documentation, along with templates, can be found on our GitHub repository [24]. This guide outlines how to define module inputs and outputs to ensure compatibility with the existing SPEX architecture.

### SPEX back-end

SPEX’s architecture design is mainly driven by the requirements to the system performance, availability, and scalability. In particular, we aimed for an architecture supporting multiuser access to computing resources while ensuring and guaranteeing the execution of the resource-heavy tasks set by the user in a reasonable time frame. As an additional requirement, we considered the ease of system extension and maintenance. All these requirements are fulfilled by the microservice architecture that is inherently flexible as it is based on the idea of interoperability of small, loosely coupled, and easily modifiable modules (microservices). A microservice is a small program that performs a clearly assigned task. Each microservice is hosted in a Docker container and can run individually or as a group via docker-compose. SPEX microservices communicate through the data bus instrumented by Redis. Redis manages message transfer between the services, ensuring message order and guaranteed delivery, even if temporary system outages occur. Message distribution and load balancing mechanisms allow one to easily increase the number of the microservices’ instances and spread them across different servers, enabling straightforward scaling and performance-boosting possibilities.

To ensure SPEX can be deployed on a variety of environments and provide recommendations for resource specifications, we benchmarked execution of analysis pipelines for the reported datasets across 4 different compute environments (Supplementary Fig. S1C, D). This included, Intel (CPU: Intel i5-13500, 14 cores/20 threads, 800–4,800 MHz, memory: 62 GB RAM), AMD (CPU: AMD64 Family 25 Model 117, 3.8 GHz, 1 processor, virtualization enabled, memory: 62 GB RAM, virtual memory: 97 GB total), AWS (CPU: AMD EPYC 7571, 8 cores/16 threads, base 3.2 GHz, memory: 62 GB RAM), and a notebook computer (CPU: AMD Ryzen 7 5800H, 8 cores/16 threads, base 3.2 GHz, memory: 45 GB RAM). Both processing time and memory usage for specific modules are reported. Single-cell segmentation and clustering represented the most memory intensive steps of the pipeline. This time-scaled with dataset size and complexity. Segmentation is inherently memory-intensive because it involves processing large, multidimensional image data, often requiring the simultaneous loading of multiple image tiles or channels into memory for normalization and inference. Clustering, on the other hand, operates on large, high-dimensional single-cell feature matrices. Memory usage grows rapidly as the number of cells and features increases, since many clustering algorithms require storage of distance matrices or neighborhood graphs.

The integration of SPEX and the OMERO image management applications is implemented via a separate microservice. This service is responsible for creating OMERO sessions operating on 2 different layers.

The first layer, known as Python Blitz Gateway, is primarily used for direct access to images. It enables batch downloading of images in chunks (through ms-image-loader) and also permits the retrieval of image metadata not available through another layer of OMERO. The second layer, named OMERO WEB API, facilitates user interaction with images and projects. More specifically, it enables the request of metadata pertaining to user projects, accessible images, and compressed image fragments. Both layers are integral to SPEX since neither possesses all the necessary OMERO functions. Thus, to accomplish specific tasks, both layers must be used. They are created and managed by the microservice named ms-omero-session and subsequently stored as objects in REDIS. From there, they can be accessed by other microservices and the back-end system.

A proxy connection has been implemented to enable interactions with the OMERO WEB API. This mechanism proxies all requests to the OMERO WEB through our back-end, utilizing a web session created on the OMERO WEB API layer by the ms-omero-session microservice. That session is then stored as an object in REDIS. This approach allows for requests to be overridden and reused, with the ms-omero-session microservice ensuring that the session remains active and accessible when necessary.

During the initiation of image-processing scripts from the pipeline, the primary layer invoked is the Python Blitz Gateway. This layer is instantiated by the microservice referred to as ms-omero-session and is then stored as an object within REDIS.The ms-job-manager microservice necessitates a Python Blitz Gateway session. This session, created and stored in REDIS by the ms-omero-session microservice at the time of user login, is persistently monitored and refreshed to ensure its availability for reuse when required.

During the execution of pipeline blocks within the microservice ms-job-manager, the computational results are converted into the anndata-zarr format, which is compatible with Vitessce [12]. These converted data are then stored in a network storage system. This storage serves as a data source for subsequent use on the client’s front-end. The combination of Vitessce with data collection for creating interconnected datasets (multiple zarr files) into a unified dashboard enriches progressively with data from each pipeline step. This provides an excellent opportunity for a comprehensive overview of the entire research subject without the need to remember the full context of the experiment.

In SPEX, the data visualization system Vitessce is utilized, shifting data visualization workload from server to client side. This approach leverages cloud access capabilities within Vitessce. A key feature is the use of the H5AD to Zarr format conversion, which is compatible with Vitessce. This conversion allows for efficient data handling, as it enables users to load only the data chunks currently in use, rather than the entire dataset. This not only reduces the load on the user’s device but also allows for repeated reuse of data obtained from pipeline outputs, with added filters and detail levels.

Furthermore, Vitessce is built upon WebGL2, which facilitates the transfer of data visualization processing to the graphics processing unit. Currently, Vitessce configuration files are back-end generated, offering simpler data visualization compared to seaborn and plotly. This requires initial technical expertise but ultimately reduces time in system support and development.

## Ethics, Consent, and Permissions

Tissue samples were supplied by the following commercial biobanks: IMC Tonsil (Bio-Options, Inc.), IMC PDAC (Indivumed), and MERFISH NSCLC (BioreclamationIVT). Each supplier received institutional review board approval, appropriate informed consent of all subjects, contributing biological materials, and all other authorizations, consents, or permissions as necessary for the transfer and use of the biological materials for research at Genentech. Patients/human donors were not compensated for their donation of tissues.

## Availability of Supporting Source Code and Requirements

Project name: SPEX (Spatial Expression Explorer)

Project homepage: https://github.com/Genentech/SPEX

Operating system(s): Linux, Windows

Programming language: Python, Javascript

Other requirements: Docker, Git, Git LFS, modern web browser (Chrome or Firefox), 64-bit x86 system

License: Apache License, Version 2.0

RRID: SCR_026970

bio.tools ID: biotools:spex

## Additional Files

**Supplementary Table S1**. Comparison of SPEX with existing spatial omics tools.

**Supplementary Table S2**. SPEX analysis modules.

**Supplementary Table S3**. Antibodies, conjugates, clones, and working concentrations used in tonsil multiplex staining.

**Supplementary Fig. S1**. Pipeline and method benchmarking. (A) Memory and (B) time of CLQ algorithm against other cell–cell interaction methods with increasing cell number. CLQ benchmarking utilized 60 GB RAM and 8 cores. (C) Memory usage in megabytes and (D) processing time in seconds for each analytical module (x-axis) utilized in the SPEX analysis pipeline for IMC Tonsil, IMC PDAC, MIBI TNBC, and MERFISH NSCLC, respectively. Each datapoint corresponds to a single input and is colored by the compute environment utilized. The IMC PDAC dataset consisted of 4 images, and MIBI TNBC consisted of 41 images processed as a batch in the SPEX application. IMC Tonsil and MERFISH NSCLC had 1 sample each. Pipeline benchmarking utilized the following systems: Intel (CPU: Intel i5-13500, 14 cores/20 threads, 800–4,800 MHz, memory: 62 GB RAM), AMD (CPU: AMD64 Family 25 Model 117,  3.8 GHz, 1 processor, virtualization enabled, memory: 62 GB RAM, virtual memory: 97 GB total), AWS (CPU: AMD EPYC 7571, 8 cores/16 threads, base  3.2 GHz, memory: 62 GB RAM), and a notebook computer (CPU: AMD Ryzen 7 5800H, 8 cores/16 threads, base 3.2 GHz, memory: 45 GB RAM).

giaf090_Supplemental_Files

giaf090_Authors_Response_To_Reviewer_Comments_Original_Submission

giaf090_GIGA-D-25-00022_Original_Submission

giaf090_GIGA-D-25-00022_Revision_1

giaf090_Reviewer_1_Report_Original_Submission Ka Yee Yeung, Ph.D. -- 3/9/2025

giaf090_Reviewer_2_Report_Original_Submission Qianqian Song -- 3/11/2025

giaf090_Reviewer_2_Report_Revision_1 Qianqian Song -- 6/1/2025

giaf090_Reviewer_3_Report_Original_Submission Hongyoon Choi -- 3/11/2025

## Abbreviations

BCR: BCR B-cell receptor; CLQ: Colocation Quotient; FDC: follicular dendritic cells; FFPE: formalin-fixed, paraffin-embedded; IMC: imaging mass cytometry; JIT: just-in-time; MIBI: Multiplex Ion Beam Imaging; NLM: nonlocal means; NSCLC: non–small cell lung cancer; OCT: optimal cutting temperature; PBS: phosphate-buffered saline; PCA: principal component analysis; PDAC: pancreatic ductal adenocarcinoma; ROI: region of interest; SPEX: Spatial Expression Explorer; TCR: cell receptor; TLS: tertiary lymphoid structure; TME: Tumor Microenvironment; TNBC: triple-negative breast cancer.

## Competing Interests

X.L., X.P., D.O., and R.J. are coinventors on a provisional patent application filed by Genentech/Roche relating to this manuscript.

## Author Contributions

X.L., X.P., C.F. T.R., D.O., and R.J. wrote the manuscript with input from all authors. X.L., X.P., D.O., and R.J. conceived the SPEX platform. D.O. and R.J. supervised the project (D.O. from 2020 to 2022 and R.J. and J.G. from 2022 to present upon D.O.’s departure from Genentech). J.G. also provided the software deployment strategy. R.J. designed the UI with input from authors and end users, developed image-processing methods, and executed image processing on spatial proteomics datasets. X.L., X.P., C.F., and D.O. developed spatial analysis methods. X.P., D.H., and C.F. developed code for spatial transcriptomics analysis and executed analysis of spatial transcriptomics datasets. C.C. developed and tested single-cell data structure schema. T.R. analyzed and interpreted MIBI data. S.R., P.C., and J.Z. executed method development of IMC PDAC data. D.O. performed clustering analysis of IMC Tonsil data. F.P. analyzed and interpreted IMC Tonsil spatial data. Z.S., M.N., and X.Y. generated Lung MERFISH data and executed primary analysis. L.M. provided interpretation and validation of spatial methods. J.C. and J.S. provided infrastructure engineering and OMERO integration work. H.C.B. provided software development supervision. A.Z. and E.T. executed front-end and back-end software development. A.P. executed front-end and back-end software development in addition to software testing. Both X.L. and X.P. contributed equally and have the right to list their names first in their CVs. All authors read and approved the final manuscript.

## Data Availability

Datasets used and/or analyzed in this work are made available at [38]. The software code is available from the GitHub repository [24, 39–46]. Snapshots of the code are available in Software Heritage [47–55]. All additional datasets used in this research can be found in the *GigaScience* database, GigaDB [56].
