# Supplementary material for: SPEX: A modular end-to-end platform for high-plex tissue spatial omics analysis
Source: Gigascience. 2025 Aug 29;14:giaf090. doi: 10.1093/gigascience/giaf090 (PMC12395962; doi:10.1093/gigascience/giaf090)
Supplement: giaf090_Supplemental_Files [file giaf090_supplemental_files.zip › Table S1-3.pdf]

Table S1. Comparison of SPEX with existing spatial omics tools.

Tool/Package	Squidpy	Giotto	MCmicro	Aquilla	EZSingleCell	SPEX
GUI or code-based execution	Code-based: Python library	Code-based: R	Code-based: Nextflow	Code-free: Web application	Code-free: Web application	Code-free: Web application
Target Audience & Prerequisites	Python experience required	R coding experience required	Coding experience required	No coding experience needed	No coding experience needed	No coding experience needed
Data types supported	multi-channel TIFF, OME-TIFF, ZARR, Anndata	S4 object system in R	OMETIFF (Image), CSV	PostgreSQL (ROI annotation, cell/spot coordination, gene expression)	CSV, TSV, 10x Cell Ranger/Space Ranger/Cell Ranger-ATAC output (.H5)	OMETIFF (Image), OMEZARR (Image), Anndata (single-cell object)
Interactive Image (pixels) visualization	Napari-interactive, multi-dimensional image viewer in Python	Interactive viewer on the user's local computer	MINERVA downstream integration	N/A	Low-resolution Space Ranger output	Integrated Image Viewer
Image Preprocessing	Image cropping, subsetting, filtering, normalization	N/A	Image Alignment	N/A	N/A	Median Filter, Background Subtraction
Single-cell segmentation	Watershed, Cellpose, StarDist, Custom (skimage/OpenCV)	Mesmer	uMINST	N/A	N/A	Cellpose, Stardist, Watershed
Cell Type Clustering	Leiden, Louvain, GMM, SingleR, scVI	N/A	N/A	N/A	Seurat, CellID, Celltypist	Phenograph
Spatial analysis	Spatial graphs, Moran's I, Geary's C, co-occurrence, neighborhood enrichment, spatial variability, ligand-receptor analysis	Spatial enrichment, deconvolution, coherent expression, neighborhood analysis	SCIMAP platform	Cell-cell interaction, neighborhood analysis, spatial entropy, spatial variable genes	GraphST	CLQ Cell-Cell Interaction, Niche/Neighborhood Analysis
Image Data management	ImageContainer (wrapper of xarray.Dataset), Lazy loading, Metadata storage	N/A	N/A	N/A	N/A	OMERO Integration
Data Visualization	Matplotlib, Seaborn, Scanpy's plotting functions	R data visualization libraries	MINERVA	Web-based	R Shiny Web Application	Vitessce Integration

Table S2. SPEX Analysis Modules

**Category**	**Module**
2I/O	Load Tiff<br>Load Image
Image Preprocessing	Median Denoising<br>NLM Denoising<br>Background Subtraction
Image Segmentation	Watershed<br>Stardist<br>Cellpose
Segmentation Post-processing	Remove Small Objects<br>Remove Large Objects<br>Simulate Cell<br>Rescue Cells
Clustering (Proteomics)	Feature Extraction<br>Phenograph
Clustering (Transcriptomics)	Preprocessing<br>Scanpy Clustering
Transcriptomics Secondary Analysis	Differential Expression Analysis<br>Pathway Analysis
Spatial Analysis	Colocation Quotient (CLQ)<br>Niche Analysis

Table S3. Antibodies, conjugates, clones and working concentrations used in tonsil multiplex staining

Antibody	Conjugate	Clone	Concentration (μg/mL)
CD20	161-Dy	H1	3
CD45	152-Sm	D9M8I	7
CD68	159-Tb	KP1	0.1
CD8	162-Dy	CD8/144B	2
Collagen type I	169-Tm	Polyclonal	1
Cytokeratin	176-Yb	AE1/AE3	0.8
Histone H3	171-Yb	D1H2	0.5
Ki67	168-Er	B56	3
BCL2	146-Nd	EPR17509	7
CD25	175-Lu	EPR6452	5
CD31	151-Eu	EPR3094	5
CD336/Tim3	154-Sm	D5D5R	7
PD1	165-Ho	EPR4877	5
PDL1	150-Nd	73-10	10
