# Supplementary figures and images for: SPEX: A modular end-to-end platform for high-plex tissue spatial omics analysis
Source: Gigascience. 2025 Aug 29;14:giaf090. doi: 10.1093/gigascience/giaf090 (PMC12395962; doi:10.1093/gigascience/giaf090)

A

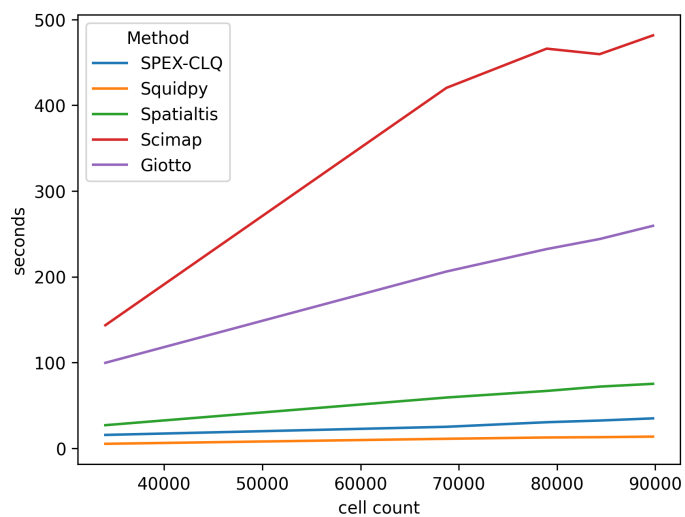

B

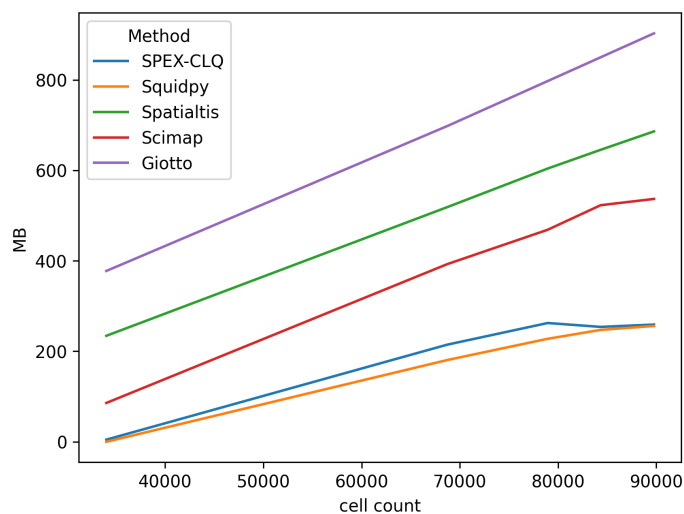

C

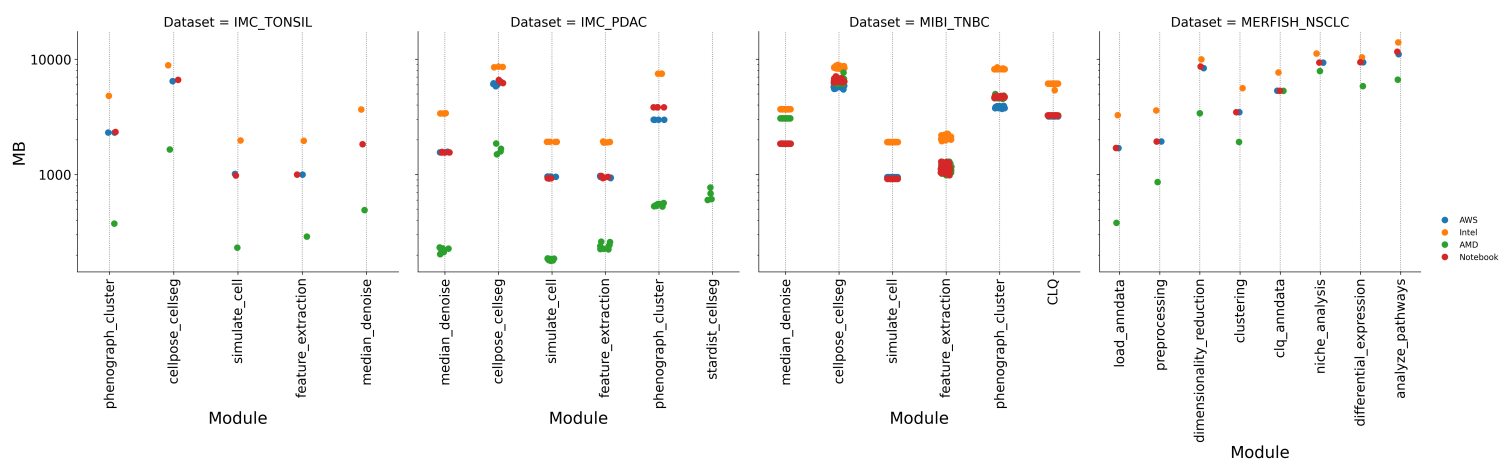

D

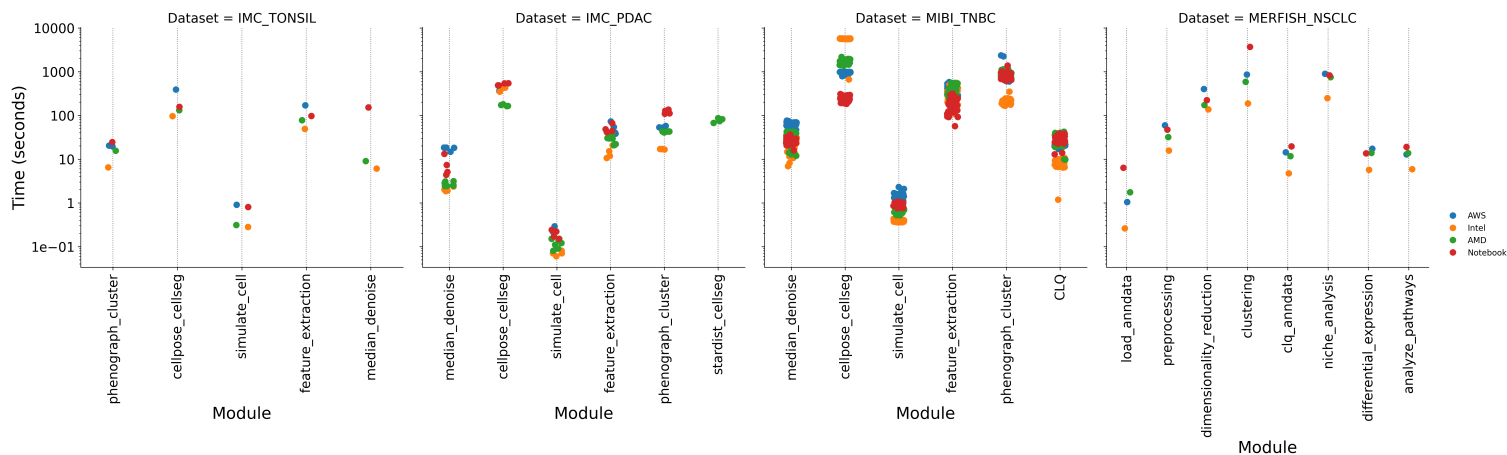

Supplement: giaf090_Supplemental_Files [file giaf090_supplemental_files.zip › FigureS1.pdf]
