# Supplementary material for: SPEX: A modular end-to-end platform for high-plex tissue spatial omics analysis
Source: Gigascience. 2025 Aug 29;14:giaf090. doi: 10.1093/gigascience/giaf090 (PMC12395962; doi:10.1093/gigascience/giaf090)
Supplement: giaf090_GIGA-D-25-00022_Revision_1 [file giaf090_giga-d-25-00022_revision_1.pdf]

GigaScience

SPEX: A modular end-to-end platform for high-plex tissue spatial omics analysis

--Manuscript Draft--

**Manuscript Number:**	GIGA-D-25-00022R1
**Full Title:**	SPEX: A modular end-to-end platform for high-plex tissue spatial omics analysis
**Article Type:**	Technical Note
**Funding Information:**	
**Abstract:**	Recent advancements in transcriptomics and proteomics have opened the possibility for spatially resolved molecular characterization of tissue architecture with the promise of enabling a deeper understanding of tissue biology in either homeostasis or disease. The wealth of data generated by these technologies has recently driven the development of a wide range of computational methods. These methods have the requirement of advanced coding fluency to be applied and integrated across the full spatial omics analysis process thus presenting a hurdle for widespread adoption by the biology research community. To address this, we introduce SPEX (Spatial Expression Explorer), a web-based analysis platform that employs modular analysis pipeline design, accessible through a user-friendly interface. SPEX's infrastructure allows for streamlined access to open source image data management systems, analysis modules, and fully integrated data visualization solutions. Analysis modules include essential steps covering image processing, single-cell and spatial analysis. We demonstrate SPEX's ability to facilitate the discovery of biological insights in spatially resolved omics datasets from healthy tissue to tumor samples.
**Corresponding Author:**	Rajiv Jesudason<br>Genentech Inc<br>South San Francisco, UNITED STATES
**Corresponding Author Secondary Information:**	
**Corresponding Author's Institution:**	Genentech Inc
**Corresponding Author's Secondary Institution:**	
**First Author:**	Xiao Li
**First Author Secondary Information:**	
**Order of Authors:**	Xiao Li
	Ximo Pechuan-Jorge
	Tyler Risom
	Conrad Foo
	Alexander Prilipko
	Artem Zubkov
	Caleb Chan
	Patrick Chang
	Frank Peale
	James Ziai
	Sandra Rost
	Derrek Hibar
	Lisa McGinnis
	Evgeniy Tabatsky

Powered by Editorial Manager® and ProduXion Manager® from Aries Systems Corporation

	Xin Ye
	Hector Corrada Bravo
	Zhen Shi
	Malgorzata Nowicka
	Jon Scherdin
	James Cowen
	Jennifer Giltnane
	Darya Orlova
	Rajiv Jesudason
**Order of Authors Secondary Information:**	
**Response to Reviewers:**	[Reviewer responses are attached as a pdf document and also listed point by point here]<br><br>Dear Editorial Board,<br>Below are our responses to the reviewers' comments. We sincerely appreciate the reviewers' thoughtful and thorough evaluation. We have addressed all concerns to the best of our ability and look forward to your feedback.<br>For your convenience, we have highlighted the changes in the revised manuscript in blue.<br>Reviewer #1:<br><br>Comment: The reviewer would like to request clarifications on how extensible the modules are. The author mentioned a SPEX pipeline builder in which "modules are selected from a library and dragged into a visual pipeline map", and also mentioend the support for "flexible plug-in analysis modules".What are the packages available from the library? Can users import their own code or script or package? How to create new plug-in's?<br>Response:<br>In response to the reviewer’s request, we have added Supplementary Table 2, SPEX Analysis Modules, which lists all currently implemented algorithms, categorized by their intended use (e.g., segmentation, clustering, etc.).<br>Each module within the SPEX user interface is backed by a corresponding Python script. As an open-source platform, SPEX provides developers with full access to these scripts, allowing them to modify and extend functionalities as needed. This open framework ensures that SPEX can continuously evolve in step with the latest advancements in spatial omics analysis.<br>SPEX also supports the integration of fully custom modules. Users can develop their own Python-based analytical modules and seamlessly integrate them into the SPEX platform. To facilitate this, we have added detailed documentation to our GitHub repository (https://github.com/Genentech/SPEX?tab=readme-ov-file#project-structure), including step-by-step instructions and a template for creating custom modules. This guide outlines how to define module inputs and outputs to ensure compatibility with the existing SPEX architecture. Additionally, we have updated the manuscript to include a more comprehensive description of the plug-in process.<br>“Custom Analysis Modules<br>To accommodate the growing collection of spatial omics methods emerging in the field, SPEX supports the integration of custom plug-in modules. Users can develop their own Python-based analytical modules and integrate them into the SPEX platform for execution within a pipeline. The primary components of a custom module is (1) an app.py file which contains the core function - reading data, processing it and returning results and (2) a json file which defines the required parameters, pipeline execution order, dependencies and other global settings. Detailed documentation along with templates can be found on our GitHub repository (https://github.com/Genentech/SPEX?tab=readme-ov-file#project-structure). This guide outlines how to define module inputs and outputs to ensure compatibility with the existing SPEX architecture.“<br>Comment: The reviewer is also wondering how do the users interact with the results? Can the user click on the resulting image and select regions of interest to zoom in?

Response:
SPEX supports interactive visualization through seamless integration with the open-source platform Vitessce (Keller et al, 2024), which enables dynamic exploration of spatial data. Users can interact directly with the resulting images, for example, by clicking to select regions of interest, which can then be zoomed in for more detailed inspection. This facilitates focused analysis of specific tissue areas. Vitessce also supports cross-filtering between the image view, UMAP projections, and bar plots. These interactive features enhance the exploratory capabilities of SPEX, allowing users to gain deeper insights from their spatial omics data.
To highlight this functionality of SPEX, we have added the following statement to our manuscript:
"Visualization
The final step in the pipeline building interface is data visualization. Here, users can visualize analytical readouts using an integrated Vitessce spatial omics dashboard. Vitessce, as described by Keller et al. (2024), is an advanced web-based platform designed for the interactive visualization of spatial omics data. The dashboard integrates a variety of visualization modalities, such as spatial plots, scatterplots, and expression heatmaps, histograms and more. All visualization panes are interactive and have cross-filtering functionality (Figure 2d). As an example, a user can select a specific population of cells in the UMAP embedded space and these same cells will be highlighted in the spatial plot atop the multiplex imaging data. The image pane can be zoomed and panned to further explore the tissue microenvironment. Together, these plots facilitate investigation of spatial patterns, cell clustering, and tissue organization at single-cell resolution. "

Reviewer #2:

Comment: The manuscript presents an advancement in spatial omics analysis but needs improvements in Quantitative benchmarking, Computational scalability assessment, etc. With these revisions, SPEX has the potential to become a widely adopted platform in the spatial omics community. I have specific comments as below:
While the manuscript provides a qualitative comparison of SPEX with other spatial omics tools (e.g., Squidpy, Giotto, Aquilla), quantitative benchmarking is missing. It is needed to include a performance benchmark comparing runtime efficiency, segmentation accuracy, and clustering resolution against existing tools. Also, it is necessary to show computational efficiency metrics (e.g., memory usage, execution time, scalability across datasets of varying sizes)

Response:

We thank the reviewer for their insightful comments and fully agree on the importance of quantitative benchmarking in evaluating analytical tools. In the supplementary materials, we have included Figure S1 that presents performance benchmarks across several datasets analyzed using SPEX. This figure highlights key metrics such as runtime efficiency, memory usage, and scalability across the diverse datasets described in the manuscript.

We would like to emphasize that the primary goal of SPEX is not to compete with existing segmentation, clustering, or other analytical algorithms, but rather to address a key gap in the spatial omics field—the limited availability of user-friendly, GUI-based tools that enable end-to-end analysis of spatially resolved omics data. We recognize that the most appropriate choice of segmentation, clustering, or other algorithms is often task- and dataset-specific. Therefore, our aim is to provide a flexible platform where users can easily plug in the algorithms of their choice and operate them within a GUI-based environment. Please refer to the 'GUI or code-based' field of Supplementary Table 1, which defines how far a user without coding expertise can progress using currently available platforms.
While SPEX includes and supports a set of default algorithms-each of which has been benchmarked in the original studies where they were introduced-we would like to reiterate that these are provided primarily for illustrative purposes. However, we have now included a justification for the specific algorithms selected as defaults. The following statements have been added to the manuscript: "For proteomics, we also include the widely adopted graph-based clustering algorithm, PhenoGraph, which has recently been shown to be among the most robust to the curse of dimensionality

compared to other high-dimensional clustering methods14."
In the case of CLQ, while its global version has been previously studied, for example, in the CELESTA framework developed by Zhang, Weiruo, et al. (2022), its local variant has not yet been explored in this context. Therefore, we conducted a side-by-side benchmarking of the global CLQ versions, and our results show that its performance is comparable to the current state of the art. Importantly, we have also introduced a local CLQ, which is unique; by design, it outperforms the global CLQ by enabling the investigation of heterogeneity in patterns that would otherwise remain undetectable at the average cell population level. These results are now included in the supplementary materials.

We have also updated the manuscript with the following description of the plug-in process:

"Custom Analysis Modules

To accommodate the growing collection of spatial omics methods emerging in the field, SPEX supports the integration of custom plug-in modules. Users can develop their own Python-based analytical modules and integrate them into the SPEX platform for execution within a pipeline. The primary components of a custom module are (1) an app.py file which contains the core function which covers reading input data, processing it and returning results and (2) a json file which defines the required interactive parameters, pipeline execution order, dependencies and other global settings. Detailed documentation along with templates can be found on our GitHub repository (https://github.com/Genentech/SPEX?tab=readme-ov-file#project-structure). This guide outlines how to define module inputs and outputs to ensure compatibility with the existing SPEX architecture."

Comment: The study presents compelling results, but there is no independent validation or interpretation of computational outputs using experimental methods.

Response:

We thank the reviewer for this important comment. This manuscript focuses on introducing a new software analysis package and demonstrating its utility in enabling biological discovery. While some of the included results are indeed biologically compelling, validating them experimentally is beyond the scope of this work.

To address this, we have revised the manuscript to soften the language regarding the ability to draw definitive biological conclusions based solely on the computational outputs. We have also more explicitly stated that analysis outcomes were evaluated for robustness either through comparison with prior analyses of the same dataset (e.g., Keren et al.) or, in cases where independent analyses were not available, through expert review by experienced pathologists (e.g., IMC data).

Comment: The manuscript does not discuss hardware requirements, processing speed, or computational limitations. It is needed to provide an assessment of SPEX's performance on different computing environments (e.g., local workstations vs. cloud computing vs. high-performance clusters).

Response:

We agree with the reviewer's suggestion and have now incorporated the relevant information into the manuscript. We have included a benchmarking of pipeline execution for all reported datasets using several computing environments which include, AWS, INTEL, AMD, and local notebook execution. We have also included the resource specification details for each environment in the manuscript. Suggested minimal system requirements have been added to the application github repository https://github.com/Genentech/SPEX. The following paragraph has been added to the manuscript:

"To ensure SPEX can be deployed on a variety of environments and provide recommendations for resource specifications we benchmarked execution of analysis pipelines for the reported datasets across 4 different compute environments (Fig S1 (C) and (D)). This included, Intel (CPU: Intel i5-13500, 14 cores / 20 threads, 800–4800 MHz, Memory: 62 GB RAM), AMD (CPU: AMD64 Family 25 Model 117, ~3.8 GHz, 1 processor, virtualization enabled, Memory: 62 GB RAM, Virtual Memory: 97 GB total), AWS (CPU: AMD EPYC 7571, 8 cores / 16 threads, base ~3.2 GHz, Memory: 62 GB RAM), and a notebook computer (CPU: AMD Ryzen 7 5800H, 8 cores / 16 threads, base 3.2 GHz, Memory: 45 GB RAM). Both processing time and memory usage for specific modules are reported. Single-cell segmentation and clustering represented the most memory intensive steps of the pipeline. This time scaled with dataset size and

complexity. Segmentation is inherently memory-intensive because it involves processing large, multi-dimensional image data, often requiring the simultaneous loading of multiple image tiles or channels into memory for normalization and inference. Clustering, on the other hand, operates on large, high-dimensional single-cell feature matrices. Memory usage grows rapidly as the number of cells and features increases, since many clustering algorithms require storage of distance matrices or neighborhood graphs.“

Comment: The Colocation Quotient (CLQ) method is well described, but the manuscript does not provide statistical validation (e.g., p-values, confidence intervals) for detected spatial relationships.

Response:

We sincerely thank the reviewer for highlighting the importance of statistical validation for the Colocation Quotient (CLQ) method.
In SPEX implementation, we have implemented permutation-based hypothesis testing to evaluate the significance of spatial associations. Below are the key details of our approach:
Permutation Testing Framework:
Null Hypothesis: Spatial associations between cell types are random (i.e., no systematic attraction/avoidant).
Procedure:
Spatial coordinates of cells were fixed to preserve tissue architecture.
Cell type labels were randomly shuffled across cells (e.g. 999 permutations) to simulate spatial randomness under the null hypothesis.
The observed CLQ was included in the null distribution, resulting in a total of 1,000 values(999 permutations + observed CLQ).
Empirical P-value Calculation:
P-values were computed as:
Pattractive=count(permuted CLQs>=observed CLQ)+1/(1+999)
Pavoidant=count(permuted CLQs<=observed CLQ)+1/(1+999)
This “+1” adjustment ensures that the observed CLQ is part of its own null

distribution, preventing p-values of zero and aligning with best practices in non-parametric testing.
Significance Thresholds:
Attractive: Pattractive 0.05 (observed CLQ ≥ 95% of permuted CLQs).
Avoidant: Pavoidant 0.05 (observed CLQ ≤ 95% of permuted CLQs).
Relationships not meeting these thresholds were classified as non-significant.

We also updated the method section to strengthen the statistical rigor of our analysis.

Reviewer #3:

Comment: One of the critical omissions in this manuscript is the lack of rigorous benchmarking against established tools. Though it demonstrated the comparison with other tools such as Squidpy, Giotto, and MC Micro, but there is no quantitative comparison to demonstrate its advantages over existing methodologies. In particular, spatial analysis such as CLQ is introduced as a different approach within the spatial biology analytics framework, but how does it compare to existing co-occurrence analysis methods? Additionally, similar analyses have been conducted using other tools (e.g., Mah, C.K., et al., Genome Biol 25, 82 (2024)), including in 'subcellular' colocalization. In this regard, concerns about its novelty arise. Moreover, as mentioned in relation to Bento, CLQ could also be applied to subcellular analysis?
- In this regard, for spatial co-occurence or other algorithms in SPEX, the authors should run identical datasets through both SPEX and existing tools to compare performance and biological insights. it is impossible to assess whether SPEX provides any meaningful improvement over existing platforms.

Response:

We would like to emphasize that the primary goal of SPEX is not to compete with existing segmentation, clustering, or other analytical algorithms, but rather to address a key gap in the spatial omics field—the limited availability of user-friendly, GUI-based tools that enable end-to-end analysis of spatially resolved omics data. We recognize that the most appropriate choice of segmentation, clustering, or other algorithms is often task- and dataset-specific. Therefore, our aim is to provide a flexible platform where users can easily plug in the algorithms of their choice and operate them within a GUI-based environment. Please refer to the 'GUI or code-based' field of Supplementary Table 1, which defines how far a user without coding expertise can progress using currently available platforms.

While SPEX includes and supports a set of default algorithms-each of which has been benchmarked in the original studies where they were introduced-we would like to reiterate that these are provided primarily for illustrative purposes. However, we have now included a justification for the specific algorithms selected as defaults. The following statements have been added to the manuscript: "For proteomics, we also include the widely adopted graph-based clustering algorithm, PhenoGraph, which has recently been shown to be among the most robust to the curse of dimensionality compared to other high-dimensional clustering methods [https://www.biorxiv.org/content/10.1101/2024.04.18.589981v1]."

In the case of CLQ, while its global version has been previously studied, for example, in the CELESTA framework developed by Zhang, Weiruo, et al. (2022), its local variant has not yet been explored in this context. Therefore, we conducted a side-by-side benchmarking of the global CLQ versions, and our results show that its performance is comparable to the current state of the art. Importantly, we have also introduced a local CLQ, which is unique; by design, it outperforms the global CLQ by enabling the investigation of heterogeneity in patterns that would otherwise remain undetectable at the average cell population level. These results are now included in the supplementary materials.

We have also updated the manuscript with the following description of the plug-in process:

"Custom Analysis Modules

To accommodate the growing collection of spatial omics methods emerging in the field, SPEX supports the integration of custom plug-in modules. Users can develop their own Python-based analytical modules and integrate them into the SPEX platform for execution within a pipeline. The primary components of a custom module is (1) an app.py file which contains the core function - reading data, processing it and returning results and (2) a json file which defines the required parameters, pipeline execution order, dependencies and other global settings. Detailed documentation along with templates can be found on our GitHub repository (https://github.com/Genentech/SPEX?tab=readme-ov-file#project-structure). This guide outlines how to define module inputs and outputs to ensure compatibility with the existing SPEX architecture."

Comment: The cell typing process is one of the most fundamental steps in spatial omics analysis. However, SPEX does not integrate a dedicated cell typing module, forcing users to use another tool or define cell types manually. The accuracy of all downstream analyses (clustering, spatial interaction, pathway analysis) depends on robust and reliable cell typing. It would be better to integrate with automated cell typing solutions to increase usability.

Response:

We thank the reviewer for highlighting the importance of cell typing in spatial omics analysis and for pointing out the need for more explicit clarification in our manuscript. After unsupervised clustering is executed, clusters can be renamed and grouped to facilitate more meaningful downstream spatial analysis within the SPEX application. To facilitate annotation of clusters to biologically relevant cell types, SPEX offers a manual classification module where users can easily rename cluster identifiers. The fully integrated Vitessce spatial omics visualization dashboard aids in this process by providing a means to interpret the expression profile of the cluster through heatmaps. Vitessce spatial plots can also aid in proper cluster classification as certain cell types may have specific localization patterns. One can also export the SPEX clustering results in anndata format and leverage a wide variety of cell type annotation tools available in Python or R. For analysis of the NSCLC MERFISH data, we utilized the Pegasus python package for cell typing.

We do agree that a fully integrated automated cell typing routine would be beneficial,

Powered by Editorial Manager® and ProduXion Manager® from Aries Systems Corporation

therefore we again note that users have flexibility in implementing algorithms from established packages like Pegasus or CellTypist as customized plugins. This design empowers users to identify and incorporate the most appropriate approach for their data.
To address the reviewer's concern, we have expanded the description of the cell annotation process in the manuscript and added Supplementary Table 2, which provides a comprehensive overview of all currently implemented algorithms in SPEX, categorized by their intended use (e.g., segmentation, phenotyping, etc.). We believe this addition will help clarify how users can perform or incorporate cell typing workflows within the SPEX framework.
Comment: The manuscript focuses almost exclusively on single-cell resolution data and high-dimensional imaging-based methods (e.g., IMC, MIBI, MERFISH). However, spot-based transcriptomics platforms such as Visium are widely used in the field. In this regard, SPEX does not provide modules tailored methodology for spot-based spatial analysis (such as deconvolution) or super-resolution or transforming cell-based analysis from spots (e.g. bin2cell in VisiumHD). Neighborhood analyses or spatially variable gene detection, etc. are specialized in whole-gene covered, spot-based methods, as well, for example.

Response:
We thank the reviewer for this important observation. Indeed, the current version of SPEX has been primarily designed and benchmarked using single-cell resolution and high-dimensional imaging-based datasets. However, we agree that spot-based transcriptomics platforms such as Visium are widely used and require tailored analytical approaches.
While SPEX does not currently include built-in modules for cell-type deconvolution or spatial super-resolution, it can be used downstream of preprocessing tools such as bin2cell, which convert spot-level data into approximated single-cell resolution. Once such transformation is performed, users can leverage SPEX's existing suite of modules for neighborhood analysis, clustering, and spatial pattern detection.
We recognize the importance of expanding SPEX's capabilities to natively support spot-based analyses and plan to explore integration of such functionality in future releases. To clarify this limitation and the potential workaround, we have revised the manuscript to include the following statement:
"SPEX modules
The SPEX graphical user interface provides essential components to facilitate comprehensive analysis of spatial omics images and datasets SPEX supports end to end modules for spatial proteomics - covering both image processing and downstream single-cell spatial analysis. Since the vast majority of single-cell spatial transcriptomics platforms output cell by transcript files with spatial coordinates, SPEX will also directly ingest a cell by transcript Anndata file (.h5ad) for downstream clustering and/or single-cell spatial analysis. For non-imaging, sequencing-based spatial transcriptomics platforms such as 10x Genomics Visium HD, users can leverage existing tools like bin2cell to transform bin-level data into pseudo single-cell resolution. The transformed data, structured as a cell-by-gene matrix with spatial coordinates, can then be converted into an AnnData object and processed by SPEX for downstream analyses such as clustering, spatial feature extraction, and colocation analysis"
Comment: The manuscript does not clarify whether users can modify or extend the pipeline with custom Python scripts. Describing further this point, customization in this ecosystem with python script, for 'power-users' of this system could be helpful.
Response:
We thank the reviewer for this helpful suggestion. Each module within the SPEX user interface is backed by an underlying Python script. As an open-source platform, SPEX provides developers with full access to these scripts, enabling them to modify and extend functionalities as needed. This openness ensures that SPEX can continually evolve alongside advancements in spatial omics analysis. SPEX also supports the integration of fully custom modules. Users can develop their own Python-based analytical components and seamlessly incorporate them into the platform. To facilitate this, we have added detailed documentation to our GitHub repository, including step-by-step instructions and a template for creating custom modules. This guide walks users through the process of defining module inputs and outputs to ensure compatibility with the existing SPEX architecture. We have now incorporated a statement reflecting this functionality into the manuscript.

Powered by Editorial Manager® and ProduXion Manager® from Aries Systems Corporation

Comment: The biological relevance of the SPEX platform remains unclear, as the case studies presented are not sufficiently rigorous. As mentioned above, comparisons with other tools based on quantification can clarify why SPEX is better than other published tools/ecosystems in certain aspect. Or meaningful biological findings and explanations based on this tool as a case study could be helpful. While the results demonstrate technical capabilities, the manuscript does not show how SPEX enables novel biological discoveries compared to existing tools.

Response:

We thank the reviewer for these thoughtful suggestions and for recognizing that the results presented in our manuscript adequately demonstrate the technical capabilities of the SPEX platform. While presenting a novel biological discovery is beyond the scope of this manuscript, we have now emphasized the strong agreement between SPEX analysis results and previously published data (Keren et al.).
Please find the following statement under “SPEX Spatial Proteomics Pipeline Enables Novel Single Cell Spatial Insights” subsection in the “Results” section:

“Following cell phenotyping by unsupervised clustering these SPEX modules generated cell calls that correctly match their molecular profiles and the published calls by the original work3 (Figure 4b-d). Through arranging the tumors from low to high immune cell frequency of total cells, we observe an increased diversity of immune cell types within the sample where samples with a lower density of immune cells are dominated by macrophages and samples with higher density of immune cells have a mixed composition of lymphocytes, myeloid cells and antigen presenting cells (Figure 4d). To further interrogate the spatial relationship between this variable immune compartment and the tumor cells, we employ the Colocation Quotient (CLQ) method under the SPEX Spatial co-occurrence module. Briefly, the CLQ measures the co-occurrence or avoidance of cell type pairs by looking at the local density of a target cell type at a fixed radius from each cell of the sample belonging to a reference cell type. That way, one can, for instance, query if target immune cells are co-locating or avoiding tumor cells. We applied the CLQ module with the immune cell::tumor cell pair here in samples that had more than 10% immune cells (non-desert samples) revealing two major groups of samples, those with high immune::tumor cell spatial enrichment, and those with low immune:tumor cell enrichment (Figure 4e). These identified inflamed and excluded groupings aligned well with the visual representation of immunophenotyping presented in the images (Figure 4f).”

We would like to emphasize that the primary goal of SPEX is not to compete with existing segmentation, clustering, or other analytical algorithms, but rather to address a key gap in the spatial omics field—the limited availability of user-friendly, GUI-based tools that enable end-to-end analysis of spatially resolved omics data. We recognize that the most appropriate choice of segmentation, clustering, or other algorithms is often task- and dataset-specific. Therefore, our aim is to provide a flexible platform where users can easily plug in the algorithms of their choice and operate them within a GUI-based environment. Please refer to the ‘GUI or code-based’ field of Supplementary Table 1, which defines how far a user without coding expertise can progress using currently available platforms.
While SPEX includes and supports a set of default algorithms-each of which has been benchmarked in the original studies where they were introduced-we would like to reiterate that these are provided primarily for illustrative purposes. However, we have now included a justification for the specific algorithms selected as defaults. The following statements have been added to the manuscript: “For proteomics, we also include the widely adopted graph-based clustering algorithm, PhenoGraph, which has recently been shown to be among the most robust to the curse of dimensionality compared to other high-dimensional clustering methods [https://www.biorxiv.org/content/10.1101/2024.04.18.589981v1].”
In the case of CLQ, while its global version has been previously studied, for example, in the CELESTA framework developed by Zhang, Weiruo, et al. (2022), its local variant has not yet been explored in this context. Therefore, we conducted a side-by-side benchmarking of the global CLQ versions, and our results show that its performance is comparable to the current state of the art. Importantly, we have also introduced a local CLQ, which is unique; by design, it outperforms the global CLQ by enabling the investigation of heterogeneity in patterns that would otherwise remain undetectable at

	the average cell population level. These results are now included in the supplementary materials.<br>We have also updated the manuscript with the following description of the plug-in process:<br>“Custom Analysis Modules<br>To accommodate the growing collection of spatial omics methods emerging in the field, SPEX supports the integration of custom plug-in modules. Users can develop their own Python-based analytical modules and integrate them into the SPEX platform for execution within a pipeline. The primary components of a custom module is (1) an app.py file which contains the core function - reading data, processing it and returning results and (2) a json file which defines the required parameters, pipeline execution order, dependencies and other global settings. Detailed documentation along with templates can be found on our GitHub repository (https://github.com/Genentech/SPEX?tab=readme-ov-file#project-structure). This guide outlines how to define module inputs and outputs to ensure compatibility with the existing SPEX architecture.“
Additional Information:	
Question	Response
Are you submitting this manuscript to a special series or article collection?	No
**Experimental design and statistics**<br><br>Full details of the experimental design and statistical methods used should be given in the Methods section, as detailed in our Minimum Standards Reporting Checklist. Information essential to interpreting the data presented should be made available in the figure legends.<br><br>Have you included all the information requested in your manuscript?	Yes
**Resources**<br><br>A description of all resources used, including antibodies, cell lines, animals and software tools, with enough information to allow them to be uniquely identified, should be included in the Methods section. Authors are strongly encouraged to cite Research Resource Identifiers (RRIDs) for antibodies, model organisms and tools, where possible.<br><br>Have you included the information requested as detailed in our Minimum Standards Reporting Checklist?	Yes

Powered by Editorial Manager® and ProduXion Manager® from Aries Systems Corporation

**Availability of data and materials**<br><br>All datasets and code on which the conclusions of the paper rely must be either included in your submission or deposited in publicly available repositories (where available and ethically appropriate), referencing such data using a unique identifier in the references and in the “Availability of Data and Materials” section of your manuscript.<br><br>Have you have met the above requirement as detailed in our Minimum Standards Reporting Checklist?	Yes
GigaScience has policies and guidelines in place for the use of generative AI-writing tools such as ChatGPT. If you have used such writing tools to assist with writing the manuscript this must be declared and cited in the text. Authors should not list AI-writing tools and other AI-assisted technologies as an author or co-author and should acknowledge that they are fully responsible for text generated or refined by AI-writing tools.<p><br><br>A summary of use (particularly in the introduction or among methods) needs to be included at the end of the paper, and the outputs should also be included as a supplementary file hosted in GigaDB or other open repositories. Please read our guidelines for more information. <p><br><br>By submitting to GigaScience, you are aware of the journal's AI-writing tools policy, and if you have declared use of such tools below, you have acknowledged this where appropriate in your manuscript and have made a summary of use and outputs available. <p><br><b>AI-assisted writing tools have been used in the preparation of this manuscript?	No

manuscript Click here to access/download;Manuscript;GigaScience_SPEX_rev_final.pdf

SPEX: A modular end-to-end platform for high-plex tissue spatial omics analysis

Xiao Li[1,*], Ximo Pechuan-Jorge[1,*], Tyler Risom[1], Conrad Foo[1], Alexander Prilipko[1], Artem Zubkov[1], Caleb Chan[1], Patrick Chang[1], Frank Peale[1], James Ziai[1], Sandra Rost[1], Derrek Hibar[1], Lisa McGinnis[1], Evgeniy Tabatsky[1], Xin Ye[1], Hector Corrada Bravo[1], Zhen Shi[1], Malgorzata Nowicka[1], Jon Scherdin[1], James Cowan[1], Jennifer Giltnane[1], Darya Orlova[1,†,∇], and Rajiv Jesudason[1,†,∇]

[1]Genentech, Inc., South San Francisco, 94080, CA, USA
[*]Denotes co-first author.
[†]Denotes co-senior author.
[∇]To whom correspondence should be addressed: dyorlova@gmail.com, jesudason.rajiv@gene.com

ABSTRACT

Recent advancements in transcriptomics and proteomics have opened the possibility for spatially resolved molecular characterization of tissue architecture with the promise of enabling a deeper understanding of tissue biology in either homeostasis or disease. The wealth of data generated by these technologies has recently driven the development of a wide range of computational methods. These methods have the requirement of advanced coding fluency to be applied and integrated across the full spatial omics analysis process thus presenting a hurdle for widespread adoption by the biology research community. To address this, we introduce SPEX (Spatial Expression Explorer), a web-based analysis platform that employs modular analysis pipeline design, accessible through a user-friendly interface. SPEX's infrastructure allows for streamlined access to open source image data management systems, analysis modules, and fully integrated data visualization solutions. Analysis modules include essential steps covering image processing, single-cell and spatial analysis. We demonstrate SPEX's ability to facilitate the discovery of biological insights in spatially resolved omics datasets from healthy tissue to tumor samples.

Introduction

Spatially resolved, highly multiplexed protein and RNA profiling of tissues at the cellular or sub-cellular level is essential for understanding the molecular components underlying tissue architecture and function[1]. This fine characterization enables the measurement of cellular heterogeneity and cell-cell interactions in the spatial dimension which in turn brings us closer to identifying molecular determinants of tissue function. Hence, recently developed technologies, like IMC[2], MIBI[3], CODEX[4] or MERFISH[5], that allow for the acquisition of spatially resolved proteomic and transcriptomic states of individual cells and tissues, hold a great promise for advancing our understanding in many areas of cell and developmental biology. However, the widespread adoption of these technologies vitally depends on the development of analytical capabilities and infrastructure to readily extract biologically relevant conclusions from the complex data generated.

Spatially resolved omics data contain multiple layers of information which in turn are derived from imaging data and the molecular measurements associated with it. Examples of said layers constitute cell morphology, patterns of protein and transcript expression, cell neighborhoods or cell-cell communication at different spatial scales. To obtain this information, robust pre-processing and visualization methods are required as well as ways of analyzing the omics information and integrating it with the image derived properties[6]. The current software ecosystem developed in the past five years leverages several common analysis categories, including cell segmentation, clustering and spatial analytics, all required for meaningful interpretation of spatially resolved omics data[7,8].

However, currently existing tools and custom analysis protocols provide only fragmentary solutions to the spatial omics analytical workflow[9,10]. None of them standing alone provide means for efficient data representation and pre-processing, interactive visualizations, and spatial relationship querying. As a result, most current end-to-end analytical strategies involve piecemeal workflows requiring data migration across numerous programs and coding proficiency. To our knowledge, the code-free platforms currently available are predominantly developed by commercial spatial omics platform vendors. These software solutions are user-friendly but often entail prohibitive licensing agreements and associated costs that hinder widespread use. Moreover, these commercial solutions demonstrate limitations in their applicability across various modalities or platforms. Every imaging, transcriptomic, and proteomic data type has unique interpretation requirements, undercutting the effectiveness of

platforms optimized for specific vendor data types. Therefore, the scientific community's demand is twofold: for an open-source solution to democratize access and foster enhanced collaboration and reproducibility; and for a flexible, comprehensive tool that can be tailored to different modalities and platforms without compromising user accessibility and functionality.

With these current challenges in mind, we developed SPEX (Spatial Expression Explorer), a modular analytics platform that covers a broad span of essential methods required to analyze spatial omics data. SPEX is an application that could readily be deployed on cloud, or on a local workstation. It has a unique architecture that allows users to build data modality-specific customized pipelines from SPEX's novel analytics developments or via plugging in external open source analysis modules. To support the needs of the cross-functional research community we designed SPEX in a way that one could operate it either from the user interface (code-free environment) or via more involved hands-on coding. A detailed comparison of SPEX with existing tools is provided in Supplementary Table 1, assessing key aspects such as execution mode (GUI vs. code-based), target audience and prerequisites, supported data types, interactive image visualization, image preprocessing, single-cell segmentation, cell type clustering, spatial analysis, image data management, and data analytics visualization.

Here, as part of the SPEX software package, we implement extensible methods for image preprocessing, single-cell segmentation, segmentation post-processing, single-cell clustering, cell-cell co-occurrence, niche/neighborhood analysis, and spatially informed functional analysis in the form of differential expression analysis and pathway enrichment analysis. SPEX is integrated with the open-sourced and widely adopted image management system, OMERO[11] and also leverages open-source spatial omics data visualization solution, Vitessce[12], as a fully integrated element of the graphical user interface.

As a proof of concept, we show SPEX appropriately characterizes the spatial and molecular configuration of tonsil tissue. We further demonstrate the robustness of these methods in more heterogeneous tumor microenvironments represented in Pancreatic Ductal Adenocarcinoma (PDAC) and Triple Negative Breast Cancer (TNBC). Moreover, we extended the analysis to identify new patterns in tumor-immune microenvironment composition. Lastly, in a spatial transcriptomics dataset, we applied spatially-informed pathway analysis to a human lung cancer specimen and elucidated the pathways associated with immune attraction/avoidance in immune cells and tumor cells.

Results

SPEX is a comprehensive spatial omics analysis platform implemented as a user-friendly web-based application with flexible plug-in analysis modules (Figure 1, Supplementary Table 2). SPEX provides both infrastructure and quantitative analysis methods that allow for efficient storage (Figure 1a), manipulation and interactive visualization of spatial expression data (Figure 1e). SPEX has an easy to use graphical user interface which allows the wider research community to build analytical pipelines and visualize high dimensional spatial data.

SPEX Analytical Modules

For spatial proteomics modalities, SPEX can start with raw imaging data or single-cell objects with spatial coordinates. For spatial transcriptomics modalities, SPEX requires a single-cell object with spatial coordinates as inputAnalysis can then be performed on these assets by leveraging a wide range of algorithms that can be categorized into four primary domains- image processing (spatial proteomics), single-cell clustering, cell-cell interaction spatial analysis and spatially informed differential expression analysis and pathway analysis.

Image Processing Modules

SPEX includes a modular pipeline to facilitate tissue-based single-cell segmentation (Figure 1c). This generalized pipeline aims to accommodate a wide range of high-dimensional imaging modalities such as IMC, MIBI, cyclic IF and spatial transcriptomics . Image processing is executed in a 4 step sequence with the ultimate goal of generating a cell by expression matrix in Anndata format for downstream single-cell and spatial analysis. These steps include image preprocessing, single-cell segmentation, post-processing and feature extraction. Each step contains a selection of modules which can be linked together to address the particularities of a given image set. The adaptability of the pipeline was demonstrated by analyzing imaging data coming from a variety of high dimensional imaging modalities. Each imageset presented unique characteristics; varying in resolution, signal to noise and structural attributes.

Clustering Module

To enable the analysis of the high-plex omics data associated with the images, SPEX provides single-cell clustering modules that cover both intensity-based proteomics and count-based transcriptomics single-cell inputs. For transcriptomics, we rely on the suite of proven methods offered by the Pegasus package[13]. For proteomics, we also include the widely adopted graph-based clustering algorithm, PhenoGraph, which has recently been shown to be among the most robust to the curse of dimensionality compared to other high-dimensional clustering methods[14].

Spatial Analysis Module

Tissue architecture can be complex, with cell types forming spatial patterns that define particular domains where they might exert very different functions owing to the distinct local cellular context. These functions might be reflected in the associated gene or protein expression patterns. SPEX implements the Colocation Quotient (CLQ) module, as detailed in the methods section, which integrates spatial coordinates of the identified cells with their respective cell types to stratify them into co-location or avoidance pattern between cell types. Further, given a set of spatially co-occurring cells, it is natural to ask whether these co-occurrence patterns are repeated throughout the tissue, and what comprises such patterns or spatial niches. Clustering spatial co-occurrence features (in an unsupervised fashion) facilitates the identification of such spatial patterns or niches.

Spatial expression analysis Module

The functional state of cells and/or their molecular action may be influenced by their spatial organization in tissue. This can be due to inclusion in higher order functional structures or cell-cell mediated interactions. To facilitate quantification of spatially informed expression, SPEX includes both differential expression analysis and pathway analysis. These modules can take spatially informed cell categories as input

SPEX User Interface

The SPEX graphical user interface is designed to minimize spurious user options and visual overhead in an effort to increase platform intuitiveness. At a high-level the SPEX interface can be divided into 3 primary sections organized as sequential steps- project creation, data loading, and analysis development (Figure 2). The analysis section contains sequential sub-sections covering pipeline building, batch execution and data visualization (Figure 2c). Critically, these application sections can be executed without the need for code scripting ensuring a user-friendly and efficient analysis experience within the SPEX environment.

Project Creation

After logging into SPEX, the first screen encountered by users is the SPEX project creation interface (Figure 2a), where projects, serving as containers for study data, are initiated. The user can define a name and description for the project for future retrieval. This project can then be accessed and populated with omics data to be analyzed.

Data Loading

SPEX supports analysis of one of three data formats- OMETIFF, OMEZARR or H5AD (Anndata). OMETIFF and OMEZARR represent supported multiplex image formats while H5AD represents the supported single-cell data format. H5AD files can be loaded from local storage drives. The integration with an OMERO image management system (Figure 2b) allows seamless visual navigation and selection of OMERO-hosted imaging data within SPEX. Individual images across OMERO datasets can be selected, concatenated and downloaded into the SPEX project for downstream analysis.

Analysis Development

Once a project is populated with image or single-cell data, the user can define an analysis pipeline. Several pipelines can exist for any given project if the user is interested in prototyping different analysis workflows. To develop an analytical workflow, users engage with the SPEX pipeline builder interface (Figure 2c). The user progresses In the build section enabling the graphical construction of analysis pipelines. In this phase, modules are selected from a library section and dragged into a visual pipeline map (Figure 1c). The selection of modules are dependency-informed, where new modules can only be added to modules that generate pre-requisite data, thus eliminating the potential for erroneous pipeline construction. Modules with user-defined parameters include graphical widgets such as slidebars and dropdown menus displayed in a window to facilitate code-free user parameterization. Once the user has constructed a pipeline and defined the parameters, they move onto execution. In the execution page of the pipeline builder, the user can send the data for batch processing. The status of the analysis jobs will be displayed for all images in the queue. When complete, an aggregated Anndata single-cell object housing data from all images is automatically generated and available for local download as a ZARR file. This ZARR file can be loaded into other external analysis routines in Python or R (Figure 1f).

Visualization

The final step in the pipeline building interface is data visualization. Here, users can visualize analytical readouts using an integrated Vitessce spatial omics dashboard. Vitessce, as described by Keller et al. (2024)[12], is an advanced web-based platform designed for the interactive visualization of spatial omics data. The dashboard integrates a variety of visualization modalities, such as spatial plots, scatter-plots, and expression heatmaps, histograms and more. All visualization panes are interactive and have cross-filtering functionality (Figure 2d). As an example, a user can select a specific population of cells in the UMAP embedded space and these same cells will be highlighted in the spatial plot atop the multiplex imaging data. The image pane can be zoomed and panned to further explore the tissue microenvironment. Together, these plots facilitate investigation of spatial patterns, cell clustering, and tissue organization at single-cell resolution.

SPEX workflow on spatial proteomics data

SPEX identified structural composition of Tonsil

In the previous sections, we illustrated the capabilities of the different modules that integrate SPEX. To demonstrate that SPEX analysis modules generate biologically meaningful results, we validated the SPEX workflow by analyzing a sample from human tonsil tissue, which has a well-defined cellular organization[15–17]. To achieve this, a 4μm-thick section of formalin-fixed, paraffin-embedded (FFPE) tonsil tissue was stained with a panel of Imaging Mass Cytometry (IMC) antibodies listed in Supplementary Table 3. The antibody panel was designed to simultaneously characterize the composition of the immune compartment, the spatial relationship between immune cells and stromal cells, and the interactions among cell subsets.

The stained tonsil sample was imaged with the Hyperion Imaging System and then pre-processed and analyzed using a SPEX pipeline utilizing most of SPEX modules (Figure 3a). This pipeline applied to the tonsil tissue IMC images started with per cell segmentation using StarDist based on the nuclear histone H3 channel. Then, Phenograph was used for clustering the segmented cells according to their median marker expression levels of the different IMC channels. Clustering outcomes were visualized using UMAP, and cluster labels were assigned based on the IMC marker measurements as well as positional data (x and y coordinates) of cells revealing 3 well-defined lobes representing epithelial, follicular and paracortical tissue compartments distinguished primarily by expression of cytokeratin, Ki67, and CD8, respectively (Figure 3b), consistent with the composition of cells in these compartments. Within the individual UMAP regions, the location of closely related cell populations correlates with biologically relevant parameters. For example, we note distinct populations representing well characterized functional zones of tonsil germinal centers. Germinal center light and dark zones can be distinguished across an axis of Ki67 and BCL2 expression.

We applied this same SPEX workflow of single-cell segmentation, feature extraction, data normalization, and single cell phenotyping to four regions of interest in a pancreatic adenocarcinoma (PDAC) patient tumor, revealing major cell types by Phenograph, including stromal cells, T cells, myeloid cells, B cells, FDC cells, and a myriad of tumor cell states (Figure 3f). Compositional analysis of these cell states per ROI revealed two of the four ROIs encompassed robust B cell and FDC cell frequencies (Figure 3g) in a spatially co-localized pattern (Figure 3h). Upon further spatial interrogation of the molecular expression of CD3, CD20, and CD21 in these cells by image overlays (Figure 3i) and spatial heatmaps (Figure 3j,k, l) they appear to compile a Tertiary Lymphoid Structure (TLS).

SPEX Spatial Proteomics Pipeline Enables Novel Single Cell Spatial Insights

Next we analyzed a public Multiplex Ion Beam Imaging (MIBI) dataset[3] to demonstrate the single cell spatial analysis methods of the SPEX pipeline (Figure 4a). This imageset consisted of 41 field of view images with 36 channels. Being a non-optical mass detection platform, it is common for raw images to include substantial noise when compared to traditional optical acquisition platforms. Therefore, median denoising was executed on channels utilized for segmentation (dsDNA, H3K9ac, H3K27me3), channels were then merged and segmented for single-cells using the Cellpose deep learning model[18]. Following cell phenotyping by unsupervised clustering these SPEX modules generated cell calls that correctly match their molecular profiles and the published calls by the original work[3] (Figure 4b-d). Through arranging the tumors from low to high immune cell frequency of total cells, we observe an increased diversity of immune cell types within the sample where samples with a lower density of immune cells are dominated by macrophages and samples with higher density of immune cells have a mixed composition of lymphocytes, myeloid cells and antigen presenting cells (Figure 4d). To further interrogate the spatial relationship between this variable immune compartment and the tumor cells, we employ the Colocation Quotient (CLQ) method under the SPEX Spatial co-occurrence module. Briefly, the CLQ measures the co-occurrence or avoidance of cell type pairs by looking at the local density of a target cell type at a fixed radius from each cell of the sample belonging to a reference cell type. That way, one can, for instance, query if target immune cells are co-locating or avoiding tumor cells. We applied the CLQ module with the immune cell::tumor cell pair here in samples that had more than 10% immune cells (non-desert samples) revealing two major groups of samples, those with high immune::tumor cell spatial enrichment, and those with low immune:tumor cell enrichment (Figure 4e). These identified inflamed and excluded groupings aligned well with the visual representation of immunophenotyping presented in the images (Figure 4f).

Having defined the tumor immune phenotypes with CLQ analysis as desert, excluded, or inflamed tumors, we explored potential gene expression patterns yielded by this classification. We observed a significant increase in PDL1 expression by tumor-associated macrophages in inflamed tumors suggesting a relationship between tumor-immune spatial enrichment and immune checkpoint expression on tumor-associated macrophages. (Figure 4h)[19,20]. We then further leveraged the CLQ module to interrogate macrophage phenotypes in these tumors by computing their pairwise spatial enrichment with all other cell types. Interestingly, this combination of CLQ and expression analysis revealed that macrophages that are spatially enriched (attractive) with CD8+ Cytotoxic T Cells (Figure 4g) had significantly higher IDO1 expression compared to macrophages that are spatially isolated (avoidant) from these T Cells (Figure 4i). This relationship was observed in excluded tumors and further enriched in inflamed tumors, suggesting IDO1 expression by macrophages may play an immunosuppressive role specifically in the inflamed subset of patients. While conclusions on the significance of these observations would require their validation by

orthogonal assays, this demonstrates the utility of the single cell annotation and spatial enrichment SPEX modules for analyses of the TME and hypothesis generation[21].

SPEX workflow on spatial transcriptomics data

The availability of spatial resolution enhances the meaningful interpretation of cellular interactions and signaling events. The CLQ module calculates descriptive statistics of spatial avoidance and attraction at both the single-cell and cell cluster level. Gene and pathway expression states can be correlated with these statistics to provide insight into the effects of spatial co-occurrence on the transcriptome.

As an example, we used the CLQ method to stratify cells in a human lung cancer MERFISH sample by attraction or avoidance to each cell type (Figure 5c). We investigated how proximity to T cells modulates inflammatory and cell proliferation pathways (Figure 5d). We noticed up-regulation of NFKb pathway genes and down- regulation of Trail and TNFa genes in epithelial cells, up-regulation of EGFR genes and down-regulation of TNFa genes in myeloid cells, and down-regulation of p54 genes in endothelial cells, when split by proximity to T cells. Furthermore, the integration of the CLQ with omics data allowed us to identify genes that are differentially expressed in the proximal presence or absence of another cell type. As an example, we assessed how gene expression differs between T cells that are classified as "attractive" to or "avoidant" of Epithelial cells (as determined by CLQ analysis).

The CLQ method is limited to pairwise relations and does not take into account higher order interactions with additional cell types within potential functional communities composed of multiple cell types. We acknowledge that spatial patterns exist beyond pairwise spatial correlations and as such we introduce the next analysis module, SPEX niche analysis. This module identifies cell communities by determining the neighborhood composition for each cell in the image at a given radius and then clustering all the different neighborhoods to identify cell communities.

In the lung tumor MERFISH sample, the resulting analysis demonstrated the presence of cell type communities that define regions of interest in the image with potentially different biology. This allowed us to extract further information than the mere projection of the cell types identified by transcriptomics back into the image (Figure 5b). SPEX communities, when highlighted in the tissue slide, allow for the identification of potential structures (Figure 5e). For example, SPEX communities bring out aggregates of B cells surrounded by layer comprised of a combination of B cells, epithelial cells and T cells potentially defining TLS (Figure 5f). Beyond the spatial information, the clusters themselves can be characterized using pathway analysis. Pathways will be present as a function of the cell types involved in the community and their contextual transcriptomic state, In the present case, TGF-β has larger activity in communities containing more fibroblasts (Figure 5g).

The SPEX community analysis module can also be leveraged to look at specific cell types in the context of a particular microenvironment. More concretely, differential gene expression analyses for the same cell type across clusters can further identify context dependent expression patterns (Figure 5h). We highlight how the T cells tend to express more CD8A in SPEX community 8 (B-cell associated) compared to 10 potentially indicating a differentiation towards more cytotoxic T cells in that com-munity. Tumor cells also show some interesting patterns, when associated with stroma they express more HLA and CYR61, involved in angiogenesis. Overall, our community module generates hypotheses about regions of the tissue where potential contextual phenotypes are observed and guides the further exploration of the data.

Discussion

Cells in complex multicellular organisms are hierarchically arranged into tissues that are in turn organized into organs, and so on. At each level of organization, structure is closely related to function. In tissues, both during and after development, structure is governed by different gene expression programs that in turn depend on the resulting architecture of the cellular microenvironment experienced by the individual cells composing the tissue. This suggests that if we can measure gene and protein expression of individual cells in a given tissue and efficiently incorporate spatial cell arrangement information into these measurements, we would be able to discern the molecular basis of tissue function and dynamics.

Currently, the fast pace of the development of spatial transcriptomics and proteomics[22] is bringing us closer to fulfill this scientific program. Nevertheless, the data generated by these methodologies is very rich in information and its successful analysis and interpretation depends on the existence of computational pipelines. Most of the solutions provided by the scientific community are libraries for use by computational biologists which expose the need for a graphical user interface application to enable the direct interaction with the data from other domain experts such as pathologists or biomedical scientists. Although there are commercially licensed platforms that offer more accessible graphical interfaces, their application is restricted due to high costs, license limitations, and lack of generalizability across different modalities or platforms. This highlights the dual need for an open-source solution and a modular, easily adaptable tool.

In response to this requirement, we presented in this work, SPEX, a powerful toolkit that offers a graphical user interface for analysis of spatially resolved omics data. The unique modular design of SPEX provides flexibility, enabling users to create

bespoke data analysis pipelines. Users can directly interact with data through the graphical interface, significantly easing the process of data analysis and interpretation by eliminating the need for low-level coding.

The SPEX platform demonstrates flexibility in analyzing diverse biological data types, illustrating both its breadth of functionality and its modality-agnostic adaptability. For IMC data, we leveraged methods in SPEX to establish an end-to-end workflow to systematically characterize the spatial organization and molecular features of the highly structured microenvironment of human tonsil tissue. Additional application extended to analyzing regions of interest in a human PDAC dataset wherein we characterized TLS positioned within a heterogeneous tumor microenvironment.

Our exploration continued in other high dimensional imaging modalities where we leveraged the CLQ method to quantify local cell-cell interaction in a TNBC MIBI dataset. The outcomes were not only consistent with previously reported results by orthogonal methods but also provided novel insights into the relationship between spatial proximity and function of macrophage populations present in varying microenvironments defined by tumor-immune spatial colocalization. While these applications highlight CLQ's biological utility, practical adoption on large spatial–omics datasets also requires computational efficiency. To assess the computational performance of CLQ (Colocation Quotient), we benchmarked its execution time and memory usage alongside other commonly used cell–cell interaction scoring methods. As summarized in Supplementary Figure S1(A) and S1(B), SPEX-CLQ consistently demonstrated lower memory usage and faster processing times as the number of cells increased, compared to alternative approaches—with the exception of Squidpy. Both CLQ and the comparable method implemented in Squidpy leverage Numba, a Just-In-Time (JIT) compiler for Python that translates a subset of Python and NumPy code into fast machine code at runtime. While Squidpy exhibited greater efficiency in terms of RAM and runtime, our CLQ approach offers a unique advantage by generating a localized CLQ map, enabling spatial visualization of where attraction or avoidance patterns occur within the tissue. This added layer of spatial interpretability provides biologically meaningful insights that are not readily captured by global summary statistics alone.

Finally, we explored the ability of SPEX methods to tackle deeper data analysis tasks by analyzing a NSCLC spatial transcriptomics dataset. We integrated gene expression analysis with spatial information by leveraging the SPEX niche analysis module. We identified microenvironment domains within the tumor tissue where particular transcriptional programs are more active. Across all these use cases, SPEX showed its potential to discover and characterize informative biological programs from spatially resolved omics data.

We are aware that there are still many open questions that warrant the development of analysis modules. Our analyses indicate that "ambient RNA" diffusion in the slide is a problem of MERFISH samples that goes beyond the need to improve the cell segmentation step. This is an example of many possibilities for future development and, therefore, we hope that the scientific community will embrace this software tool and expand upon its modules.

While we foresee immediate utility of the current SPEX version in the emerging field of spatially resolved omics, we recognize the need to enhance its capabilities for the imaging processing of whole-slide imaging data and spatial transcriptomics modalities. In the forthcoming years, the field of spatially resolved omics is expected to progress towards generating multi-modal readouts, encompassing transcriptomics, proteomics, TCR and BCR repertoire, epigenomics, and metabolomics, all from the same tissue slide[22,23]. Therefore, we present SPEX, not merely as a static tool, but as an adaptable, and extensible open-source solution, directly catering to the diverse and rapidly evolving needs of the spatial omics research community. SPEX is available at 24, and has been registered in the bio.tools database under the identifier *biotools:spex*.

Methods

Data Overview

To demonstrate SPEX capabilities, we leverage both newly generated and published datasets. The published datasets include raw MIBI data for a cohort of 41 TNBC patients that was downloaded from 3.

Control Tonsil Tissue Antibody staining for imaging mass cytometry was performed on 4µm-thick, formalin-fixed, paraffin-embedded tissue sections mounted on SuperFrost PlusTM glass slides. Antibodies to cytokeratin (cat. no. ab80826), CD103 (cat. no. ab221210) and PD-L1 (cat. no. ab226766) were purchased as purified, carrier-free formulations from AbCam and, using the Maxpar X8 Antibody Labeling Kit (Fluidigm Inc., South San Francisco, CA), conjugated to 176-Yb (cat. no. 201167A), 153-Eu (cat. no. 201153A) and 150-Nd (cat. no. 201150A), respectively, per manufacturer's protocol. Remaining antibodies were purchased as conjugated from Fluidigm. To stain, tissue was first deparaffinized and rehydrated. Next, pH 9.0 antigen retrieval was used followed by incubation in Superblock at room temperature for 30 minutes. Samples were incubated with a staining panel comprised of 16 antibodies (see Supplementary table 3) overnight at 4C in a humidity chamber. Samples were washed four times in 0.1% TritonX/PBS for four minutes each followed by two 4 minute washes in PBS at room temperature. Samples were then incubated with Ir-Intercalator dye for 30 min at room temperature, washed in water for 5 min and air-dried. Regions of interest (ROIs) measuring 1800 x 2000 µm each were selected from the slide and acquired at 200 Hz. Data were exported as MCD files and visualized.

For PDAC Imaging Mass Cytometry samples, slides were first baked at 70°C for a minimum of 30 minutes to remove all visible wax. They were then subjected to a deparaffinization and rehydration series using xylenes, alcohol, and water in an autostainer. Antigen retrieval was subsequently achieved using the EZ-Retriever® System, and the slides were cooled to about 74°C in EZ-AR1 solution. After a 10-minute wash in Maxpar Water, the samples on the slide were encircled with a PAP pen and then blocked with a freshly prepared 3% BSA in Maxpar PBS solution. For antibody staining, an overnight MCA cocktail specific for the assay was prepared. The slides were incubated overnight with this mixture at 4 °C and given a secondary antibody for 30 minutes following a rinse in Maxpar PBS. Counterstaining involved washing slides with 0.2% Triton X-100 in Maxpar PBS followed by rinses in Maxpar PBS. Slides were then incubated in a diluted Intercalator-Ir solution. Finally, slides were washed in Maxpar Water, and air-dried overnight at room temperature.

MERFISH Lung Tumor Sample Samples from patients with human lung cancer were snap frozen and preserved in optimal cutting temperature (OCT) compound and cut into 10 µm thick on a cryostat at -20°C and placed on MERSCOPE Slide (Vizgen 20400001). The tissue slices were fixed with 4% paraformaldehyde in 1x PBS for 15 minutes, washed three times with 5 mL 1XPBS and incubated with 70% ethanol at 4 C overnight for tissue permeabilization. Samples were then stained for cell boundary using Vizgen's Cell Boundary Kit (10400009), and later hy-bridized with a custom designed MERSCOPE Gene Panel Mix consisting of 484 genes that assess the different cell types (tumor, stroma, immune cells, etc) and key cell signaling and activity marker (Vizgen 20300008) at 37°C incubator for 36-48 hours. Following incubation, the tissues were washed with 5 mL Formamide Wash Buffer at 47°C for 30 minutes, twice and embedded into a hydrogel using the Gel Embedding Premix (Vizgen 20300004), ammonium persulfate (Sigma, 09913-100G) and TEMED (N,N,N',N'-tetramethylethylenediamine) (Sigma, T7024-25ML) from the MERSCOPE Sample Prep Kit (10400012). After the gel mix solution solidified, the sam-ples were cleared with Clearing Solution consisting of 50uL of Protease K (NEB, P8107S) and 5mL of Clearing Premix (Vizgen 20300003) at 37°C overnight. After removing clearing solution, the sample was stained with DAPI and Poly T Reagent (Vizgen 20300021) for 15 minutes at room temperature, washed for 10 minutes with 5ml of Formamide Wash Buffer, and then im-aged on the MERSCOPE system (Vizgen 10000001). A fully detailed, step-by-step instruction on the MERFISH sample prep the full protocol is available at 25. Full Instrumentation protocol is available at 26.

SPEX modules

The SPEX graphical user interface provides essential components to facilitate comprehensive analysis of spatial omics images and datasets SPEX supports end to end modules for spatial proteomics - covering both image processing and downstream single-cell spatial analysis. Since the vast majority of single-cell spatial transcriptomics platforms output cell by transcript files with spatial coordinates, SPEX will also directly ingest a cell by transcript Anndata file (.h5ad) for downstream clustering and/or single-cell spatial analysis. For non-imaging, sequencing-based spatial transcriptomics platforms such as 10x Genomics Visium HD, users can leverage existing tools like bin2cell to transform bin-level data into pseudo single-cell resolution. The transformed data, structured as a cell-by-gene matrix with spatial coordinates, can then be converted into an AnnData object and processed by SPEX for downstream analyses such as clustering, spatial feature extraction, and colocation analysis.

Image processing

Image processing is executed in a 5 step sequence with the ultimate goal of generating a cell by expression matrix. These steps include- image loading, image preprocessing, single-cell segmenta-tion, post-processing and feature extraction. Each step contains a selection of modules which can be linked together to address the particularities of a given image set.

Image loading

The image loading step contains a single module which supports OME-TIFF or OME-ZARR. This multi-dimensional open format provides a well-structured metadata header that can accommodate a wide range of image and acquisition system information. A number of open source conversion pipelines are available in the event proprietary microscopy formats need to be converted to OMETIFF or OME-ZARR. Within SPEX, the AICSimageio python library[27] is used read these open formats in as in-memory multi-channel Numpy arrays SPEX also supports the loading of H5AD files, which are a data format for AnnData objects.

This allows SPEX users to utilize H5AD files as data sources, fter loading, this data can be processed and analyzed in the SPEX pipeline, for those modules that provide this capability.

The preprocessing step includes optional modules to denoise images and/or enhance pixel information to facilitate single-cell segmentation. These modules include- global background correction, median filter denoising, and Non-local means (NLM) denoising. All modules were developed using the Scikit-image python library. To accommodate the potentially large channel dimension of images, channels are processed in parallel with use of the apply *parallel()* function in SCIKIT image, a wrapper for DASK map *blocks()* function. This routine allows application of the denoising functions on chunked arrays which are efficiently distributed across computing cores.

In the global background correction module, background signal, as captured in one channel, can be subtracted from all other channels. For example, signal in an autofluorescent or a detector noise channel can be subtracted from channels that house specific molecular markers. Within the defined background channel, OTSU thresholding[28] is applied to create a positive pixel binary mask. This pixel mask is then intensity-scaled by a user-defined correction factor. This correction mask is then subtracted from the other channels.

Median denoising is a common image processing technique which can preserve edge information while suppressing unstructured noise. For any channel, the filter replaces a pixel's value with the median of its local neighborhood. The size of the local neighborhood is defined by the filter kernel size, an argument of the function.

Non-local means denoising provides a slightly more sophisticated method where a pixel's value is replaced by a mean which is sampled from other regions of the image. These sampled regions are only utilized if their mean is similar to the target region. This technique can preserve local texture information.

Single-cell segmentation SPEX includes several algorithms for single-cell segmentation. This includes traditional watershed cell segmentation in addition to a collection of pretrained deep learning models which include Stardist[29] and Cellpose[18]. These algorithms will operate on the channel or combination of channels containing cell nucleus information. In instances where the input data differs in resolution to the training dataset for the respective models, the user can up-sample or downsample the image pixels. All segmentation modules will return an instance segmentation label image where each cell is assigned a unique integer value.

The optional post-processing modules aim to modify the single-cell segmentation labels. Tissue-based imaging is often prone to include technical artifacts. This could be tissue tears, folds or debris. These artifacts may generate false positive cell segmentation labels. Therefore, we include rule-based functions to exclude segmented labels based on morphological features.

In some rare instances, the pretrained deep learning segmentation may include false negative regions. This is likely a result of the model not having representation of this cell type in the training data. In these cases, one can utilize the optional rescue cells() algorithm . First, prototypical cell size and intensity are calculated adaptively from the currently segmented cell objects. These parameters are then used as input for classical watershed segmentation of the image. If a label from the watershed segmentation does not overlap with that of the deep learning approach, then it is merged into the final instance segmentation label image.

The user then has an option to expand the boundaries of the cell. This may be desired since some of the cell segmentation modules utilize only nuclear information. To ensure cytoplasmic compartments are included in feature extraction, boundaries should be dilated.

SPEX utilizes the scikit image regionprops function to extract single cell features. Currently, these features include mean intensity for each channel, extracted for each cell. The output will be a cell by expression matrix in Anndata formart which serves as input for downstream cell type clustering.

Proteomics Data Clustering

An Anndata object containing the cell by expression matrix obtained from the upstream image processing modules can be clustered into groups of cells that share the same properties. The SPEX proteomics clustering module leverages Phenograph for cell typing[14]. Within this module, users will define a transformation and scaling method that is most suitable for their data. Arcsin and Log available as transformation options. Winsorizing and z-scoring are available as scaling options.

Transcriptomics Data Clustering

Cells described by a cell by expression matrix obtained from the set of image processing or spatial transcriptomics ingestion modules can be clustered by gene expression. Currently, we provide a standard method to cluster the cells using the popular scanpy package. Cells with fewer than 20 total UMI counts were excluded from analysis. The cell by expression matrix is first normalized to the median total counts and log-transformed. The matrix is then whitened and PCA is performed (the optimal number of components is determined from the singular value spectrum). A nearest-neighbor graph ($K = \sqrt{\text{number of cells}}$) is constructed on the PCA matrix and the graph is clustered using the Leiden algorithm with the default parameters.

Cell type annotation

After unsupervised clustering is executed, clusters can be renamed and grouped to facilitate more meaningful downstream spatial analysis within the SPEX application. To facilitate annotation of clusters to biologically relevant cell types, SPEX offers a manual classification module where users can easily rename cluster identifiers. The fully integrated Vitessce spatial omics visualization dashboard aids in this process by providing a means to interpret the expression profile of the cluster through heatmaps. Vitessce spatial plots can also aid in proper cluster classification as certain cell types may have specific localization patterns. One can also export the SPEX clustering results in anndata format and leverage a wide variety of cell type annotation tools available in Python or R. For analysis of the NSCLC MERFISH data, we utilized the Pegasus[30] python package for cell typing.

Spatial Analytics Module

Cell-cell co-occurrence at the cell population level To systematically assess the non-random co-occurrence/avoidance of identified cell types at the cell population level, we used a permutation test to compare the number of interactions between all of the cell types in a given image (or in a given user defined region) to that of synthetic matched controls generated under the null hypothesis containing randomized cell phenotypes. The neighborhood radius (e.g. 20 µm) was chosen empirically to restrict cell-cell co-occurrence assessment to only those cells that are close enough to potentially establish physical contact between their membranes. This approach, similar to the one described in the histoCAT paper[31], allows to determine the significance of cell-cell co-occurrence between two cell types.

Colocation Quotient: cell-cell co-occurrence at enhanced resolution The Colocation Quotient (CLQ), defined as a ratio of ratios, is a widely used geographic index[32]. In the context of tissue images, it is used to measure the local density to the global density of a specific cell type[33]. Specifically, the local density is calculated as the proportion of cell type B in the neighborhood constructed by a certain radius, centered around cell type A. The global density is the proportion of cell type B in the entire tissue image. In general, if the density of cell type B within the neighborhood of A is more than the global density of B, the CLQ will be > 1. If the neighborhood of cell type A contains many other cell types other than B, the CLQ will be < 1. The value 1 means there is no spatial relationships between the two cell types.

The CLQ can be measured globally for a particular cell type A, $\mathrm{CLQ}_{A\to B}$, or locally for each cell in the slide classified as that particular cell type, which we denote as $\mathrm{LCLQ}_{A_i\to B}$. The CLQ is calculated through the following expressions:

$$\mathrm{LCLQ}_{A_i\to B} = \frac{N_{A_i\to B}}{N_b\,(N-1)} \tag{1}$$

$$N_{A_i\to B} = \sum \frac{w_{ij}\delta_{ij}}{\sum w_{ij}} \tag{2}$$

$$\mathrm{CLQ}_{A\to B} = \frac{\sum_{i=1}^{N_A} \mathrm{LCLQ}_{A_i\to B}}{N_A} \tag{3}$$

Where N is the total number of cells in the image, δ_{ij} is the Kroenecker delta indicating whether cell j is a type B cell; w_{ij} is $1/N$ for non-weighted version, and Gaussian distance decay kernel for weighted version which is common in geostatistics application[34]. The global CLQ will then be the average of the local CLQ.

To evaluate the statistical significance of spatial associations identified by CLQ, we implemented a permutation-based hypothesis testing framework in our SPEX software, following an approach similar to that used in histoCAT for assessing cell-cell interactions in multiplex imaging data[31]. Under the null hypothesis, cell-type labels are assumed to be randomly distributed in space, implying no spatial association between cell types. To preserve tissue architecture, the spatial coordinates of all cells were kept fixed while the cell-type labels were randomly permuted across cells. For each test, 999 random permutations were performed, and the observed CLQ value was included in the null distribution, yielding a total of 1,000 values.Empirical p-values were then calculated. The p-value for spatial attraction was defined as:

$$\mathrm{P}_{attractive} = \frac{counts(CLQ_{perm} \geq CLQ_{obs}) + 1}{999+1} \tag{4}$$

and for spatial avoidant as:

$$\mathrm{P}_{avoidant} = \frac{counts(CLQ_{perm} \leq CLQ_{obs}) + 1}{999+1} \tag{5}$$

Where CLQ_{perm} denotes CLQ value calculated on each permuted sample, CLQ_{obs} denotes CLQ value calculated on the original sample. The "+1" correction ensures that the observed statistic is included in its null distribution and avoids zero p-values, consistent with best practices in non-parametric testing. Associations with $P_{\text{attractive}} < 0.05$ were considered significantly attractive, and those with $P_{\text{avoidant}} < 0.05$ were considered significantly avoidant. Other associations were classified as non-significant.

Spatially informed transcriptomics using CLQ scores For each cell type we estimated a CLQ score that describes the spatial relationship between cells (or globally as clusters), classifying them as "avoidant" or "attractive" spatial relationships. The cell-level CLQ classifications can be further investigated to identify differences in gene expression associated with for example "avoidant" behavior in clusters of interest. Gene expression differences were estimated using the scanpy (version 1.9.4)[35] using default parameters. Hypothesis-driven testing of cell clusters and cell-cell spatial relationships for differences in gene expression can be performed flexibly using SPEX.

Spatially informed transcriptomics using SPEX clusters For each cell in the dataset, neighborhood compositions were determined by collecting the counts of cells belonging to the differ-ent clusters included within a circle of radius epsilon centered on each cell (a parameter that can be adjusted by the user to assess the locality of expected cell interactions). SPEX communities then were identified via clustering of the matrix of the neighborhood compositions using Phenograph.

MERFISH single cell data was processed using the squidpy package[10] (version 1.3.0) and then fed into SPEX for spatial analyses. For each cluster identified, CLQ was calculated and neighborhood compositions were determined for each cell. SPEX communities were identified via clustering neighborhood compositions using the Leiden algorithm as implemented in scanpy (version 1.9.4). These communities were then analyzed using decoupleR[36] (version 1.4.0) to infer cell-cell interactions and pathways specific for said communities. SPEX communities were also inspected for pathway activities from a collection of well understood transcriptional pathways using progeny[37] (version 1.12.0).

Custom Analysis Modules

To accommodate the growing collection of spatial omics methods emerging in the field, SPEX supports the integration of custom plug-in modules. Users can develop their own Python-based analytical modules and integrate them into the SPEX platform for execution within a pipeline. The primary components of a custom module is (1) an *app.py* file which contains the core function - reading data, processing it and returning results and (2) a json file which defines the required parameters, pipeline execution order, dependencies and other global settings. Detailed documentation along with templates can be found on our GitHub repository[24]. This guide outlines how to define module inputs and outputs to ensure compatibility with the existing SPEX architecture.

SPEX Backend

SPEX's architecture design is mainly driven by the requirements to the system performance, availability, and scalability. In particular, we aimed for an architecture supporting multi-user access to computing resources while ensuring and guaranteeing the execution of the resource-heavy tasks set by the user in a reasonable time frame. As an additional requirement, we considered the ease of system extension and maintenance. All these requirements are fulfilled by the microservice architecture that is inherently flexible as it is based on the idea of interoperability of small, loosely coupled and easily modifiable modules (microservices). A microservice is a small program that performs a clearly assigned task. Each microservice is hosted in a Docker container and can run individually or as a group via docker-compose. SPEX microservices communicate through the data bus instrumented by Redis). Redis manages message transfer between the ser-vices ensuring messages order and guaranteed delivery even if temporary system outages occur. Message distribution and load balancing mechanisms allow to easily increase the number of the microservices' instances and spread them across different servers enabling straightforward scaling and performance boosting possibilities.

To ensure SPEX can be deployed on a variety of environments and provide recommendations for resource specifications we benchmarked execution of analysis pipelines for the reported datasets across 4 different compute environments (Fig S1 (C) and (D)). This included, Intel (CPU: Intel i5-13500, 14 cores / 20 threads, 800–4800 MHz, Memory: 62 GB RAM), AMD (CPU: AMD64 Family 25 Model 117, 3.8 GHz, 1 processor, virtualization enabled, Memory: 62 GB RAM, Virtual Memory: 97 GB total), AWS (CPU: AMD EPYC 7571, 8 cores / 16 threads, base 3.2 GHz, Memory: 62 GB RAM), and a notebook computer (CPU: AMD Ryzen 7 5800H, 8 cores / 16 threads, base 3.2 GHz, Memory: 45 GB RAM). Both processing time and memory usage for specific modules are reported. Single-cell segmentation and clustering represented the most memory intensive steps of the pipeline. This time scaled with dataset size and complexity. Segmentation is inherently memory-intensive because it involves processing large, multi-dimensional image data, often requiring the simultaneous loading of multiple image tiles or channels into memory for normalization and inference. Clustering, on the other hand, operates on large, high-dimensional single-cell feature matrices. Memory usage grows rapidly as the number of cells and features increases, since many clustering algorithms require storage of distance matrices or neighborhood graphs.

The integration of SPEX and the OMERO image management applications is implemented via a separate microservice. This service is responsible for creating OMERO sessions operating on two different layers.

The first layer, known as Python Blitz Gateway, is primarily used for direct access to images. It enables batch downloading of images in chunks (through ms-image-loader) and also permits the retrieval of image metadata not available through another layer of OMERO.The second layer, named OMERO WEB API, facilitates user interaction with images and projects. More specifically, it enables the request of metadata pertaining to user projects, accessible images, and compressed image fragments.Both layers are integral to SPEX since neither possesses all the necessary OMERO functions. Thus, to accomplish specific tasks, both layers must be used. They are created and managed by the microservice named ms-omero-session, and subsequently stored as objects in REDIS. From there, they can be accessed by other microservices and the backend system.

A proxy connection has been implemented to enable interactions with the OMERO WEB API. This mechanism proxies all requests to the OMERO WEB through our backend, utilizing a web session created on the OMERO WEB API layer by the ms-omero-session microservice. That session is then stored as an object in REDIS. This approach allows for requests to be

overridden and reused, with the ms-omero-session microservice ensuring that the session remains active and accessible when necessary.

During the initiation of image processing scripts from the pipeline, the primary layer invoked is the Python Blitz Gateway. This layer is instantiated by the microservice referred to as ms-omero-session and is then stored as an object within REDIS.The ms-job-manager microservice necessitates a Python Blitz Gateway session. This session, created and stored in REDIS by the ms-omero-session microservice at the time of user login, is persistently monitored and refreshed to ensure its availability for reuse when required.

During the execution of pipeline blocks within the microservice ms-job-manager, the computational results are converted into the anndata-zarr format, which is compatible with Vitessce[12]. These converted data are then stored in a network storage system. This storage serves as a data source for subsequent use on the client's frontend. The combination of Vitessce with data collection for creating interconnected datasets (multiple zarr files) into a unified dashboard enriches progressively with data from each pipeline step. This provides an excellent opportunity for a comprehensive overview of the entire research subject without the need to remember the full context of the experiment.

In SPEX, the data visualization system Vitessce is utilized, shifting data visualization workload from server to client side. This approach leverages cloud access capabilities within Vitessce. A key feature is the use of the H5AD to Zarr format conversion, which is compatible with Vitessce. This conversion allows for efficient data handling, as it enables users to load only the data chunks currently in use, rather than the entire dataset. This not only reduces the load on the user's device but also allows for repeated reuse of data obtained from pipeline outputs, with added filters and detail levels.

Furthermore, Vitessce is built upon WebGL2, which facilitates the transfer of data visualization processing to the graphics processing unit (GPU). Currently, Vitessce configuration files are backend-generated, offering simpler data visualization compared to seaborn and plotly. This requires initial technical expertise but ultimately reduces time in system support and development.

Ethics, consent and permissions

Tissue samples were supplied by commercial biobanks- IMC Tonsil (Bio-Options, Inc.), IMC PDAC (Indivumed) and MERFISH NSCLC (BioreclamationIVT). Each supplier received IRB approval, appropriate informed consent of all subjects, contributing biological materials, and all other authorizations, consents, or permissions as necessary for the transfer and use of the biological materials for research at Genentech. Patients/human donors were not compensated for their donation of tissues.

References

1. Dries, R. *et al.* Advances in spatial transcriptomic data analysis. *Genome Res.* **31**, 1706–1718 (2021).
2. Giesen, C. *et al.* Highly multiplexed imaging of tumor tissues with subcellular resolution by mass cytometry. *Nat. methods* **11**, 417–422 (2014).
3. Keren, L. *et al.* A structured tumor-immune microenvironment in triple negative breast cancer revealed by multiplexed ion beam imaging. *Cell* **174**, 1373–1387 (2018).
4. Goltsev, Y. *et al.* Deep profiling of mouse splenic architecture with codex multiplexed imaging. *Cell* **174**, 968–981 (2018).
5. Chen, K. H., Boettiger, A. N., Moffitt, J. R., Wang, S. & Zhuang, X. Spatially resolved, highly multiplexed rna profiling in single cells. *Science* **348**, aaa6090 (2015).
6. Palla, G., Fischer, D. S., Regev, A. & Theis, F. J. Spatial components of molecular tissue biology. *Nat. Biotechnol.* 1–11 (2022).
7. Lewis, S. M. *et al.* Spatial omics and multiplexed imaging to explore cancer biology. *Nat. methods* **18**, 997–1012 (2021).
8. Moses, L. & Pachter, L. Museum of spatial transcriptomics. *Nat. Methods* **19**, 534–546 (2022).
9. Martinelli, A. L. & Rapsomaniki, M. A. Athena: analysis of tumor heterogeneity from spatial omics measurements. *Bioinformatics* **38**, 3151–3153 (2022).
10. Palla, G. *et al.* Squidpy: a scalable framework for spatial omics analysis. *Nat. methods* **19**, 171–178 (2022).
11. Allan, C. *et al.* Omero: flexible, model-driven data management for experimental biology. *Nat. methods* **9**, 245–253 (2012).
12. Keller, M. S. *et al.* Vitessce: integrative visualization of multimodal and spatially resolved single-cell data. *Nat. Methods* **22**, 63–67 (2025).
13. Li, B., Gould, J., Yang, Y., Sarkizova, S. *et al.* Pegasus documentation. (2022). https://pegasus.readthedocs.io/en/stable/.

14. Levine, J. H. *et al.* Data-driven phenotypic dissection of aml reveals progenitor-like cells that correlate with prognosis. *Cell* **162**, 184–197 (2015).

15. Allen, C. D., Okada, T. & Cyster, J. G. Germinal-center organization and cellular dynamics. *Immunity* **27**, 190–202 (2007).

16. Bannard, O. & Cyster, J. G. Germinal centers: programmed for affinity maturation and antibody diversification. *Curr. opinion immunology* **45**, 21–30 (2017).

17. Willard-Mack, C. L. Normal structure, function, and histology of lymph nodes. *Toxicol. pathology* **34**, 409–424 (2006).

18. Stringer, C., Wang, T., Michaelos, M. & Pachitariu, M. Cellpose: a generalist algorithm for cellular segmentation. *Nat. methods* **18**, 100–106 (2021).

19. Johnson, D. B. *et al.* Quantitative spatial profiling of pd-1/pd-l1 interaction and hla-dr/ido-1 predicts improved outcomes of anti–pd-1 therapies in metastatic melanoma. *Clin. Cancer Res.* **24**, 5250–5260 (2018).

20. Tsakiroglou, A. M. *et al.* Spatial proximity between t and pd-l1 expressing cells as a prognostic biomarker for oropharyngeal squamous cell carcinoma. *Br. journal cancer* **122**, 539–544 (2020).

21. Liu, H. *et al.* Reduced cytotoxic function of effector cd8+ t cells is responsible for indoleamine 2, 3-dioxygenase-dependent immune suppression. *The J. Immunol.* **183**, 1022–1031 (2009).

22. Wu, Y., Cheng, Y., Wang, X., Fan, J. & Gao, Q. Spatial omics: Navigating to the golden era of cancer research. *Clin. Transl. Medicine* **12**, e696 (2022).

23. Duan, H., Cheng, T. & Cheng, H. Spatially resolved transcriptomics: advances and applications. *Blood Sci.* **5**, 1–14 (2023).

24. spex. Github 2025. https://github.com/Genentech/spex.

25. Vizgen. https://vizgen.com/resources/fresh-and-fixed-frozen-tissue-sample-preparation/.

26. Vizgen. https://vizgen.com/resources/merscope-instrument/.

27. Brown, E. M. *et al.* Aicsimageio: Image reading, metadata conversion, and image writing for microscopy images in pure python (2021).

28. Otsu, N. A threshold selection method from gray-level histograms. *IEEE transactions on systems, man, cybernetics* **9**, 62–66 (1979).

29. Schmidt, U., Weigert, M., Broaddus, C. & Myers, G. Cell detection with star-convex polygons. In *International Conference on Medical Image Computing and Computer-Assisted Intervention*, 265–273 (Springer, 2018).

30. Li, B. *et al.* Cumulus provides cloud-based data analysis for large-scale single-cell and single-nucleus rna-seq. *Nat. methods* **17**, 793–798 (2020).

31. Schapiro, D. *et al.* histocat: analysis of cell phenotypes and interactions in multiplex image cytometry data. *Nat. methods* **14**, 873–876 (2017).

32. Leslie, T. F. & Kronenfeld, B. J. The colocation quotient: A new measure of spatial association between categorical subsets of points. : . *Geogr. Analysis* **43**, 306–326 (2011).

33. Zhang, W. *et al.* Identification of cell types in multiplexed in situ images by combining protein expression and spatial information using celesta. *Nat. Methods* **19**, 759–769 (2022).

34. Li, X., Sun, X., Jin, L. & Xue, F. Worldwide spatial genetic structure of angiotensin-converting enzyme gene: a new evolutionary ecological evidence for the thrifty genotype hypothesis. *Eur. journal human genetics* **19**, 1002–1008 (2011).

35. Wolf, F. A., Angerer, P. & Theis, F. J. Scanpy: large-scale single-cell gene expression data analysis. *Genome biology* **19**, 1–5 (2018).

36. Badia-i Mompel, P. *et al.* decoupler: ensemble of computational methods to infer biological activities from omics data. *Bioinforma. Adv.* **2**, vbac016 (2022).

37. Schubert, M. *et al.* Perturbation-response genes reveal signaling footprints in cancer gene expression. *Nat. communications* **9**, 1–11 (2018).

38. Datasets. Github 2025. https://github.com/Genentech/spex/tree/main/demo_data/Datasets.

39. spex_common. Github 2025. https://github.com/Genentech/spex_common.

40. spex_frontend. Github 2025. https://github.com/Genentech/spex_frontend.

41. spex_backend. Github 2025. https://github.com/Genentech/spex_backend.

42. spex_bundle. Github 2025. https://github.com/Genentech/spex_bundle.

43. spex_ms_omero_image_downloader. Github 2025. https://github.com/Genentech/spex_ms_omero_image_downloader.

44. spex_ms_omero_sessions. Github 2025. https://github.com/Genentech/spex_ms_omero_sessions.

45. spex_ms_pipeline_manager. Github 2025. https://github.com/Genentech/spex_ms_pipeline_manager.

46. spex_ms_job_manager. Github 2025. https://github.com/Genentech/spex_ms_job_manager.

47. Li, X. *et al.* Spex application microservices and architecture. [computer software]. software heritage 2025. https://archive.softwareheritage.org/swh:1:snp:5b3b059d81e820de3d3609bbe5a9568c0426ca68.

48. Li, X. *et al.* Common library for spex. [computer software]. software heritage 2025. https://archive.softwareheritage.org/swh:1:snp:5108e1fa8d5b7f22c68aa7b0a89f9ccecc7f3c79.

49. Li, X. *et al.* spex_frontend. [computer software]. software heritage 2025. https://archive.softwareheritage.org/swh:1:snp:157fafab550673a06c8be00e86e417098f630833.

50. Li, X. *et al.* spex_backend. [computer software]. software heritage 2025. https://archive.softwareheritage.org/swh:1:snp:c606fd111c1572ba959eb9ebac54da90183b419c.

51. Li, X. *et al.* omero server. [computer software]. software heritage 2025. https://archive.softwareheritage.org/swh:1:snp:8491c334f65df99cb4777b45b94ab0772bbbb8c3.

52. Li, X. *et al.* Microservice splitter. [computer software]. software heritage 2025. https://archive.softwareheritage.org/swh:1:snp:a32447690ddd1f513e8a4b455bed8764de26a787.

53. Li, X. *et al.* spex_ms_omero_sessions. [computer software]. software heritage 2025. https://archive.softwareheritage.org/swh:1:snp:5bf64eabbfb93b946892ae924b38100037a597aa.

54. Li, X. *et al.* Microservice collector. [computer software]. software heritage 2025. https://archive.softwareheritage.org/swh:1:snp:b07899251f6cc02f5da3722f2316c8f79b3b18d6.

55. Li, X. *et al.* spex_ms_job_manager. [computer software]. software heritage 2025. https://archive.softwareheritage.org/swh:1:snp:dd43d2f4525454d5a2b8d760ad76487c12ca3a40.

56. Li, X. *et al.* Supporting data for "spex: A modular end-to-end platform for high-plex tissue spatial omics analysis" gigascience database 2025. https://doi.org/10.5524/102723.

Figures

Figure 1. Graphical depiction of the Spatial Expression Explorer (SPEX) platform and analytical workflow. (a) Data can be input into SPEX as images or single-cell objects. Image data is managed and served into SPEX via the OMERO image management system. **(b)** Analytical projects in SPEX are executed as a 4 step process - create project, build pipeline, batch execute and review results. Projects are containers for input data, editable pipelines and study output data. **(c)** A modular pipeline builder is used to assemble an analytical routine. Modules are selected from the module library, covering image processing, segmentation, clustering, spatial analysis and expression analysis. The individual modules are shown as multi-colored rounded squares and can be added to the pipeline in a graphical manner. **(d)** Analysis data generated by the pipeline are stored as an Anndata object. As the pipeline proceeds, new elements are added to the Anndata object. **(e)** Processed datasets packaged as Anndata ZARR files can be reviewed using an integrated Vitessce dashboard. **(f)** Output data is compiled as an Anndata ZARR can be exported for downstream analysis in a variety of platforms.

Figure 2. SPEX Graphical User Interface. (a) SPEX project creation user interface. Projects are containers for study data. **(b)** SPEX-OMERO integration highlighting navigation and selection OMERO hosted datasets and images within SPEX. **(c)** SPEX pipeline builder user interface. An intuitive 3 step process of building, executing and reviewing the analysis displayed at the top of the interface. The user moves through each step in sequence. In the build section the user is able to graphically build an analysis pipeline by selecting and chaining modules in sequence. A unique parameter section is displayed for each module. **(d)** Fully integrated Vitessce dashboard for visualizing results in spatial context.

Figure 3. SPEX robustly identified structural composition of Tonsil and PDAC tissue in Imaging Mass Cytometry modality. **(a)** Graphical dataset description and SPEX spatial proteomics workflow modules highlighted in this figure **(b)** UMAP embedding of Tonsil single-cell data colored by pathologist annotated cell type. Inset shows primary high order Tonsil structures (EPI=Epithelial, GC = Germinal Center, MZ/PC= Marginal Zone/Paracortex). **(c)** Detail of the Germinal Center region of UMAP with pathologist annotated functional zones. **(d)** Protein expression of Ki67 and CD68 color mapped to UMAP embedding and spatial domain. Warm colors denote high expressing regions **(e)** The histologic tonsil image is color-coded by 14 structural hierarchy categories identified by the marker expression pattern. **(f)** PDAC single-cell clustering expression heatmap **(g)** Cell type composition of 4 individual IMC ROIs **(h)** Spatial map of clustered cell types across 4 PDAC ROIs **(i)** Multiplex IMC image showing detail of Tertiary Lymphoid Structure (TLS). CD3= Green, CD20 = Red, CD21=Cyan **(j)** Detail spatial map of cell types in a TLS region **(k)** Spatial distribution expression heatmap of CD21, CD20, and CD3e.

Figure 4. SPEX spatial analysis modules reveal immune cell state relationships to tumor:immune spatial architecture in a TNBC MIBI dataset. **(a)** Graphical dataset description and SPEX spatial proteomics workflow modules highlighted in this figure. **(b)** Representative image of TNBC patient tumor from the Keren cohort showing color overlays of DNA (white), PanCytokeratin (PanCK, blue), CD20 (green), CD8 (yellow), CD11b (pink), CD68 (cyan) and Vimentin (VIM, red), scale bar = 100 um. **(c)** Cell phenotype map corresponding to B showing 13 major cellular phenotypes by colored points. **(d)** Frequencies of major cell lineages (of all cells, top) and immune cell types (of total immune cells, bottom) are shown as stacked bar plots for each patient. **(e)** Scatterplot showing results of SPEX CLQ analysis module camparing the immune::tumor spatial enrichment vs tumor:immune spatial enrichment in each patient that had more than 10% immune cells (not desert). Based on these data patients are assigned to an immune-inflamed (orange) class or an immune-excluded (blue) class using a GMM model. **(f)** Heatmaps showing the tumor:immune, tumor:tumor, immune:tumor, and immune:immune CLQ results in immune-excluded patients versus immune-inflamed patients. **(g)** Representative cell phenotype maps of two immune-desert tumors, two immune-excluded tumors, and two immune-inflamed tumors, showing the location of tumor (blue), immune (red), mesenchymal (brown), endothelial (green), and other (grey) cells. **(h)** A cell phenotype map (top) showing cytotoxic T cells (blue) and macrophages (green) is shown above a heatmap of the macrophage cells, colored by their CLQ Macrophage:Tcell spatial enrichment score. **(i)** Boxplot showing the normalized expression of PDL1 in macrophages in immune-desert, immune-excluded, and immune-inflamed tumors, asterisks denote significance in kruskal-wallis test, **** $P < 0.001$, ns = not significant. Boxplot showing the normalized expression of IDO1 in macrophages that are either CD8-Tcell-avoidant (CLQ < 0.5) or CD8-Tcell-attractive (CLQ> 1.5) in immune-excluded vs immune-inflamed tumors, Mann-whitney test, asterisks denote significance: ** $P < 0.01$, **** $P < 0.001$.

Figure 5. SPEX spatial transcriptomics analysis identifies immune cell niches. **(a)** Graphical dataset description and SPEX spatial transcriptomics workflow modules highlighted in this figure. **(b)** Cell phenotype map in both gene expression (UMAP, left) and spatial (right) coordinates. **(c)** PROGENy pathway enrichment scores for each cell type cluster, split by colocalization with T cells. Colocalization was determined using the SPEX CLQ analysis module. Scores were calculated by fitting a multilinear model to the gene expression of each cell given the pathway weights per gene. The average score per cluster is shown here. **(d)** Volcano plot showing differentially expressed genes for T cells based on colocalization with epithelial cells. **(e)** Spatial map of cell niches. **(f)** Cell phenotype composition of detected niches with detail spatial maps showing phenotype spatial arrangement in niches. **(g)** PROGENy pathway enrichment scores for each spatial niche. **(h)** Volcano plot showing differential expression between T cells in spatial niche 10 vs spatial niche 8 (B-cell enriched) (left) and volcano plot showing differential expression between epithelial cells in spatial niche 9 (macrophage enriched) vs spatial niche 1 (right).

Author contributions statement

X.L., X.P., C.F. T.R., D.O., and R.J. wrote the manuscript with input from all authors. X.L., X.P. , D.O. and R.J. conceived the SPEX platform. D.O. and R.J. supervised the project (D.O. from 2020 to 2022, and R.J. and J.G. 2022-present upon D.O.'s departure from Genentech). J.G. also provided software deployment strategy. R.J. designed the UI with input from authors and end users, developed image processing methods and executed image processing on spatial proteomics datasets. X.L., X.P., C.F., D.O. developed spatial analysis methods. X.P., D.H., and C.F. developed code for spatial transcriptomics analysis and executed analysis of spatial transcriptomics datasets. C.C. developed and tested single-cell data structure schema. T.R. analyzed and interpreted MIBI data. S.R., P.C., J.Z. executed method development of IMC PDAC data. D.O. performed clustering analysis of IMC Tonsil data. F.P. analyzed and interpreted IMC Tonsil spatial data. Z.S , M.N., and X.Y. generated Lung MERFISH data and executed primary analysis. L.M. provided interpretation and validation of spatial methods. J.C. and J.S. provided infrastructure engineering and OMERO integration work. H.C.B. provided software development supervision. A.Z. and E.T. executed frontend and backend software development. A.P. executed frontend and backend software development in addition to software testing. Both X.L. and X.P. contributed equally and have the right to list their names first in their CVs. All authors read and approved the final manuscript.

Availability of supporting source code and requirements

Project name: SPEX (Spatial Expression Explorer)
Project home page: https://github.com/Genentech/SPEX
Operating system(s): Linux, Windows
Programming language: Python, Javascript
Other requirements: Docker, Git, Git LFS, modern web browser (Chrome or Firefox), 64-bit x86 system
License: Apache License, Version 2.0
RRID: SCR_026970
bio.tools ID: biotools:spex

Data Availability

Datasets used and/or analyzed in this work are made available at 38. The software code is available from github repository[24,39–46]. Snapshots of the code are available in Software Heritage[47–55]. All additional datasets used in this research can be found in GigaDB[56].

Competing interests

X.L., X.P., D.O. and R.J. are co-inventors on a provisional patent application filed by Genentech/Roche relating to this manuscript.

Author Affiliations

All authors were employees of Genentech at the time of project contributions. X.L.'s current affiliation is Roche Diagnostic Solutions (xiao.li.xl2@roche.com). D.O.'s current affiliation is Cell Signaling Technologies (darya.orlova@cellsignal.com). X.P.'s current affiliation is Revolution Medicines (xpechuanjorge@revmed.com). A.Z. and E.T. were contract employees at time of project contribution. A.Z.'s current email address is artzub@gmail.com and E.T.'s current email is e.tabatsky@gmail.com. All other authors are current employees of Genentech.

Supporting Information

Table 1. Comparison of SPEX with existing spatial omics tools.

**Tool/Package**	**Squidpy**	**Giotto**	**MCmicro**	**Aquilla**	**EZSingleCell**	**SPEX**
GUI or code-based execution	Code-based: Python library	Code-based: R	Code-based: Nextflow	Code-free: Web application	Code-free: Web application	Code-free: Web application
Target Audience & Prerequisites	Python experience required	R coding experience required	Coding experience required	No coding experience needed	No coding experience needed	No coding experience needed
Data types supported	multi-channel TIFF, OME-TIFF, ZARR, Anndata	S4 object system in R	OMETIFF (Image), CSV	PostgreSQL (ROI annotation, cell/spot coordination, gene expression)	CSV, TSV, 10x Cell Ranger/Space Ranger/Cell Ranger-ATAC output (.H5)	OMETIFF (Image), OMEZARR (Image), Anndata (single-cell object)
Interactive Image (pixels) visualization	Napari-interactive, multi-dimensional image viewer in Python	Interactive viewer on the user's local computer	MINERVA downstream integration	N/A	Low-resolution Space Ranger output	Integrated Image Viewer
Image Preprocessing	Image cropping, subsetting, filtering, normalization	N/A	Image Alignment	N/A	N/A	Median Filter, Background Subtraction
Single-cell segmentation	Watershed, Cellpose, StarDist, Custom (skimage/OpenCV)	Mesmer	uMINST	N/A	N/A	Cellpose, Stardist, Watershed
Cell Type Clustering	Leiden, Louvain, GMM, SingleR, scVI	N/A	N/A	N/A	Seurat, CellID, Celltypist	Phenograph
Spatial analysis	Spatial graphs, Moran's I, Geary's C, co-occurrence, neighborhood enrichment, spatial variability, ligand-receptor analysis	Spatial enrichment, deconvolution, coherent expression, neighborhood analysis	SCIMAP platform	Cell-cell interaction, neighborhood analysis, spatial entropy, spatial variable genes	GraphST	CLQ Cell-Cell Interaction, Niche/Neighborhood Analysis
Image Data management	ImageContainer (wrapper of xarray.Dataset), Lazy loading, Metadata storage	N/A	N/A	N/A	N/A	OMERO Integration
Data Visualization	Matplotlib, Seaborn, Scanpy's plotting functions	R data visualization libraries	MINERVA	Web-based	R Shiny Web Application	Vitessce Integration

Table 2. SPEX Analysis Modules

Category	Module
I/O	Load Tiff<br>Load Image
Image Preprocessing	Median Denoising<br>NLM Denoising<br>Background Subtraction
Image Segmentation	Watershed<br>Stardist<br>Cellpose
Segmentation Post-processing	Remove Small Objects<br>Remove Large Objects<br>Simulate Cell<br>Rescue Cells
Clustering (Proteomics)	Feature Extraction<br>Phenograph
Clustering (Transcriptomics)	Preprocessing<br>Scanpy Clustering
Transcriptomics Secondary Analysis	Differential Expression Analysis<br>Pathway Analysis
Spatial Analysis	Colocation Quotient (CLQ)<br>Niche Analysis

Table 3. Antibodies, conjugates, clones and working concentrations used in tonsil multiplex staining

Antibody	Conjugate	Clone	Concentration (μg/mL)
CD20	161-Dy	H1	3
CD45	152-Sm	D9M8I	7
CD68	159-Tb	KP1	0.1
CD8	162-Dy	CD8/144B	2
Collagen type I	169-Tm	Polyclonal	1
Cytokeratin	176-Yb	AE1/AE3	0.8
Histone H3	171-Yb	D1H2	0.5
Ki67	168-Er	B56	3
BCL2	146-Nd	EPR17509	7
CD25	175-Lu	EPR6452	5
CD31	151-Eu	EPR3094	5
CD336/Tim3	154-Sm	D5D5R	7
PD1	165-Ho	EPR4877	5
PDL1	150-Nd	73-10	10

Figure S1. Pipeline and method Benchmarking.(A) Memory and (B) Time of CLQ algorithm against other cell-cell interaction methods with increasing cell number. CLQ benchmarking utilized 60GB RAM and 8 cores (C) Memory usage in megabytes and (D) processing time in seconds for each analytical module (x-axis) utilized in the SPEX analysis pipeline for IMC Tonsil, IMC PDAC, MIBI TNBC, and MERFISH NSCLC respectively. Each datapoint corresponds to a single input and is colored by the compute environment utilized. The IMC PDAC dataset consisted of 4 images and MIBI TNBC consisted of 41 images processed as a batch in the SPEX application. IMC Tonsil and MERFISH NSCLC had 1 sample each. Pipeline benchmarking utilized the following systems: Intel (CPU: Intel i5-13500, 14 cores / 20 threads, 800–4800 MHz, Memory: 62 GB RAM), AMD (CPU: AMD64 Family 25 Model 117, 3.8 GHz, 1 processor, virtualization enabled, Memory: 62 GB RAM, Virtual Memory: 97 GB total), AWS (CPU: AMD EPYC 7571, 8 cores / 16 threads, base 3.2 GHz, Memory: 62 GB RAM), and a notebook computer (CPU: AMD Ryzen 7 5800H, 8 cores / 16 threads, base 3.2 GHz, Memory: 45 GB RAM)

fig. 1
Click here to access/download;Figure;Figure1.pdf

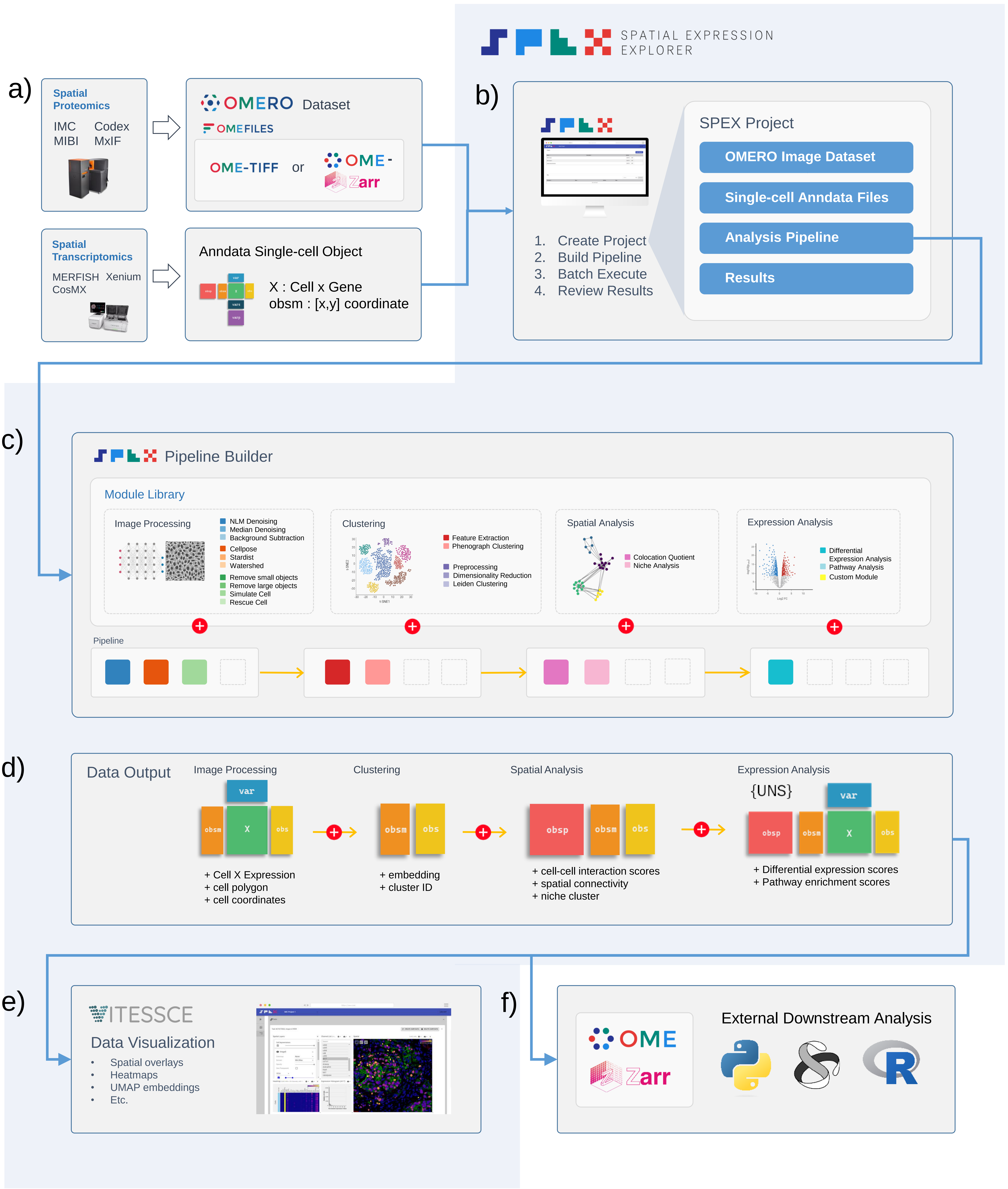

fig. 2

Click here to access/download;Figure;Figure2.pdf

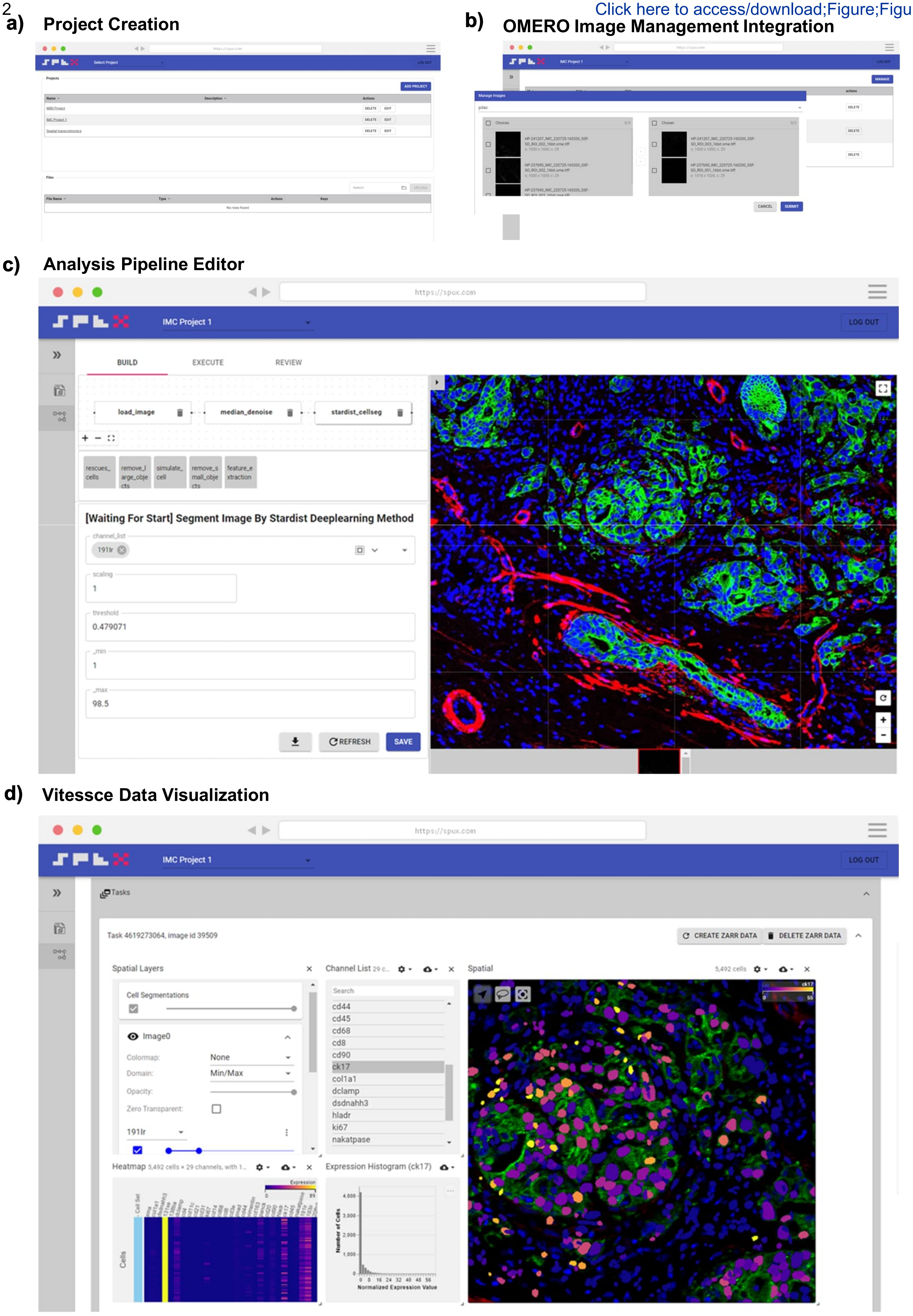

fig. 3

Click here to access/download;Figure;Figure3.pdf

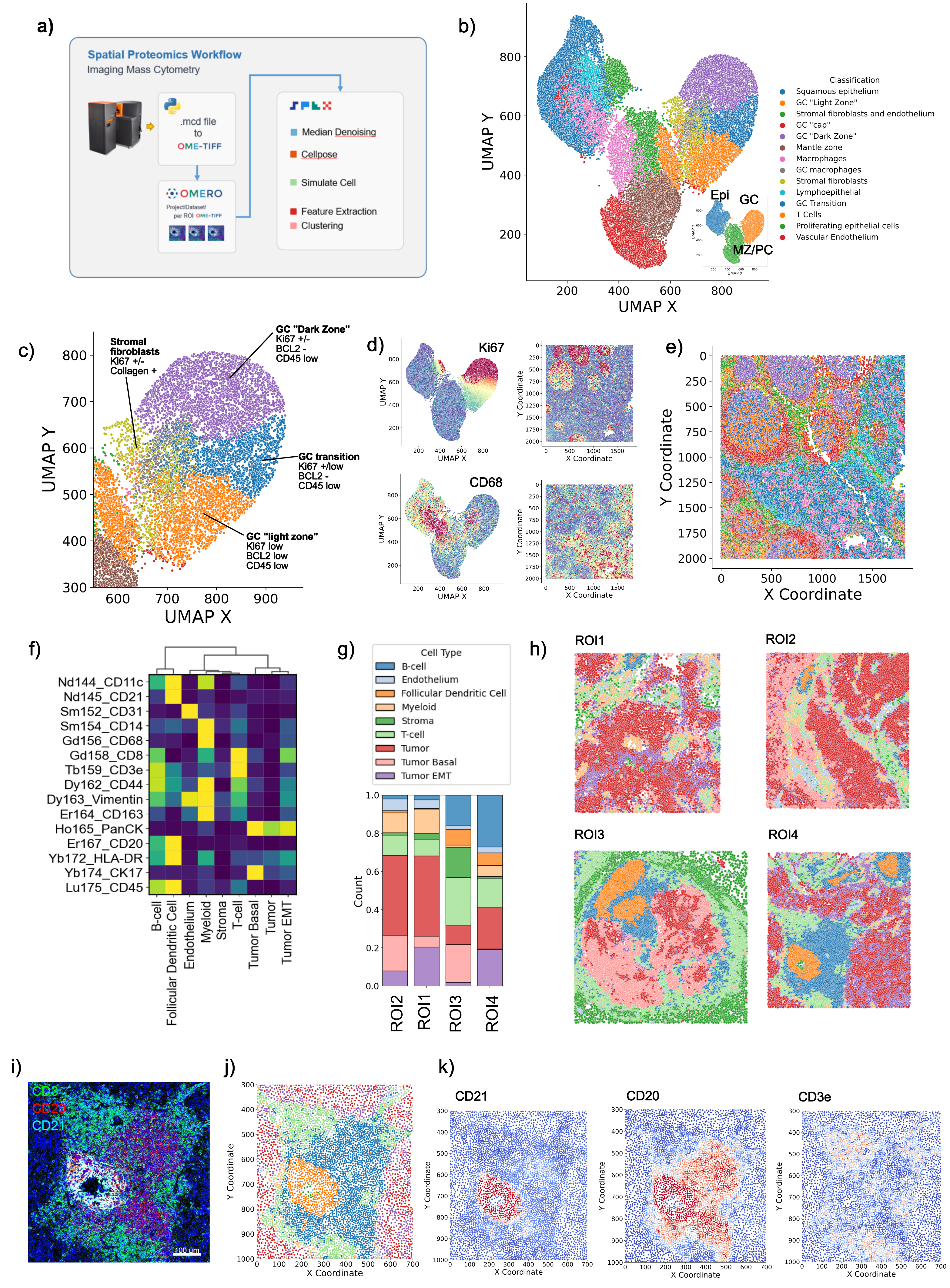

fig. 4

Click here to access/download;Figure;Figure4.pdf

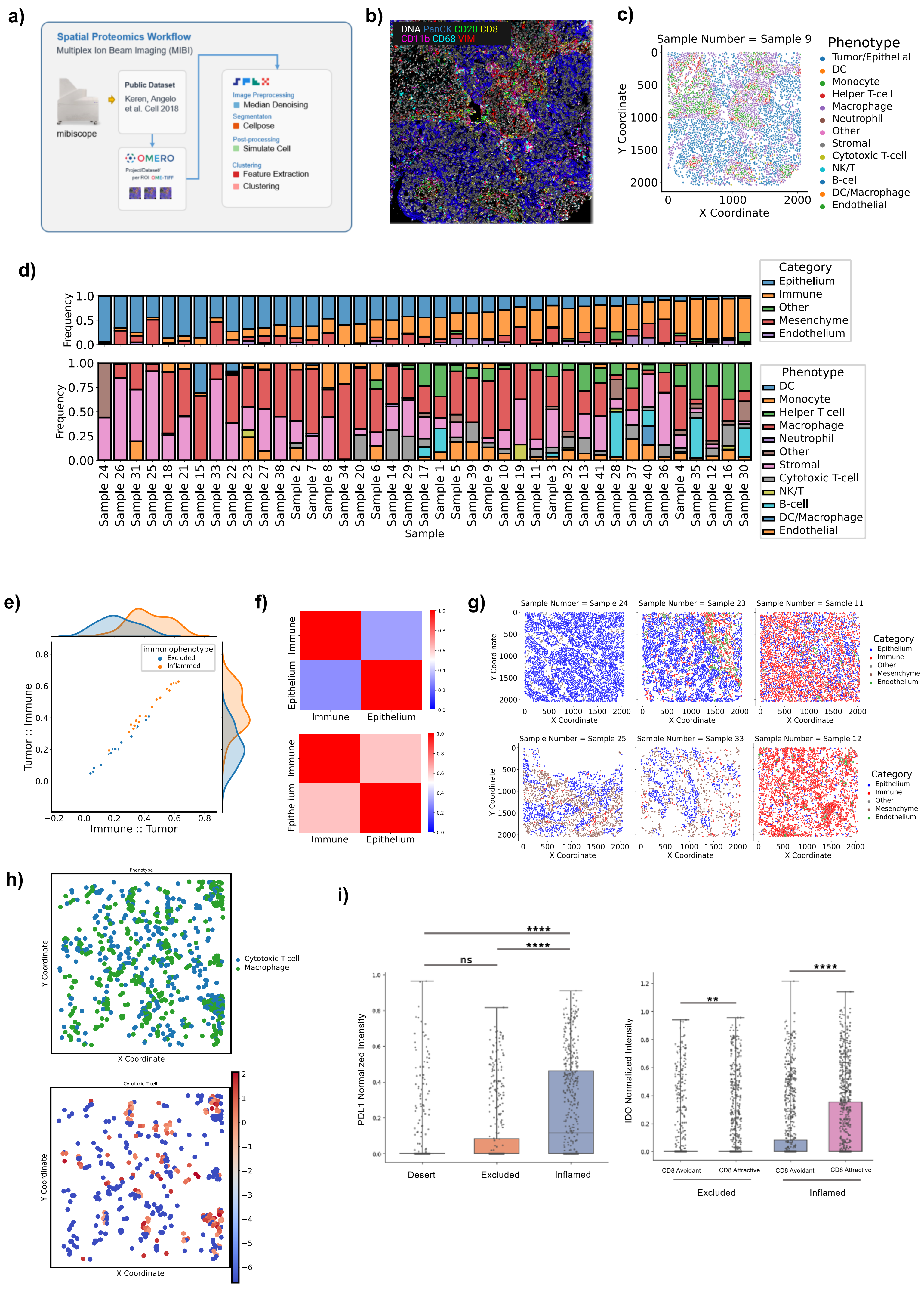

fig. 5

Click here to access/download;Figure;Figure5.pdf

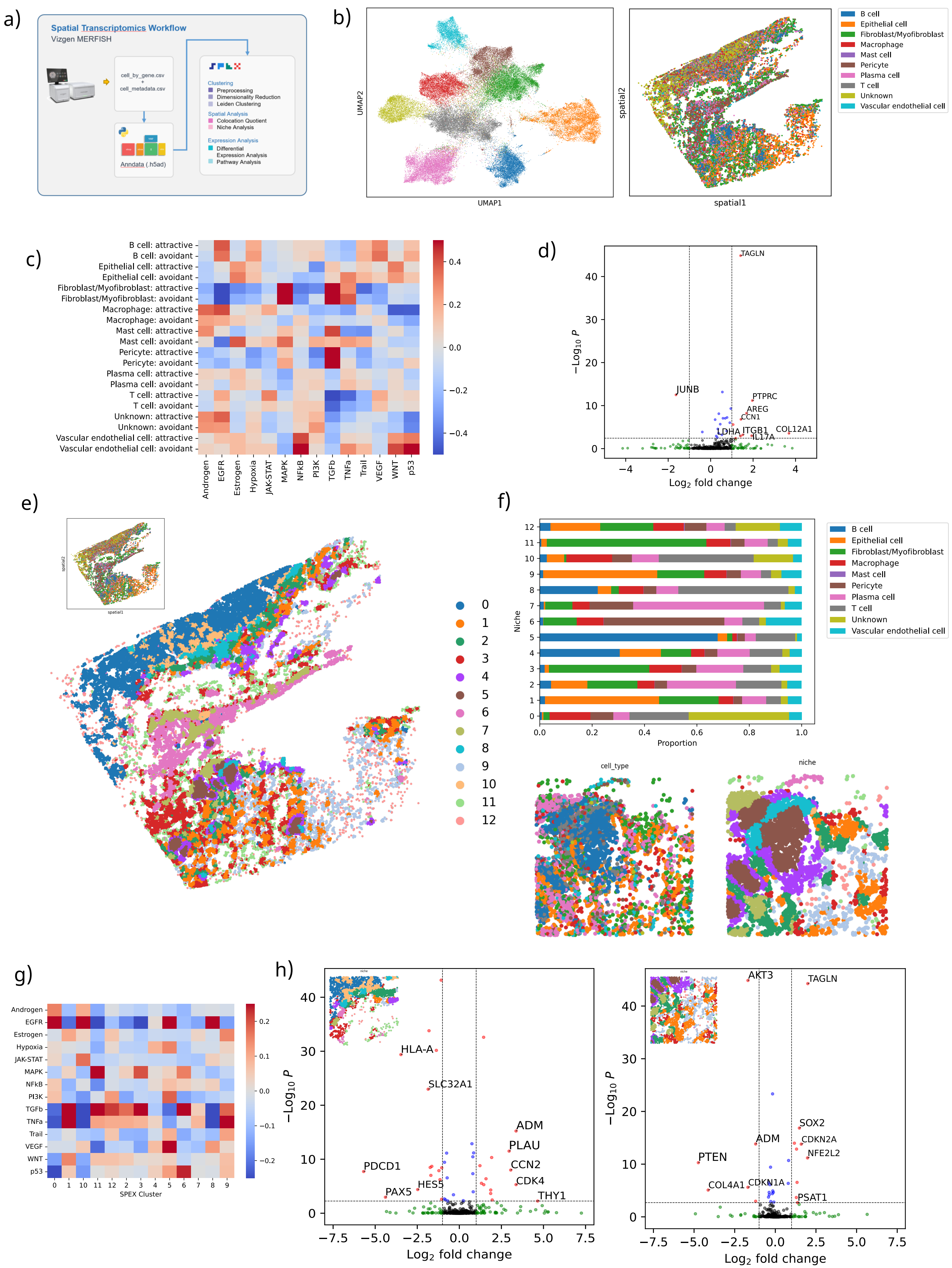

Reviewer comments and responses

Click here to access/download
Supplementary Material
GigaScience_SPEX_ReviewerComments.pdf

Presubmission correspondance

Click here to access/download
Supplementary Material
GigaScience_Presubmission_correspondance.pdf

fig. s1

Click here to access/download
Supplementary Material
FigureS1.pdf

Cover Letter with suggested Reviewers

Click here to access/download;Personal Cover;CoverLetter_GigaScience_SPEX.pdf

Genentech
A Member of the Roche Group

January 15, 2025

GigaScience Press, BGI Hong Kong Tech Co Ltd.,
26F A Kings Wing Plaza 2, 1 On Kwan Street,
Shek Mun, Sha Tin, NT, Hong Kong SAR

Dear Editor,

Please consider our manuscript entitled "SPEX: A modular end-to-end analytics tool for tissue spatial omics analysis" by X. Li, X. Pechuan-Jorge, T. Risom, C. Foo, A. Zubkov, C. Chan, P. Chang, F. Peale, J. Ziai, S. Rost, D. Hibar, L. McGinnis, E. Tabatsky, X. Ye, H.C. Bravo, Z. Shi, M. Nowicka, J. Scherdin, J. Cowan, J. Giltnane, D. Orlova, and R. Jesudason. We believe that reviewers will find this manuscript highly suitable for GigaScience as a Technical Report..

Recent developments in transcriptomics and proteomics have opened the possibility for the molecularly resolved spatial characterization of cellular composition in tissues, promising a deeper understanding of tissue biology in both homeostasis and disease. The wealth of data generated by these technologies has driven the development of new tools that often require advanced coding fluency or pay-for-service analytical platforms and contract research organizations. Additionally, new methods frequently address only a narrow scope of the full analytical process required to translate raw imaging data into actionable insights. Moreover, many of these methods do not scale to the typical data size of spatial omics studies, limiting their practical utility.

To enable data analysis capabilities for the larger community, we present SPEX (Spatial Expression Explorer), an open-source, comprehensive single-cell spatial analysis pipeline implemented as a user-friendly web-based application. The platform features analytical modules that can be conveniently chained together as pipelines through a graphical user interface. SPEX's infrastructure allows for streamlined access to open-source image data management systems (OMERO) and analysis modules for image denoising, cell segmentation, cell-cell co-occurrence, local cell type communities characterization, and spatially informed omics analyses. Additionally, SPEX utilizes open single-cell data structures, facilitating integration with other workflows and ensuring compatibility with a wide range of data formats. Furthermore, SPEX integrates seamlessly with popular open-source omics visualization platforms (Vitessce), enhancing the accessibility and interpretability of the results.

We demonstrate SPEX's ability to facilitate the discovery of biological insights from a variety of spatially resolved omics datasets. Importantly, SPEX's open-source model allows for both commercial and academic use under an Apache license.

We trust that our manuscript will be of great interest to the audience of GigaScience. We thank you in advance for your time and effort in considering our submission. Below, you will find a list of suggested reviewers.

Sincerely,

Rajiv Jesudason
Computational Scientist, Team Lead, Digital Pathology Image Analysis- Spatial Omics, Research Pathology
Genentech, A Member of the Roche Group
Office +1 (650) 123-4567
jesudasr@gene.com

Genentech
A Member of the Roche Group

Suggested Reviewers:

Abdulhamit Subasi
Institute of Biomedicine, Faculty of Medicine, University of Turku, Turku, Finland
https://scholar.google.com/citations?hl=en&user=W6FLhskAAAAJ&view_op=list_works&sortby=pubdate
Email: abdulhamit.subasi@utu.fi

Semyon Kolmykov,
Sirius University, Krasnodar region, Russia
https://scholar.google.ru/citations?user=Xl80wLUAAAAJ&hl=ru
Email: kolmykov.sk@talantiuspeh.ru

Qiwei Li
Department of Mathematical Sciences, The University of Texas at Dallas, Richardson, Texas, USA
https://sites.google.com/site/liqiwei2000/
Email: qiwei.li@utdallas.edu

Rong Fan
Department of Biomedical Engineering and Department of Pathology, Yale School of Medicine, New Haven, CT, USA
https://seas.yale.edu/faculty-research/faculty-directory/rong-fan
Email: rong.fan@yale.edu

Denis Antonets
https://scholar.google.ru/citations?hl=ru&user=Gpn-G6QAAAAJ
State Research Center of Virology and Biotechnology "VECTOR", Federal Service for Surveillance on Consumer Rights Protection and Human Wellbeing, Koltsovo, Novosibirsk region, Russia
Email: antonec@nprog.ru
Email: antonec@vector.nsc.ru

Veniamin Fishman
Institute of Cytology and Genetics, Novosibirsk, Russia
Skoltech Center for Stem Cell Research, Skolkovo Institute of Science and Technology, Skolkovo 143025, Moscow, Russia
https://assa.icgbio.ru/open/person/782/
https://scholar.google.ru/citations?hl=ru&user=b72SdFUAAAAJ
Email: minja@bionet.nsc.ru
