# Supplementary material for: SPEX: A modular end-to-end platform for high-plex tissue spatial omics analysis
Source: Gigascience. 2025 Aug 29;14:giaf090. doi: 10.1093/gigascience/giaf090 (PMC12395962; doi:10.1093/gigascience/giaf090)
Supplement: giaf090_Authors_Response_To_Reviewer_Comments_Original_Submission [file giaf090_authors_response_to_reviewer_comments_original_submission.pdf]

Author's Response To Reviewer Comments

[Reviewer responses are attached as a pdf document and also listed point by point here]

Dear Editorial Board,
Below are our responses to the reviewers' comments. We sincerely appreciate the reviewers' thoughtful and thorough evaluation. We have addressed all concerns to the best of our ability and look forward to your feedback.
For your convenience, we have highlighted the changes in the revised manuscript in blue.
Reviewer #1:

Comment: The reviewer would like to request clarifications on how extensible the modules are. The author mentioned a SPEX pipeline builder in which "modules are selected from a library and dragged into a visual pipeline map", and also mentioend the support for "flexible plug-in analysis modules".What are the packages available from the library? Can users import their own code or script or package? How to create new plug-in's?
Response:
In response to the reviewer’s request, we have added Supplementary Table 2, SPEX Analysis Modules, which lists all currently implemented algorithms, categorized by their intended use (e.g., segmentation, clustering, etc.).
Each module within the SPEX user interface is backed by a corresponding Python script. As an open-source platform, SPEX provides developers with full access to these scripts, allowing them to modify and extend functionalities as needed. This open framework ensures that SPEX can continuously evolve in step with the latest advancements in spatial omics analysis.
SPEX also supports the integration of fully custom modules. Users can develop their own Python-based analytical modules and seamlessly integrate them into the SPEX platform. To facilitate this, we have added detailed documentation to our GitHub repository (https://github.com/Genentech/SPEX?tab=readme-ov-file#project-structure), including step-by-step instructions and a template for creating custom modules. This guide outlines how to define module inputs and outputs to ensure compatibility with the existing SPEX architecture. Additionally, we have updated the manuscript to include a more comprehensive description of the plug-in process.
“Custom Analysis Modules
To accommodate the growing collection of spatial omics methods emerging in the field, SPEX supports the integration of custom plug-in modules. Users can develop their own Python-based analytical modules and integrate them into the SPEX platform for execution within a pipeline. The primary components of a custom module is (1) an app.py file which contains the core function - reading data, processing it and returning results and (2) a json file which defines the required parameters, pipeline execution order, dependencies and other global settings. Detailed documentation along with templates can be found on our GitHub repository (https://github.com/Genentech/SPEX?tab=readme-ov-file#project-structure). This guide outlines how to define module inputs and outputs to ensure compatibility with the existing SPEX architecture.“
Comment: The reviewer is also wondering how do the users interact with the results? Can the user click on the resulting image and select regions of interest to zoom in?
Response:
SPEX supports interactive visualization through seamless integration with the open-source platform Vitessce (Keller et al, 2024), which enables dynamic exploration of spatial data. Users can interact directly with the resulting images, for example, by clicking to select regions of interest, which can then be zoomed in for more detailed inspection. This facilitates focused analysis of specific tissue areas. Vitessce also supports cross-filtering between the image view, UMAP projections, and bar plots. These interactive features enhance the exploratory capabilities of SPEX, allowing users to gain deeper insights from their spatial omics data.
To highlight this functionality of SPEX, we have added the following statement to our manuscript:
“Visualization
The final step in the pipeline building interface is data visualization. Here, users can visualize analytical readouts using an integrated Vitessce spatial omics dashboard. Vitessce, as described by Keller et al.

(2024), is an advanced web-based platform designed for the interactive visualization of spatial omics data. The dashboard integrates a variety of visualization modalities, such as spatial plots, scatterplots, and expression heatmaps, histograms and more. All visualization panes are interactive and have cross-filtering functionality (Figure 2d). As an example, a user can select a specific population of cells in the UMAP embedded space and these same cells will be highlighted in the spatial plot atop the multiplex imaging data. The image pane can be zoomed and panned to further explore the tissue microenvironment. Together, these plots facilitate investigation of spatial patterns, cell clustering, and tissue organization at single-cell resolution. "

Reviewer #2:

Comment: The manuscript presents an advancement in spatial omics analysis but needs improvements in Quantitative benchmarking, Computational scalability assessment, etc. With these revisions, SPEX has the potential to become a widely adopted platform in the spatial omics community. I have specific comments as below:
While the manuscript provides a qualitative comparison of SPEX with other spatial omics tools (e.g., Squidpy, Giotto, Aquilla), quantitative benchmarking is missing. It is needed to include a performance benchmark comparing runtime efficiency, segmentation accuracy, and clustering resolution against existing tools. Also, it is necessary to show computational efficiency metrics (e.g., memory usage, execution time, scalability across datasets of varying sizes)

Response:

We thank the reviewer for their insightful comments and fully agree on the importance of quantitative benchmarking in evaluating analytical tools. In the supplementary materials, we have included Figure S1 that presents performance benchmarks across several datasets analyzed using SPEX. This figure highlights key metrics such as runtime efficiency, memory usage, and scalability across the diverse datasets described in the manuscript.

We would like to emphasize that the primary goal of SPEX is not to compete with existing segmentation, clustering, or other analytical algorithms, but rather to address a key gap in the spatial omics field—the limited availability of user-friendly, GUI-based tools that enable end-to-end analysis of spatially resolved omics data. We recognize that the most appropriate choice of segmentation, clustering, or other algorithms is often task- and dataset-specific. Therefore, our aim is to provide a flexible platform where users can easily plug in the algorithms of their choice and operate them within a GUI-based environment. Please refer to the 'GUI or code-based' field of Supplementary Table 1, which defines how far a user without coding expertise can progress using currently available platforms.
While SPEX includes and supports a set of default algorithms-each of which has been benchmarked in the original studies where they were introduced-we would like to reiterate that these are provided primarily for illustrative purposes. However, we have now included a justification for the specific algorithms selected as defaults. The following statements have been added to the manuscript: "For proteomics, we also include the widely adopted graph-based clustering algorithm, PhenoGraph, which has recently been shown to be among the most robust to the curse of dimensionality compared to other high-dimensional clustering methods14."
In the case of CLQ, while its global version has been previously studied, for example, in the CELESTA framework developed by Zhang, Weiruo, et al. (2022), its local variant has not yet been explored in this context. Therefore, we conducted a side-by-side benchmarking of the global CLQ versions, and our results show that its performance is comparable to the current state of the art. Importantly, we have also introduced a local CLQ, which is unique; by design, it outperforms the global CLQ by enabling the investigation of heterogeneity in patterns that would otherwise remain undetectable at the average cell population level. These results are now included in the supplementary materials.
We have also updated the manuscript with the following description of the plug-in process:
"Custom Analysis Modules
To accommodate the growing collection of spatial omics methods emerging in the field, SPEX supports the integration of custom plug-in modules. Users can develop their own Python-based analytical modules and integrate them into the SPEX platform for execution within a pipeline. The primary components of a custom module are (1) an app.py file which contains the core function which covers reading input data, processing

it and returning results and (2) a json file which defines the required interactive parameters, pipeline execution order, dependencies and other global settings. Detailed documentation along with templates can be found on our GitHub repository (https://github.com/Genentech/SPEX?tab=readme-ov-file#project-structure). This guide outlines how to define module inputs and outputs to ensure compatibility with the existing SPEX architecture."

Comment: The study presents compelling results, but there is no independent validation or interpretation of computational outputs using experimental methods.

Response:

We thank the reviewer for this important comment. This manuscript focuses on introducing a new software analysis package and demonstrating its utility in enabling biological discovery. While some of the included results are indeed biologically compelling, validating them experimentally is beyond the scope of this work. To address this, we have revised the manuscript to soften the language regarding the ability to draw definitive biological conclusions based solely on the computational outputs. We have also more explicitly stated that analysis outcomes were evaluated for robustness either through comparison with prior analyses of the same dataset (e.g., Keren et al.) or, in cases where independent analyses were not available, through expert review by experienced pathologists (e.g., IMC data).

Comment: The manuscript does not discuss hardware requirements, processing speed, or computational limitations. It is needed to provide an assessment of SPEX's performance on different computing environments (e.g., local workstations vs. cloud computing vs. high-performance clusters).

Response:

We agree with the reviewer's suggestion and have now incorporated the relevant information into the manuscript. We have included a benchmarking of pipeline execution for all reported datasets using several computing environments which include, AWS, INTEL, AMD, and local notebook execution. We have also included the resource specification details for each environment in the manuscript. Suggested minimal system requirements have been added to the application github repository https://github.com/Genentech/SPEX. The following paragraph has been added to the manuscript:

"To ensure SPEX can be deployed on a variety of environments and provide recommendations for resource specifications we benchmarked execution of analysis pipelines for the reported datasets across 4 different compute environments (Fig S1 (C) and (D)). This included, Intel (CPU: Intel i5-13500, 14 cores / 20 threads, 800–4800 MHz, Memory: 62 GB RAM), AMD (CPU: AMD64 Family 25 Model 117, ~3.8 GHz, 1 processor, virtualization enabled, Memory: 62 GB RAM, Virtual Memory: 97 GB total), AWS (CPU: AMD EPYC 7571, 8 cores / 16 threads, base ~3.2 GHz, Memory: 62 GB RAM), and a notebook computer (CPU: AMD Ryzen 7 5800H, 8 cores / 16 threads, base 3.2 GHz, Memory: 45 GB RAM). Both processing time and memory usage for specific modules are reported. Single-cell segmentation and clustering represented the most memory intensive steps of the pipeline. This time scaled with dataset size and complexity. Segmentation is inherently memory-intensive because it involves processing large, multi-dimensional image data, often requiring the simultaneous loading of multiple image tiles or channels into memory for normalization and inference. Clustering, on the other hand, operates on large, high-dimensional single-cell feature matrices. Memory usage grows rapidly as the number of cells and features increases, since many clustering algorithms require storage of distance matrices or neighborhood graphs."

Comment: The Colocation Quotient (CLQ) method is well described, but the manuscript does not provide statistical validation (e.g., p-values, confidence intervals) for detected spatial relationships.

Response:

We sincerely thank the reviewer for highlighting the importance of statistical validation for the Colocation Quotient (CLQ) method.

In SPEX implementation, we have implemented permutation-based hypothesis testing to evaluate the significance of spatial associations. Below are the key details of our approach:

Permutation Testing Framework:

Null Hypothesis: Spatial associations between cell types are random (i.e., no systematic attraction/avoidant).

Procedure:
Spatial coordinates of cells were fixed to preserve tissue architecture.
Cell type labels were randomly shuffled across cells (e.g. 999 permutations) to simulate spatial randomness under the null hypothesis.
The observed CLQ was included in the null distribution, resulting in a total of 1,000 values(999 permutations + observed CLQ).
Empirical P-value Calculation:
P-values were computed as:
Pattractive=count(permuted CLQs>=observed CLQ)+1/(1+999)
Pavoidant=count(permuted CLQs<=observed CLQ)+1/(1+999)
This "+1" adjustment ensures that the observed CLQ is part of its own null distribution, preventing p-values of zero and aligning with best practices in non-parametric testing.
Significance Thresholds:
Attractive: Pattractive 0.05 (observed CLQ ≥ 95% of permuted CLQs).
Avoidant: Pavoidant 0.05 (observed CLQ ≤ 95% of permuted CLQs).
Relationships not meeting these thresholds were classified as non-significant.

We also updated the method section to strengthen the statistical rigor of our analysis.

Reviewer #3:

Comment: One of the critical omissions in this manuscript is the lack of rigorous benchmarking against established tools. Though it demonstrated the comparison with other tools such as Squidpy, Giotto, and MC Micro, but there is no quantitative comparison to demonstrate its advantages over existing methodologies. In particular, spatial analysis such as CLQ is introduced as a different approach within the spatial biology analytics framework, but how does it compare to existing co-occurrence analysis methods? Additionally, similar analyses have been conducted using other tools (e.g., Mah, C.K., et al., Genome Biol 25, 82 (2024)), including in 'subcellular' colocalization. In this regard, concerns about its novelty arise. Moreover, as mentioned in relation to Bento, CLQ could also be applied to subcellular analysis?
- In this regard, for spatial co-occurence or other algorithms in SPEX, the authors should run identical datasets through both SPEX and existing tools to compare performance and biological insights. it is impossible to assess whether SPEX provides any meaningful improvement over existing platforms.

Response:

We would like to emphasize that the primary goal of SPEX is not to compete with existing segmentation, clustering, or other analytical algorithms, but rather to address a key gap in the spatial omics field—the limited availability of user-friendly, GUI-based tools that enable end-to-end analysis of spatially resolved omics data. We recognize that the most appropriate choice of segmentation, clustering, or other algorithms is often task- and dataset-specific. Therefore, our aim is to provide a flexible platform where users can easily plug in the algorithms of their choice and operate them within a GUI-based environment. Please refer to the 'GUI or code-based' field of Supplementary Table 1, which defines how far a user without coding expertise can progress using currently available platforms.
While SPEX includes and supports a set of default algorithms-each of which has been benchmarked in the original studies where they were introduced-we would like to reiterate that these are provided primarily for illustrative purposes. However, we have now included a justification for the specific algorithms selected as defaults. The following statements have been added to the manuscript: "For proteomics, we also include the widely adopted graph-based clustering algorithm, PhenoGraph, which has recently been shown to be among the most robust to the curse of dimensionality compared to other high-dimensional clustering methods [https://www.biorxiv.org/content/10.1101/2024.04.18.589981v1]."
In the case of CLQ, while its global version has been previously studied, for example, in the CELESTA framework developed by Zhang, Weiruo, et al. (2022), its local variant has not yet been explored in this context. Therefore, we conducted a side-by-side benchmarking of the global CLQ versions, and our results show that its performance is comparable to the current state of the art. Importantly, we have also introduced a local CLQ, which is unique; by design, it outperforms the global CLQ by enabling the

investigation of heterogeneity in patterns that would otherwise remain undetectable at the average cell population level. These results are now included in the supplementary materials.
We have also updated the manuscript with the following description of the plug-in process:
"Custom Analysis Modules
To accommodate the growing collection of spatial omics methods emerging in the field, SPEX supports the integration of custom plug-in modules. Users can develop their own Python-based analytical modules and integrate them into the SPEX platform for execution within a pipeline. The primary components of a custom module is (1) an app.py file which contains the core function - reading data, processing it and returning results and (2) a json file which defines the required parameters, pipeline execution order, dependencies and other global settings. Detailed documentation along with templates can be found on our GitHub repository (https://github.com/Genentech/SPEX?tab=readme-ov-file#project-structure). This guide outlines how to define module inputs and outputs to ensure compatibility with the existing SPEX architecture."

Comment: The cell typing process is one of the most fundamental steps in spatial omics analysis. However, SPEX does not integrate a dedicated cell typing module, forcing users to use another tool or define cell types manually. The accuracy of all downstream analyses (clustering, spatial interaction, pathway analysis) depends on robust and reliable cell typing. It would be better to integrate with automated cell typing solutions to increase usability.
Response:
We thank the reviewer for highlighting the importance of cell typing in spatial omics analysis and for pointing out the need for more explicit clarification in our manuscript. After unsupervised clustering is executed, clusters can be renamed and grouped to facilitate more meaningful downstream spatial analysis within the SPEX application. To facilitate annotation of clusters to biologically relevant cell types, SPEX offers a manual classification module where users can easily rename cluster identifiers. The fully integrated Vitessce spatial omics visualization dashboard aids in this process by providing a means to interpret the expression profile of the cluster through heatmaps. Vitessce spatial plots can also aid in proper cluster classification as certain cell types may have specific localization patterns. One can also export the SPEX clustering results in anndata format and leverage a wide variety of cell type annotation tools available in Python or R. For analysis of the NSCLC MERFISH data, we utilized the Pegasus python package for cell typing.
We do agree that a fully integrated automated cell typing routine would be beneficial, therefore we again note that users have flexibility in implementing algorithms from established packages like Pegasus or CellTypist as customized plugins. This design empowers users to identify and incorporate the most appropriate approach for their data.
To address the reviewer's concern, we have expanded the description of the cell annotation process in the manuscript and added Supplementary Table 2, which provides a comprehensive overview of all currently implemented algorithms in SPEX, categorized by their intended use (e.g., segmentation, phenotyping, etc.). We believe this addition will help clarify how users can perform or incorporate cell typing workflows within the SPEX framework.
Comment: The manuscript focuses almost exclusively on single-cell resolution data and high-dimensional imaging-based methods (e.g., IMC, MIBI, MERFISH). However, spot-based transcriptomics platforms such as Visium are widely used in the field. In this regard, SPEX does not provide modules tailored methodology for spot-based spatial analysis (such as deconvolution) or super-resolution or transforming cell-based analysis from spots (e.g. bin2cell in VisiumHD). Neighborhood analyses or spatially variable gene detection, etc. are specialized in whole-gene covered, spot-based methods, as well, for example.

Response:
We thank the reviewer for this important observation. Indeed, the current version of SPEX has been primarily designed and benchmarked using single-cell resolution and high-dimensional imaging-based datasets. However, we agree that spot-based transcriptomics platforms such as Visium are widely used and require tailored analytical approaches.
While SPEX does not currently include built-in modules for cell-type deconvolution or spatial super-resolution, it can be used downstream of preprocessing tools such as bin2cell, which convert spot-level data into approximated single-cell resolution. Once such transformation is performed, users can leverage SPEX's existing suite of modules for neighborhood analysis, clustering, and spatial pattern detection.
We recognize the importance of expanding SPEX's capabilities to natively support spot-based analyses and

plan to explore integration of such functionality in future releases. To clarify this limitation and the potential workaround, we have revised the manuscript to include the following statement:
“SPEX modules
The SPEX graphical user interface provides essential components to facilitate comprehensive analysis of spatial omics images and datasets SPEX supports end to end modules for spatial proteomics - covering both image processing and downstream single-cell spatial analysis. Since the vast majority of single-cell spatial transcriptomics platforms output cell by transcript files with spatial coordinates, SPEX will also directly ingest a cell by transcript Anndata file (.h5ad) for downstream clustering and/or single-cell spatial analysis. For non-imaging, sequencing-based spatial transcriptomics platforms such as 10x Genomics Visium HD, users can leverage existing tools like bin2cell to transform bin-level data into pseudo single-cell resolution. The transformed data, structured as a cell-by-gene matrix with spatial coordinates, can then be converted into an AnnData object and processed by SPEX for downstream analyses such as clustering, spatial feature extraction, and colocation analysis‟
Comment: The manuscript does not clarify whether users can modify or extend the pipeline with custom Python scripts. Describing further this point, customization in this ecosystem with python script, for 'power-users' of this system could be helpful.
Response:
We thank the reviewer for this helpful suggestion. Each module within the SPEX user interface is backed by an underlying Python script. As an open-source platform, SPEX provides developers with full access to these scripts, enabling them to modify and extend functionalities as needed. This openness ensures that SPEX can continually evolve alongside advancements in spatial omics analysis. SPEX also supports the integration of fully custom modules. Users can develop their own Python-based analytical components and seamlessly incorporate them into the platform. To facilitate this, we have added detailed documentation to our GitHub repository, including step-by-step instructions and a template for creating custom modules. This guide walks users through the process of defining module inputs and outputs to ensure compatibility with the existing SPEX architecture. We have now incorporated a statement reflecting this functionality into the manuscript.

Comment: The biological relevance of the SPEX platform remains unclear, as the case studies presented are not sufficiently rigorous. As mentioned above, comparisons with other tools based on quantification can clarify why SPEX is better than other published tools/ecosystems in certain aspect. Or meaningful biological findings and explanations based on this tool as a case study could be helpful. While the results demonstrate technical capabilities, the manuscript does not show how SPEX enables novel biological discoveries compared to existing tools.

Response:

We thank the reviewer for these thoughtful suggestions and for recognizing that the results presented in our manuscript adequately demonstrate the technical capabilities of the SPEX platform. While presenting a novel biological discovery is beyond the scope of this manuscript, we have now emphasized the strong agreement between SPEX analysis results and previously published data (Keren et al.).
Please find the following statement under “SPEX Spatial Proteomics Pipeline Enables Novel Single Cell Spatial Insights” subsection in the “Results” section:

“Following cell phenotyping by unsupervised clustering these SPEX modules generated cell calls that correctly match their molecular profiles and the published calls by the original work3 (Figure 4b-d). Through arranging the tumors from low to high immune cell frequency of total cells, we observe an increased diversity of immune cell types within the sample where samples with a lower density of immune cells are dominated by macrophages and samples with higher density of immune cells have a mixed composition of lymphocytes, myeloid cells and antigen presenting cells (Figure 4d). To further interrogate the spatial relationship between this variable immune compartment and the tumor cells, we employ the Colocation Quotient (CLQ) method
under the SPEX Spatial co-occurrence module. Briefly, the CLQ measures the co-occurrence or avoidance of cell type pairs by looking at the local density of a target cell type at a fixed radius from each cell of the sample belonging to a reference cell type. That way, one can, for instance, query if target immune cells are co-locating or avoiding tumor cells. We applied the CLQ module with the immune cell::tumor cell pair here in samples that had more than 10% immune cells (non-desert samples) revealing two major groups of

samples, those with high immune::tumor cell spatial enrichment, and those with low immune:tumor cell enrichment (Figure 4e). These identified inflamed and excluded groupings aligned well with the visual representation of immunophenotyping presented in the images (Figure 4f)."

We would like to emphasize that the primary goal of SPEX is not to compete with existing segmentation, clustering, or other analytical algorithms, but rather to address a key gap in the spatial omics field—the limited availability of user-friendly, GUI-based tools that enable end-to-end analysis of spatially resolved omics data. We recognize that the most appropriate choice of segmentation, clustering, or other algorithms is often task- and dataset-specific. Therefore, our aim is to provide a flexible platform where users can easily plug in the algorithms of their choice and operate them within a GUI-based environment. Please refer to the 'GUI or code-based' field of Supplementary Table 1, which defines how far a user without coding expertise can progress using currently available platforms.

While SPEX includes and supports a set of default algorithms-each of which has been benchmarked in the original studies where they were introduced-we would like to reiterate that these are provided primarily for illustrative purposes. However, we have now included a justification for the specific algorithms selected as defaults. The following statements have been added to the manuscript: "For proteomics, we also include the widely adopted graph-based clustering algorithm, PhenoGraph, which has recently been shown to be among the most robust to the curse of dimensionality compared to other high-dimensional clustering methods [https://www.biorxiv.org/content/10.1101/2024.04.18.589981v1]."

In the case of CLQ, while its global version has been previously studied, for example, in the CELESTA framework developed by Zhang, Weiruo, et al. (2022), its local variant has not yet been explored in this context. Therefore, we conducted a side-by-side benchmarking of the global CLQ versions, and our results show that its performance is comparable to the current state of the art. Importantly, we have also introduced a local CLQ, which is unique; by design, it outperforms the global CLQ by enabling the investigation of heterogeneity in patterns that would otherwise remain undetectable at the average cell population level. These results are now included in the supplementary materials.

We have also updated the manuscript with the following description of the plug-in process:

"Custom Analysis Modules

To accommodate the growing collection of spatial omics methods emerging in the field, SPEX supports the integration of custom plug-in modules. Users can develop their own Python-based analytical modules and integrate them into the SPEX platform for execution within a pipeline. The primary components of a custom module is (1) an app.py file which contains the core function - reading data, processing it and returning results and (2) a json file which defines the required parameters, pipeline execution order, dependencies and other global settings. Detailed documentation along with templates can be found on our GitHub repository (https://github.com/Genentech/SPEX?tab=readme-ov-file#project-structure). This guide outlines how to define module inputs and outputs to ensure compatibility with the existing SPEX architecture."

Editorial Manager®

Cookie Preference Center

We use cookies which are necessary to make our site work. We may also use additional cookies to analyze, improve and personalize our content and your digital experience. For more information, see our Cookie Policy.

You may choose not to allow some types of cookies. However, blocking some types may impact your experience of our site and the services we are able to offer. See the different category headings below to find out more or change your settings.

Allow all

Manage Consent Preferences

Strictly Necessary Cookies

Always active

These cookies are necessary for the website to function and cannot be switched off in our systems. They are usually only set in response to actions made by you which amount to a request for services, such as setting your privacy preferences, logging in or filling in forms. You can set your browser to block or alert you about these cookies, but some parts of the site will not then work.

Functional Cookies

These cookies enable the website to provide enhanced functionality and personalisation. They may be set by us or by third party providers whose services we have added to our pages. If you do not allow these cookies then some or all of these services may not function properly.

Performance Cookies

These cookies allow us to count visits and traffic sources so we can measure and improve the performance of our site. They help us to know which pages are the most and least popular and see how visitors move around the site.

Targeting Cookies

These cookies may be set through our site by our advertising partners. They may be used by those companies to build a profile of your interests and show you relevant adverts on other sites. If you do not allow these cookies, you will experience less targeted advertising.

Cookie List

Clear

Apply Cancel

Consent Leg.Interest

Confirm my choices

Powered by onetrust
