# Supplementary material for: SPEX: A modular end-to-end platform for high-plex tissue spatial omics analysis
Source: Gigascience. 2025 Aug 29;14:giaf090. doi: 10.1093/gigascience/giaf090 (PMC12395962; doi:10.1093/gigascience/giaf090)
Supplement: giaf090_Reviewer_1_Report_Original_Submission — Ka Yee Yeung, Ph.D. -- 3/9/2025 [file giaf090_reviewer_1_report_original_submission.pdf]

Reviewer Report

Title: SPEX: A modular end-to-end platform for high-plex tissue spatial omics analysis

Version: Original Submission **Date:** 3/9/2025

Reviewer name: Ka Yee Yeung, Ph.D.

Reviewer Comments to Author:

Li et al. presented SPEX (Spatial Expression Explorer), a web-based open-source end-to-end analysis platform offering modular design and a user accessible interface. The users demonstrated use cases in spatial transcriptomics (MERFISH lung cancer) and spatial proteomics datasets (tonsil, public multiplex ion beam imaging data). SPEX includes the following analytical modules1. image processing modules includes a 4-step sequence (image pre-processing, single-cell segmentation, post-processing, feature selection). Image loading supports OMERO integration. Output is a cell by expression matrix in Anndata format.2. clustering modules for both spatial transcriptomic and proteomic data.3. spatial analysis module implements the CLQ (Colocation Quotient) method.4. spatial expression analysis module includes differential expression and pathway analysis.SPEX supports visualization via Vitessce.The paper is well written, addresses a rising interest and critical need in the biomedical community. The reviewer would like to request clarifications on how extensible the modules are. The author mentioned a SPEX pipeline builder in which "modules are selected from a library and dragged into a visual pipeline map", and also mentioend the support for "flexible plug-in analysis modules".What are the packages available from the library? Can users import their own code or script or package? How to create new plug-in's?The reviewer is also wondering how do the users interact with the results? Can the user click on the resulting image and select regions of interest to zoom in?

Level of Interest

Please indicate how interesting you found the manuscript: Choose an item.

Quality of Written English

Please indicate the quality of language in the manuscript: Choose an item.

Declaration of Competing Interests

Please complete a declaration of competing interests, considering the following questions:

- Have you in the past five years received reimbursements, fees, funding, or salary from an organisation that may in any way gain or lose financially from the publication of this manuscript, either now or in the future?
- Do you hold any stocks or shares in an organisation that may in any way gain or lose financially from the publication of this manuscript, either now or in the future?

- Do you hold or are you currently applying for any patents relating to the content of the manuscript?
- Have you received reimbursements, fees, funding, or salary from an organization that holds or has applied for patents relating to the content of the manuscript?
- Do you have any other financial competing interests?
- Do you have any non-financial competing interests in relation to this paper?

If you can answer no to all of the above, write 'I declare that I have no competing interests' below. If your reply is yes to any, please give details below.

No to 1, 2, 3, 4, 6 5. Yes. Ka Yee Yeung-Rhee is a a co-founder and equity holder in Biodepot LLC.

I agree to the open peer review policy of the journal. I understand that my name will be included on my report to the authors and, if the manuscript is accepted for publication, my named report including any attachments I upload will be posted on the website along with the authors' responses. I agree for my report to be made available under an Open Access Creative Commons CC-BY license (http://creativecommons.org/licenses/by/4.0/). I understand that any comments which I do not wish to be included in my named report can be included as confidential comments to the editors, which will not be published.

Choose an item.

To further support our reviewers, we have joined with Publons, where you can gain additional credit to further highlight your hard work (see: https://publons.com/journal/530/gigascience). On publication of this paper, your review will be automatically added to Publons, you can then choose whether or not to claim your Publons credit. I understand this statement.

Yes Choose an item.
