# Supplementary material for: SPEX: A modular end-to-end platform for high-plex tissue spatial omics analysis
Source: Gigascience. 2025 Aug 29;14:giaf090. doi: 10.1093/gigascience/giaf090 (PMC12395962; doi:10.1093/gigascience/giaf090)
Supplement: giaf090_Reviewer_3_Report_Original_Submission — Hongyoon Choi -- 3/11/2025 [file giaf090_reviewer_3_report_original_submission.pdf]

Reviewer Report

Title: SPEX: A modular end-to-end platform for high-plex tissue spatial omics analysis

Version: Original Submission **Date:** 3/11/2025

Reviewer name: Hongyoon Choi

Reviewer Comments to Author:

The manuscript introduces SPEX, a web-based platform designed for spatial omics data analysis. The authors highlight its user-friendly UI, modular analysis pipelines, and integration with open-source image data management systems. The platform supports image processing such as cell/nucleus segmentation, clustering, and spatial analysis. GUI-based approaches as well as python script-based modules increase usability for the broader research community.While the goals of the platform are commendable, and the integration of multiple analysis modules is a valuable contribution, there are critical shortcomings in the manuscript that must be addressed before publication.Several key weaknesses significantly limit the scientific rigor and impact of this work.- One of the critical omissions in this manuscript is the lack of rigorous benchmarking against established tools. Though it demonstrated the comparison with other tools such as Squidpy, Giotto, and MC Micro, but there is no quantitative comparison to demonstrate its advantages over existing methodologies. In particular, spatial analysis such as CLQ is introduced as a different approach within the spatial biology analytics framework, but how does it compare to existing co-occurrence analysis methods? Additionally, similar analyses have been conducted using other tools (e.g., Mah, C.K., et al., Genome Biol 25, 82 (2024)), including in 'subcellular' colocalization. In this regard, concerns about its novelty arise. Moreover, as mentioned in relation to Bento, CLQ could also be applied to subcellular analysis?- In this regard, for spatial co-occurence or other algorithms in SPEX, the authors should run identical datasets through both SPEX and existing tools to compare performance and biological insights. it is impossible to assess whether SPEX provides any meaningful improvement over existing platforms.- The cell typing process is one of the most fundamental steps in spatial omics analysis. However, SPEX does not integrate a dedicated cell typing module, forcing users to use another tool or define cell types manually. The accuracy of all downstream analyses (clustering, spatial interaction, pathway analysis) depends on robust and reliable cell typing. It would be better to integrate with automated cell typing solutions to increase usability.- The manuscript focuses almost exclusively on single-cell resolution data and high-dimensional imaging-based methods (e.g., IMC, MIBI, MERFISH). However, spot-based transcriptomics platforms such as Visium are widely used in the field. In this regard, SPEX does not provide modules tailored methodology for spot-based spatial analysis (such as deconvolution) or super-resolution or transforming cell-based analysis from spots (e.g. bin2cell in VisiumHD). Neighborhood analyses or spatially variable gene detection, etc. are specialized in whole-gene covered, spot-based methods, as well, for example.- The manuscript does not clarify whether users can modify or extend the pipeline with custom Python scripts. Describing further this point, customization in this ecosystem with python script, for 'power-users' of this system could be helpful.- The biological relevance of the SPEX platform remains unclear, as the case studies presented are not sufficiently rigorous. As mentioned above,

comparisons with other tools based on quantification can clarify why SPEX is better than other published tools/ecosystems in certain aspect. Or meaningful biological findings and explanations based on this tool as a case study could be helpful. While the results demonstrate technical capabilities, the manuscript does not show how SPEX enables novel biological discoveries compared to existing tools.

Level of Interest

Please indicate how interesting you found the manuscript: Choose an item.

Quality of Written English

Please indicate the quality of language in the manuscript: Choose an item.

Declaration of Competing Interests

Please complete a declaration of competing interests, considering the following questions:

- Have you in the past five years received reimbursements, fees, funding, or salary from an organisation that may in any way gain or lose financially from the publication of this manuscript, either now or in the future?
- Do you hold any stocks or shares in an organisation that may in any way gain or lose financially from the publication of this manuscript, either now or in the future?
- Do you hold or are you currently applying for any patents relating to the content of the manuscript?
- Have you received reimbursements, fees, funding, or salary from an organization that holds or has applied for patents relating to the content of the manuscript?
- Do you have any other financial competing interests?
- Do you have any non-financial competing interests in relation to this paper?

If you can answer no to all of the above, write 'I declare that I have no competing interests' below. If your reply is yes to any, please give details below.

I declare that I have no competing interests

I agree to the open peer review policy of the journal. I understand that my name will be included on my report to the authors and, if the manuscript is accepted for publication, my named report including any attachments I upload will be posted on the website along with the authors' responses. I agree for my report to be made available under an Open Access Creative Commons CC-BY license (http://creativecommons.org/licenses/by/4.0/). I understand that any comments which I do not wish to be included in my named report can be included as confidential comments to the editors, which will not be published.

Choose an item.

To further support our reviewers, we have joined with Publons, where you can gain additional credit to further highlight your hard work (see: https://publons.com/journal/530/gigascience). On publication of this paper, your review will be automatically added to Publons, you can then choose whether or not to claim your Publons credit. I understand this statement.

Yes Choose an item.
